# Inhibition of G-protein signalling in cardiac dysfunction of intellectual developmental disorder with cardiac arrhythmia (IDDCA) syndrome

**DOI:** 10.1136/jmedgenet-2020-107015

**Published:** 2020-11-10

**Authors:** Pasquelena De Nittis, Stephanie Efthymiou, Alexandre Sarre, Nicolas Guex, Jacqueline Chrast, Audrey Putoux, Tipu Sultan, Javeria Raza Alvi, Zia ur Rahman, Faisal Zafar, Nuzhat Rana, Fatima Rahman, Najwa Anwar, Shazia Maqbool, Maha S Zaki, Joseph G Gleeson, David Murphy, Hamid Galehdari, Gholamreza Shariati, Neda Mazaheri, Alireza Sedaghat, SYNAPS Study Group, Gaetan Lesca, Nicolas Chatron, Vincenzo Salpietro, Marilena Christoforou, Henry Houlden, William F Simonds, Thierry Pedrazzini, Reza Maroofian, Alexandre Reymond

**Affiliations:** 1 Center for Integrative Genomics, University of Lausanne, Lausanne, Switzerland; 2 Department of Neuromuscular Disorders, Queen Square Institute of Neurology, University College London, London, UK; 3 Cardiovascular Assessment Facility, University of Lausanne, Lausanne, Switzerland; 4 Bioinformatics Competence Center, University of Lausanne, Lausanne, Switzerland; 5 Service de Génétique, Hopital Femme Mere Enfant, Bron, France; 6 Department of Pediatric Neurology, The Children's Hospital and Institute of Child Health, Lahore, Pakistan; 7 Department of Paediatric Neurology, Children's Hospital and Institute of Child Health, Multan, Pakistan; 8 Department of Developmental-Behavioural Paediatrics, The Children's Hospital and Institute of Child Health, Lahore, Pakistan; 9 Clinical Genetics Department, Human Genetics and Genome Research Division, National Research Centre, Cairo, Egypt; 10 Department of Neuroscience and Pediatrics, Howard Hughes Medical Institute, La Jolla, California, USA; 11 Department of Genetics, Faculty of Science, Shahid Chamran University of Ahvaz, Ahwaz, Iran (the Islamic Republic of); 12 Department of Medical Genetics, Faculty of Medicine, Ahvaz Jondishapour University of Medical Sciences, Ahvaz, Iran (the Islamic Republic of); 13 Health Research Institute, Diabetes Research Center, Ahvaz Jundishapur University of medical Sciences, Ahvaz, Iran (the Islamic Republic of); 14 Service de Genetique, Hospices Civils de Lyon, Lyon, France; 15 Metabolic Diseases Branch/NIDDK, National Institutes of Health, Bethesda, MD, USA; 16 Experimental Cardiology Unit, Department of Cardiovascular Medicine, University of Lausanne, Lausanne, Switzerland

**Keywords:** *GNB5*variants, *Gnb5*-null mouse models, IDDCA, cardiac conduction anomalies

## Abstract

**Background:**

Pathogenic variants of *GNB5* encoding the β_5_ subunit of the guanine nucleotide-binding protein cause IDDCA syndrome, an autosomal recessive neurodevelopmental disorder associated with cognitive disability and cardiac arrhythmia, particularly severe bradycardia.

**Methods:**

We used echocardiography and telemetric ECG recordings to investigate consequences of *Gnb5* loss in mouse.

**Results:**

We delineated a key role of *Gnb5* in heart sinus conduction and showed that *Gnb5*-inhibitory signalling is essential for parasympathetic control of heart rate (HR) and maintenance of the sympathovagal balance. *Gnb5^−/−^* mice were smaller and had a smaller heart than *Gnb5^+/+^* and *Gnb5^+/−^*, but exhibited better cardiac function. Lower autonomic nervous system modulation through diminished parasympathetic control and greater sympathetic regulation resulted in a higher baseline HR in *Gnb5^−/−^* mice. In contrast, *Gnb5^−/−^* mice exhibited profound bradycardia on treatment with carbachol, while sympathetic modulation of the cardiac stimulation was not altered. Concordantly, transcriptome study pinpointed altered expression of genes involved in cardiac muscle contractility in atria and ventricles of knocked-out mice. Homozygous *Gnb5* loss resulted in significantly higher frequencies of sinus arrhythmias. Moreover, we described 13 affected individuals, increasing the IDDCA cohort to 44 patients.

**Conclusions:**

Our data demonstrate that loss of negative regulation of the inhibitory G-protein signalling causes HR perturbations in *Gnb5^−^* ^/−^ mice, an effect mainly driven by impaired parasympathetic activity. We anticipate that unravelling the mechanism of *Gnb5* signalling in the autonomic control of the heart will pave the way for future drug screening.

## Introduction

Intellectual developmental disorder with cardiac arrhythmia (IDDCA, OMIM (Online Mendelian Inheritance in Man): #617173) is an autosomal recessive neurodevelopmental disorder with onset in early childhood. Inactivating and hypomorphic mutations in the β_5_ subunit of guanine nucleotide-binding protein (*GNB5*), respectively, cause severe and mild forms of the disorder.^1^ The former is associated with cognitive disability, poor or absent speech and/or severe cardiac arrhythmias. The moderate manifestation of the syndrome, also named language delay and ADHD/cognitive impairment with or without cardiac arrhythmia (LADCI) syndrome (OMIM: #617182), consists of mild intellectual impairment, language delay, attention deficit hyperactivity disorder (ADHD) and, in about half the cases, severe cardiac arrhythmia.^1 2^ Some patients with IDDCA also showed retinal dysfunction and nystagmus, epilepsy, hypotonia and gastrointestinal problems.^1–10^ The *GNB5* retinopathy is a unique combination of dual retinal signalling defects reminiscent of features of both bradyopsia and rod ON-bipolar dysfunction,^5^ while the IDDCA epilepsy is characterised by early seizure onset (~3 months of age) with focal seizures rapidly evolving into epileptic spasms and consequent generalised multifocal discharges.^4^

The heart rate (HR) is established by the sinoatrial node, the pacemaker of the cardiac muscle, and controlled by the autonomic nervous system. This autonomic nervous system consists of two anatomically and functionally distinct divisions: the sympathetic and the parasympathetic branches, whose functions are often antagonistic but work together to maintain balance. In the heart, the postganglionic fibres of the sympathetic trunk stimulate the β-adrenoreceptors, thereby increasing HR and force of contraction. The parasympathetic modulation of the heart is primarily mediated by acetylcholine release, which activates the M_2_-muscarinic receptors (M_2_R) present on cells innervated by parasympathetic postganglionic neurons, including sinoatrial node cells. The activation of M_2_R triggers G_i/o_ subfamily G-proteins, which turn on G-protein-gated inwardly rectifying K^+^ channels (GIRK) resulting in membrane hyperpolarisation and decrease in HR. Regulator of G-protein signalling (RGS) proteins negatively regulate the timing of this M_2_R-GIRK signalling. GNB5, a divergent member of the Gβ family, has the unique property of forming complexes with R7-RGS proteins.^11–16^ In particular, the GNB5-RGS6 complex is involved in cardiac GIRK deactivation kinetics. *Rgs6*-null mice manifested heart conduction anomalies and hypersensitivity to parasympathomimetics.^17^ Zebrafish model defective for *gnb5* gene correspondingly showed reduced heartbeat on reinforced parasympathetic stimulation, eye movement defects and altered swimming behaviour^1^ and cardiomyocytes differentiated from human induced pluripotent stem cells (iPSCs) edited to engineer the GNB5-Ser81Leu missense variant associated with LADCI showed a decrease in spontaneous activity on stimulation with carbachol compared with normal cells.^7^

Whereas homozygous *Gnb5*-null mice recapitulated many of the corresponding human disease phenotypes such as learning deficiencies, hyperactivity, impaired motor coordination and perturbed vision,^18–22^ a systematic cardiac evaluation has never been performed in a mammalian model. Here, we assessed heart electrophysiology of *Gnb5* mice models. We detected an increased frequency of sinus arrhythmias in *Gnb5^−/^* ^−^ animals, which have a smaller heart than wild-type and *Gnb5^+/−^*, but exhibited better cardiac function. *Gnb5^−/−^* mice also displayed enhanced parasympathetic sensitivity on stimulation with a cholinergic agonist. Consistent with this, transcriptome profiling of atria and ventricles revealed overexpression of genes involved in cardiac muscle contractility, along with reduced ventricular expression of genes required for development of pacemaker cells in *Gnb5^−/−^* mice. Finally, we expanded the number of ascertained IDDCA individuals and the *GNB5* mutational spectrum.

## Materials and methods

### Enrolment

All affected individuals and their family members were recruited in Pakistan (families R–V), France (family W), Egypt (families X and Y) and Iran (family Z) after signing a written informed consent according to ethical review boards policies. Clinical ascertainment included physical examinations, medical history interviews and specialised consultations by a certified neurologist and cardiologist as appropriate. Venous blood was collected in EDTA for DNA extraction according to standard procedures.

### Exome sequencing

Whole-exome sequencing of families R–V and Z was performed by Macrogen, Korea, as described in reference.^23^ Briefly, target enrichment was performed with 2 µg genomic DNA using the SureSelectXT Human All Exon Kit version 6 (Agilent Technologies, Santa Clara, California, USA) to generate barcoded whole-exome sequencing libraries. Libraries were sequenced on the HiSeqX platform (Illumina, San Diego, California, USA) with 50× coverage. Quality assessment of the sequence reads was performed by generating QC statistics with FastQC.^24^ The filtering strategy included screening for only exonic and donor/acceptor splicing variants. In accordance with the pedigree and phenotype, priority was given to variants rare or absent in public databases (1000 Genomes project, National Heart, Lung, and Blood Institute Exome Variant Server, Complete Genomics 69 and Exome Aggregation Consortium V.0.2).

Trio exome sequencing was performed in proband 37 (family W) and her parents using SeqCap EZ Medexome library preparation kit following manufacturer’s recommendations (Roche, Indiana, Indianapolis, USA). Libraries were sequenced on a NextSeq500 (Illumina) at a mean depth coverage of 73× with 93.3% of the target bases above 30×. Genomic alignment against the hg19/GRCh37 assembly and variant calling were, respectively, done with BWAMEM V.0.7.12 and GATK HaplotypeCaller V.3.4 (Broad Institute, Boston, Massachusetts, USA). Only highly confident variants were kept for analysis (total depth >9, alternative allele depth >4, no strand bias, mosaicism >10%). Rare variants were considered as having a frequency of <1% in GnomAD v2 dataset. Whole-exome sequencing of families X and Y was performed as described in Makrythanasis *et al*.^25^ Sanger sequencing in each family confirmed the segregation of *GNB5* variants with the phenotype.

### Mouse husbandry

The *Gnb5* mouse line was recovered from cryopreserved sperm using in vitro fertilisation. The knockout allele was engineered in a C57BL/6J inbred genetic background by heterozygous deletion of exon 3 in the germline, as previously described.^18 22^ Genetically modified animals were born and housed in the Animal Facility of the Centre for Integrative Genomics, under controlled temperature conditions and a 12-hour light–dark cycle with free access to water, normal chow and nest building material. Mouse genomic DNA was extracted from ear biopsies using the hot shot protocol^26^ and used for genotyping as described.^22^ To prevent the previously documented high mortality of *Gnb5^−^* ^/−^ pups at weaning,^18^ heterozygous breeding couples used to obtain knockout pups were given breeding food pellet enriched for proteins and vitamins (Kliba 3336, extrudate). Additionally, litters including *Gnb5* ^−/−^ pups were fed from 14 to 28 days of age, that is, starting 1 week before weaning, with powdered wet maintenance food (Kliba 3436) in a Petri dish placed directly onto the floor of the cages, an expedient that should provide easier access to the food for the pups.

### In vivo transthoracic ultrasound imaging protocol

Transthoracic echocardiography was performed using a 30 MHz probe and the Vevo 2100 Ultrasound machine (VisualSonics, Toronto, Ontario, Canada). A light anaesthesia was achieved with 1%–1.5% isoflurane, maintaining HR at 400–500 beats/min. The mice were placed in decubitus dorsal on a heated 37°C platform to maintain body temperature. The heart was imaged in the 2D mode in the parasternal long-axis view. From this view, an M-mode cursor was positioned perpendicular to the interventricular septum and the posterior wall of the left ventricle, at the level of the papillary muscles. Diastolic and systolic interventricular septa, left ventricular posterior wall thickness and left ventricular internal end-diastolic and end-systolic chamber dimensions were measured. Three separate M-mode images were measured and averaged. Left ventricular fractional shortening and ejection fraction were also calculated. Fractional shortening and ejection fraction were assessed from M-mode based on the percentage changes of left ventricular end-diastolic and end-systolic diameters and volumes, respectively. We used male mice at 9 weeks of age of three different genotypes (*Gnb5^+/+^*, *Gnb5^+/^* ^−^ and *Gnb5^−/−^*).

### In vivo electrocardiography measurements

For in vivo electrocardiography monitoring we have subcutaneously implanted biopotential telemetric transponders (ETA-F10, Data Sciences International) allowing continuous monitoring in conscious freely moving animals at 12 weeks of age. The negative electrode was implanted at the top of the right pectoral muscle, and the positive one was anchored at the level of the last left rib (at about 1 cm of the xiphoid appendix), thus leading to a normal lead II trace. Baseline ECG was recorded 10 days after device implantation, over a period of 86 hours. After 30 min of basal measurements, we injected mice with 0.9% saline solution (intraperitoneal NaCl, 10 mL/kg) as a vehicle control; next, the following compounds were administered one at a time: atropine (PubChem CID: 174174, intraperitoneal, 1 mg/kg), carbachol (PubChem CID: 5831, intraperitoneal 0.1 mg/kg) and in a subset of mice isoprenaline (PubChem CID: 3779, intraperitoneal 4 mg/kg) and atenolol (PubChem CID: 2249, intraperitoneal 2 mg/kg) as well, with a one night interval between each injection. The amounts of isoprenaline and atenolol were chosen after testing 10 doses ranging from 4 µg/kg to 4 mg/kg and 2 µg/kg to 2 mg/kg, respectively. While atropine and carbachol respectively inhibit and activate the parasympathetic system atenolol and isoprenaline respectively block and promote the sympathetic response.

At the end of the experiment, mice were sacrificed by CO_2_ inhalation; the heart was digitally imaged both within its thoracic position and after excision. Heart weight was recorded and tibia length was measured to normalise the heart weight to body size. Images were taken by a Leica DCF295 digital colour camera with 3M pixels mounted on a MZ6 stereomiscroscope (Leica, Switzerland). The length of the tibia was measured using a precision calliper after removal from the left leg (without patella nor articular cartilage).

Baseline ECG traces were analysed as follows: 10 min of recording were analysed using ECG-Auto software in shape recognition mode (EMKA Technology, France) every 30 min, during night and day phases. Mean values were reported for each analysed parameter. During pharmacological challenges, ECG recording was analysed continuously, with 10 min steps; thus, one mean of each parameter was calculated every 10 min. On ECG traces we analysed: (1) RR interval (measured at R peak, expressed in millisecond; (2) HR: heart beating rate, calculated as 60/(RR/1000), expressed in beats/min; (3) PR interval (interval between beginning of P wave and R peak, expressed in millisecond); (4) QT duration: duration of the QT complex; (5) QTc (corrected QT, calculated from QT/√(RR/100)^27^ (all expressed in millisecond) that allows correction of QT from HR variations).

Temperature and activity were also recorded. Activity was estimated by displacement of the telemetric device from the antennas of the recording plate. This measurement was only used as a qualitative index of mouse activity/movement. Temperature and activity values are mean values of 1-hour interval.

### Time-domain heart rate variation (HRV) analysis

Twelve-hour segments of day/night phases were selected from the baseline recording period and analysed separately for the HRV analysis. Specifically, R wave detection was performed and R-R interval time series were obtained. To ensure inclusion of sinus beats only, values not included between R–R intervals±2 SD were excluded as reported in Thireau *et al*.^28^ The analysed time-domain HRV parameters, computed using Kubios HRV Standard software V. 3.2.0, were: (1) mean R–R intervals (NN, in millisecond); (2) SD of all normal R–R intervals (SDNN, in millisecond); (3) square root of the mean square differences (RMSSD) between successive normal intervals (in millisecond); and (4) percentage of normal consecutive R–R intervals differing by >*x*ms (pNNx, in %, in this study x=6 ms).

### Arrhythmia assessment

Arrhythmias were identified based on ECG trace (RR interval) and counted. Observed arrhythmias were defined as follows: (1) escape atrial beat, with a P wave morphology different from that of the sinus P wave, classified as ‘long’ and ‘short’ according to the location of the P wave on the ECG trace (specifically, long was an escape beat whose duration was longer than two normal PP intervals, and short were those escape beats that lasted less than two normal PP intervals); (2) atrioventricular block defined on ECG by more than one P wave for one QRS complex; (3) premature beats; and (4) episodes of tachycardia followed by bradycardia, with HR oscillation between high and low values within a few seconds, independently of other type of arrhythmias.

### Statistical tests

Each parameter measured was reported as mean±SD. Standard t-test was used to assess differences between two groups. Analysis of variance tests, followed by Tukey post hoc tests, were also calculated and are displayed. A p value of <0.05 was considered significant, and stars on the plots represent the level of significance (*p≤0.05, **p≤0.01, ***p≤0.001, ****p≤0.0001; p>0.05 was considered not significant).

### Transcriptome profiling, data processing and differential expression analysis

A total of 72 RNA sequencing libraries were generated from two heart tissues, atria and ventricles, and three brain regions, cerebellum, hippocampus and cerebral cortex. Both the entire ventricles (left and right) or atria (left and right) were used for the sample processing; therefore, the atrial tissue represents a mixture of heart muscle cells, as well as sinoatrial and atrioventricular cells. The whole cerebellum, the hippocampi from each hemisphere of the brain and the whole cerebral cortex were dissected for RNA extraction. For this experiment, we created a cohort of adult (18 weeks) male mice including 6 *Gnb5^−/−^*, 6 *Gnb5^+/−^* and 6 *Gnb5^+/+^* animals. Brain transcriptome was only assessed in *Gnb5^−/−^* and *Gnb5^+/+^* animals. Tissue collection and processing procedures were designed to minimise biological and technical variation. Specifically, tissues were dissociated in QIAzol Lysis Reagent (Qiagen) using the gentleMACS Dissociator (Miltenyi Biotec). Cell suspension was used to obtain total RNA. Genomic DNA contamination was removed by digestion with RNase-free Deoxyribonuclease I (Qiagen). RNA concentration and purity were measured by ND-1000 spectrophotometer (Thermo Scientific, Wilmington, North Carolina, USA), and RNA integrity was verified by fragment analyser automated CE system (Advanced Analytical Technologies) according to manufacturer’s instructions. Libraries were then prepared with TruSeq Stranded RNA Library Prep Kit (Illumina) and sequenced on multiple lanes of an Illumina HiSeq4000 platform, generating an average of 50M single-end 125-cycle reads for each sample. Quality of sequence was assessed by FastQC V.0.11.4.^24^ Purity-filtered reads were adapters-trimmed and quality- trimmed with Cutadapt V.1.8.^29^ Reads matching to ribosomal RNA sequences were removed with fastq_screen V.0.11.1. Remaining reads were further filtered for low complexity with reaper V.15–065.^30^ Reads were then aligned against *Mus Musculus.GRCm38.92* genome using STAR V.2.5.3a.^31^ The number of read counts per gene locus was summarised with htseq-count V.0.9.1^32^ using *Mus Musculus.GRCm38.92* gene annotations. Quality of the RNA-seq data alignment was assessed using RSeQC V. 2.3.7.^33^ Reads were also aligned to the *Mus Musculus.GRCm38.92* transcriptome using STAR V. 2.5.3a,^31^ and the estimation of the isoforms abundance was computed using RSEM V.1.2.31.^34^ To assess differential expression between genotypes within each tissue, we compared *Gnb5^−/−^* and *Gnb5^+/−^* ± to wild-type specimens. Data analysis was performed with the R Bioconductor package DESeq2 V.1.14.1.^35^ Differentially expressed genes (DEGs) were identified at the Benjamini-Hochberg adjusted p<0.05 level, using Wald test under design ~genotype. For gene set enrichment analysis, no direction criterion on fold change was applied. Enriched Gene Ontology (GO) categories were identified using the enrichment analysis package in R/Bioconductor, clusterProfiler,^36^ considering only categories with at least 10 and maximum 500 annotated genes. Nominally significant enriched terms were retained for results interpretation.

### Western blotting analysis

Tissue lysates, including atria, ventricles, cerebral cortex, cerebellum and hippocampi, were prepared in RIPA buffer (Millipore) supplemented with protease inhibitors (Thermo Fisher Scientific). Tissues were homogenised using the gentleMACS Dissociator (Miltenyi Biotec). After SDS-polyacrylamide gel electrophoresis (SDS-PAGE) and transfer to nitrocellulose membrane, blots were incubated with anti-Gnb5, anti-Rgs7 (both a generous gift from Dr William F Simonds) and anti-Gnb3 (Cell Signalling Technology) antibodies, separately, and with an antiactin antibody (Sigma), used for loading control. Horseradish peroxidase-conjugated anti-rabbit antibody (Santa Cruz) and the ECL chemiluminescence system (Millipore) were used for detection.

## Results

### Clinical and molecular features of thirteen novel patients with IDDCA

**Figure 1:**
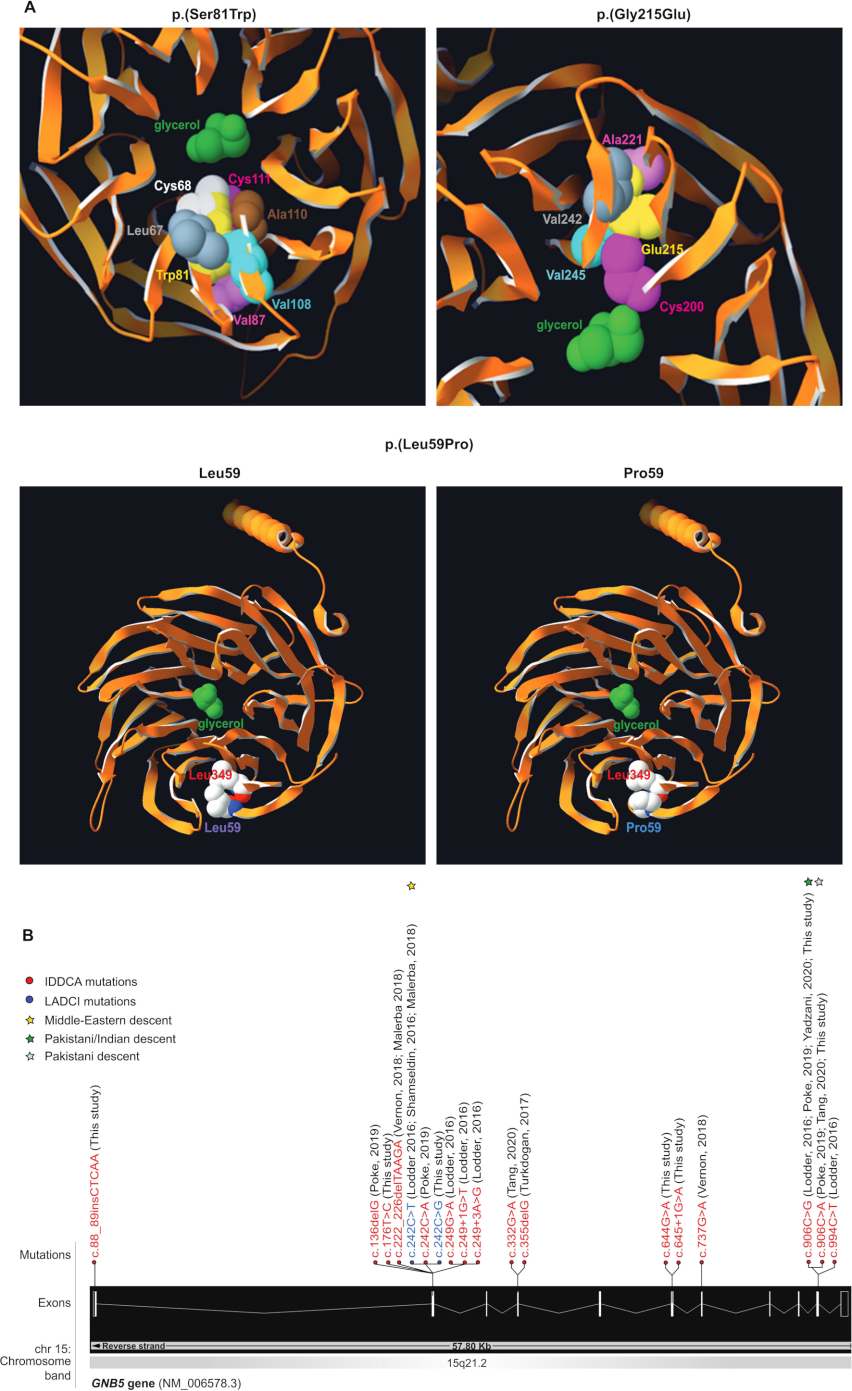
Variants modelling and IDDCA mutational spectrum. (A, top left) Top view of the Gnb5 (orange, PDB entry 2pbi) 3D protein model, showing the mutated Trp81 (yellow) and the glycerol molecule (green) in the centre of the pore. The rearrangements necessary to accommodate a tryptophan residue at position 81 will change the channel characteristics. The rotamer displayed here highlights clashes of Trp81 with Cys68 (white) and Cys111 (magenta). In additional rotamers, the bulky tryptophan sidechain will severely bump into Leu67 (grey), Val87 (pink), Val108 (cyan) and Ala110 (brown). (A, top right) As shown in this view of the beta propeller from above, the ‘wild-type’ Gly215 (not shown) lays in a beta-sheet, with on top Ala221 (pink), at the bottom Cys200 (magenta), and in front a beta-strand harbouring Val242 (grey) and Val245 (cyan). The presence of Glu215 cannot be tolerated, as it will encroach into one of the residues previously enumerated. Another rotamer shows clashes into Val245 (cyan). Overall, all rotamers may also force the sidechain of Cys200 (magenta) to reorient itself toward the internal part of the channel to provide space to accommodate glutamine at position 215 (yellow). In this position, the Cys200 sidechain will occupy the space dedicated to the glycerol (green), thus changing the properties of the channel. (A, bottom) The Leu59 (purple label, left panel) is positioned closely to the Leu349 residue just above (red label) in an antiparallel beta-sheet. This secondary structure will likely be broken in presence of a proline at that position (blue label, right panel), which in turn will disrupt the protein folding as the proline sidechain will collide into Leu349 (red label). (B) Distribution of the IDDCA published and novel variants along the schematically represented 11 exons of the human *GNB5* gene (transcript NM_006578.3; Ensembl (release 98, September 2019). The variants of IDDCA affected individuals are represented in red (LoF) and the missense LADCI variants in blue. The yellow star marks the variant of Middle Eastern descent, while green stars indicate the amber and ochre variants from the Indian subcontinent (dark green) and of Pakistani descent (light green), respectively. IDDCA, intellectual developmental disorder with cardiac arrhythmia; LADCI, language delay and ADHD/cognitive impairment; LoF, loss of function.

We identified 13 additional IDDCA cases (online supplemental figure S1, table S1 and table 1) through exome sequencing of nine consanguineous families and data aggregation of multiple laboratories and clinical centres via GeneMatcher^37 38^ or direct contacts. Consistent with previous reports,^1–10^ the carrier of a homozygous *GNB5* missense variant on Ser81 presented with LADCI, the mild form of IDDCA (family W), while the nine individuals with biallelic loss-of-function (LoF) alleles due to truncating or splicing mutations displayed phenotypes corresponding to the severe end of the disease spectrum (families R and S, U and V, and X and Z). The remaining three individuals (families T and Y) carrying novel homozygous missense variants on Gly215 (family T) and on Leu59 (family Y) similarly presented with the severe IDDCA phenotypical spectrum. Clinical features of affected individuals are summarised in table 1 and detailed in the online supplemental note and table S1. The three missense variants (c.644G>A, p.(Gly215Glu), family T, c. 242C>G, p.(Ser81Trp), family W and c.176T>C, p.(Leu59Pro), family Y; transcript NM_006578.3) were not reported before (figure 1). They are predicted by a majority of prediction tools to be likely damaging to protein function (online supplemental table S2) and are absent from GnomAD V.3.^39^ To assess possible impact, we modelled these three substitutions using the crystal structure of the GNB5-RGS9 complex^40^ and found that each variant could impact the potential binding properties of the GNB5 central pore (p.(Ser81Trp) and p.(Gly215Glu)) or the protein folding (p.(Leu59Pro)) (figure 1A). Specifically, Serine 81 is buried inside a β strand of the first WD40 repeat close to the central pore of the β propeller structure^1 2^ where a glycerol molecule is observed in pbi structure 2pbi (figure 1A, top left panel). Reminiscent of the Ser81Leu variant previously documented,^1^ a tryptophan at position 81 cannot be accommodated without disrupting the structure and potential binding properties of the pore. Our model suggests that the rearrangements necessary to settle such a bulky sidechain will change the channel characteristics. We have investigated the related rotamers, emphasising the steric hindrance-induced local rearrangements associated with the tryptophan replacement, whose perturbations were evaluated using the backbone-dependent rotamer library implemented in the Swiss-PdbViewer.^41^ Depending on the rotamer, tryptophan 81 will severely encroach with leucine 67, cysteine 68, valine 87, cysteine 111, valine 108 and/or alanine 110 (figure 1A, top left panel).

10.1136/jmedgenet-2020-107015.supp3 Supplementary data

10.1136/jmedgenet-2020-107015.supp4 Supplementary data

10.1136/jmedgenet-2020-107015.supp2 Supplementary data

**Table 1:** Overlapping clinical features of individuals with IDDCA and LADCI syndromes

	Individuals with phenotype/individuals (total n)	Phenotype severity (IDDCA)	Phenotype severity (LADCI)	Phenotype severity (intermediate)
Gender	23F, 21M	15F, 19M	7F, 2M	1F
Clinical examination				
Dysmorphic features	11/44 (25%)	10/34	–	1/1
Congenital malformations	4/44 (9%)	4/34 (heart)	–	–
Neurological manifestations				
Intellectual disability	37/44 (84%)	32/34	4/9	1/1
Speech delay	34/44 (77%)	27/34	6/9	1/1
Hypotonia	33/44 (75%)	29/34	3/9	1/1
Seizures	25/44 (57%)	25/34	–	–
Behavioural disorders	6/44 (14%)	3/34 (ASD)	3/9 (ADHD)	–
MRI anomalies	8/44 (18%)	8/34	–	–
Sleep disturbance	2/44 (5%)	2/34	–	–
Cardiac manifestations				
Sinus sick syndrome	27/44 (61%)	22/34	4/9	1/1
Pacemaker implantation	6/44 (14%)	4/34	1/9	1/1
Ophthalmological findings				
Nystagmus	26/44 (59%)	26/34	–	–
Strabismus	6/44 (14%)	5/34	–	1/1
Retinal disease	14/44 (32%)	14/34	–	–
Gastrointestinal problems				
Pathological gastric reflux	18/44 (41%)	18/34	–	–

Pedigree charts and variants of single individuals are detailed in the corresponding published reports^1–4 6 8–10 81^ and in online supplemental figure S1. Detailed phenotypical information of each affected individuals is reported in online supplemental table S1 and supplemental note.

ADHD, attention deficit hyperactivity disorder; ASD, autism spectrum disorder; F, female; IDDCA, Intellectual developmental disorder with cardiac arrhythmia; LADCI, language delay and ADHD/cognitive impairment; M, male.

Pedigree charts and variants of single individuals are detailed in the corresponding published reports^1–4 6 8–10 43^ and in (online supplemental figure S1). Detailed phenotypic information of each affected individuals is reported in (online supplemental table S1 and Supplemental Note). Abbreviations are as follows: M: male; F: female, ASD: Autism Spectrum Disorder, ADHD: Attention Deficit Hyperactivity Disorder.

Glycine 215 (figure 1A, top right panel) lies in a beta-sheet, between alanine 221 and cysteine 200. It faces a beta-strand harbouring valine 242 and valine 245. There is not enough space to accommodate the glutamic acid sidechain as it would encroach into one or more sidechains of the above-enumerated residues. All rotamers would also probably force the sidechain of cysteine 200 to reorient itself toward the interior of the channel to accommodate the glutamic acid 215 sidechain. This will infringe on the glycerol molecule space^40^ changing the channel characteristics. Additionally, the c.644G>A, p.(Gly215Glu) variant that affects the second to last nucleotide of exon 6 might alter the activity of the donor splice site as well as create cryptic exonic splicing enhancers or silencers according to the NNSplice, NetGene2 and Splicing Finder prediction tools (online supplemental table S2).

Leucine 59 (figure 1A, bottom left panel) is in close contact with leucine 349, and both residues, linked by a hydrogen bond, belong to an antiparallel beta-sheet. A proline at position 59 (figure 1A, bottom right panel) will likely destabilise (break) this hydrogen bond, thus representing a structural conundrum. Locally, the presence of a proline might disrupt the overall protein fold as there will not be enough space to accommodate this residue, its cyclic sidechain clashing into Leu349.

To date a total of 18 pathogenic *GNB5* variants and one homozygous deletion at 15q21.2 encompassing *GNB5* gene have been identified in 44 individuals with IDDCA (online supplemental ffigure 1S 1B and table S1). Suggestive of a founder effect, the eight affected individuals from three families (families E–G) carrying the Ser81Leu variant all originate from Arab countries (Morocco, Algeria and Saudi Arabia). Of note, the Greater Middle East Variome Project^43^ (http://igm.ucsd.edu/gme/) did not identify this variant within 2497 individuals. Similarly, the patients from six families (families D, L, P, R, S and V) harbouring the amber nonsense c.906C>G (Tyr302*) variant are from the Indian subcontinent (one from India and five from Pakistan). Another variant modifying the Tyr302 codon in an ochre codon (c.906C>A) was found in two additional Pakistani families (families N and U), suggesting again a possible founder effect. Of note, the same ochre variant was shown to be de novo on the paternal allele of the proband of the Chinese descent family O.^9^ The 34 IDDCA individuals present with the severe end of the disease spectrum, which is characterised by severe ID (32 out of 34) with poor or absent speech (27/34), early onset sinus node dysfunction (22/34) with 4/22 who had a pacemaker implanted, variable visual abnormalities (26/34), seizures (25/34), hypotonia (29/34) and gastrointestinal problems (18/34). Additionally, 9/34 individuals showed different types of dysmorphic features (table 1 and online supplemental table 1), and MRI evaluation revealed altered brain structure in 8/34 children, with four having thinner corpus callosum, two having long posterior and hypogenesis of corpus callosum, respectively, one cerebral atrophy and one cerebral and cerebellar cortical atrophy. Three individuals (individual 16, family H; individual 39, family X; and individual 44, Family Z) showed autistic features, and the other two displayed sleep disturbances (individual 27, family N, and individual 29, family P). All severely affected individuals carry either biallelic truncation mutations or biallelic missense variants that probably result in LoF. Nine patients displayed the milder LADCI syndrome and biallelic missense variants at position 81: 4/9 presented with mild ID with 6/9 showing language deficits; 4/9 were noted to have sinus node dysfunction (one of which with pacemaker implantation); and 3/9 were reported with impaired fine motor skills. Behaviorally, 3/9 patients exhibited ADHD. The remaining patient (family J) is compound heterozygous for the LoF p.Asp74Glufs52* and the Ser81Leu variants.^3^ She presented with an intermediate manifestation of the symptoms with mild ID accompanied by speech delay, hypotonia and sinus bradycardia (table 1). Like patient 21 from family I who carries the same LoF p.Asp74Glufs52* combined with a different missense, p.(Arg246Gln),^8^ she is affected by hearing loss (online supplemental table S1).

### *Gnb5* knockout mouse cohort

**Figure 2:**
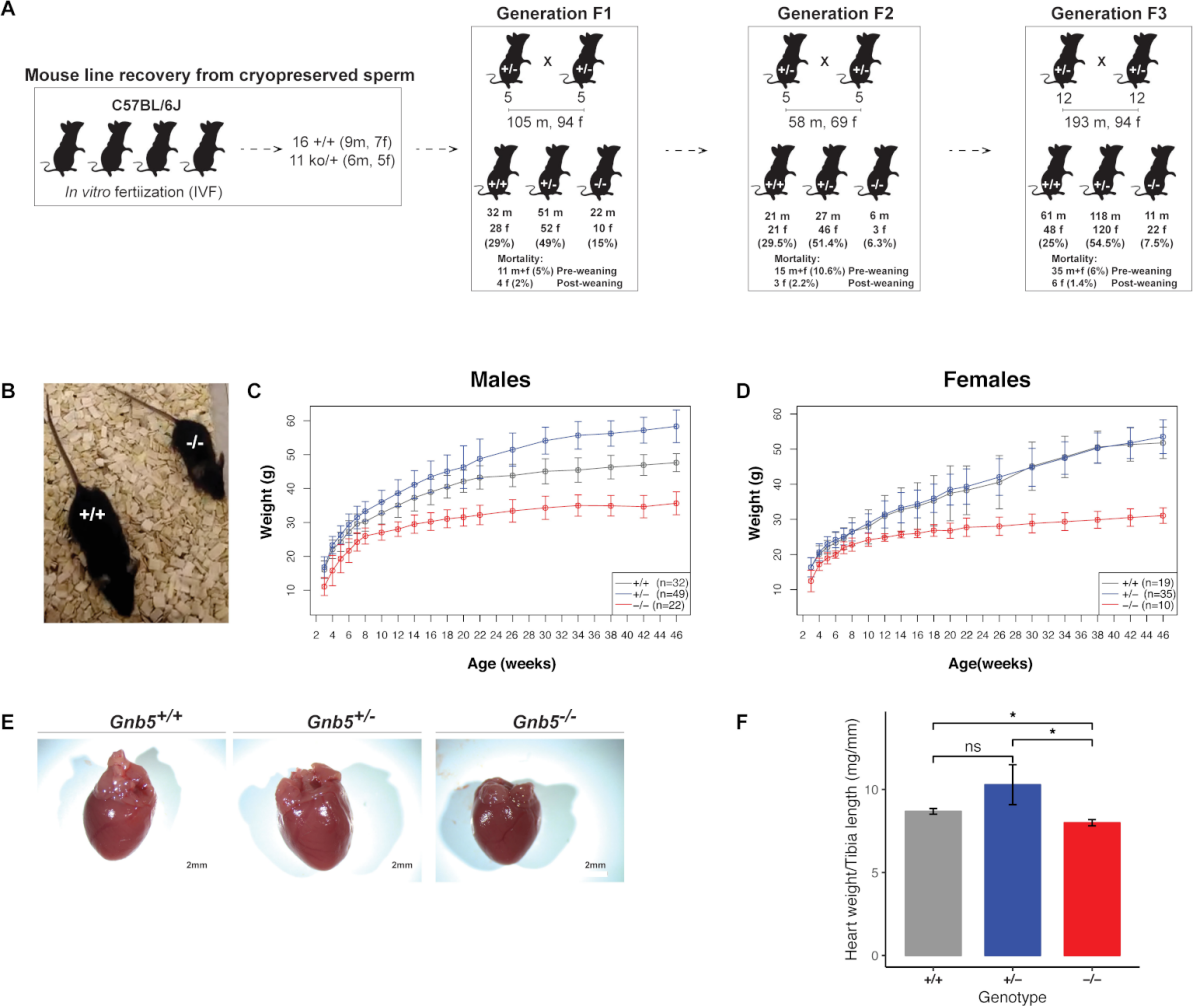
*Gnb5* mouse line features. (A) Mouse mating strategy and gender and genotype distribution over three successive generations. Preweaning and postweaning mortality is reported for each colony. (B) Size of *Gnb5^+/+^* and *Gnb5^−/^* ^−^ mice. (C, D) Body weights profile monitored from 3–46 weeks of age. All mice were weaned on week 3. Data are shown as mean±SD. (C, D) Panels separate body weights according to sex. *Gnb5^+/+^* is depicted in grey, *Gnb5^+/^* ^−^ in blue and *Gnb5^−/^* ^−^ in red. (E) At sacrifice, neither significant morphology difference nor thoracic position of the heart were observed among groups. (F) Bar plot showing that *Gnb5^−/^* ^−^ hearts (red, n=16) are smaller compared with the other genotypes (*Gnb5^+/^* ^−^, blue (n=8) and *Gnb5^+/+^,* grey (n=16)). Data are shown as mean of the ratio between heart weight and tibia length (used to normalise for animal size)±SEM. Asterisks on the plots represent the level of significance: *p≤0.05, **p≤0.01, ***p≤0.001, ****p≤0.0001; p>0.05 was considered not significant.

To model the cardiac manifestations occurring in IDDCA syndrome, we used the mouse model knockout for *Gnb5* (*Gnb5^–/^* ^–^), thus mimicking a complete LoF. Whereas ~66% of the pups carrying the homozygous null allele were previously reported to die prior to or at weaning,^18^ preweaning mortality was very low in our husbandry setting (see Materials and methods). We experienced only 5%, 11% and 6% of losses in the three cohorts we generated by mating heterozygous parents (figure 2A). We recorded two to eight breeding events during three generations of husbandry with an average litter size of 4–10 pups. The vast majority of preweaning lethality appears to be associated with *Gnb5^–/–^* as shown by the quasi-Mendelian distribution of genotypes (figure 2A). We longitudinally monitored the body weight of *Gnb5^+/+^* (wild type), *Gnb5^+/–^* and *Gnb5^–/–^* male and female mice from 3 to 46 weeks of age (figure 2C, D). We confirmed previous reports^18 22^ that showed that female and male knockout animals are smaller (figure 2B–D) and that heterozygote male mice are heavier (figure 2). Importantly, knockout mice had a smaller heart (figure 2E), even smaller than expected when the heart weight was normalised to the tibia length, a proxy for animal size (figure 2F). During animal handling, no obvious gender differences were observed regarding development, behaviour or other gross phenotypes.

### Ultrasound scans pinpointed increased cardiac function in *Gnb5* knockout mice

**Figure 3:**
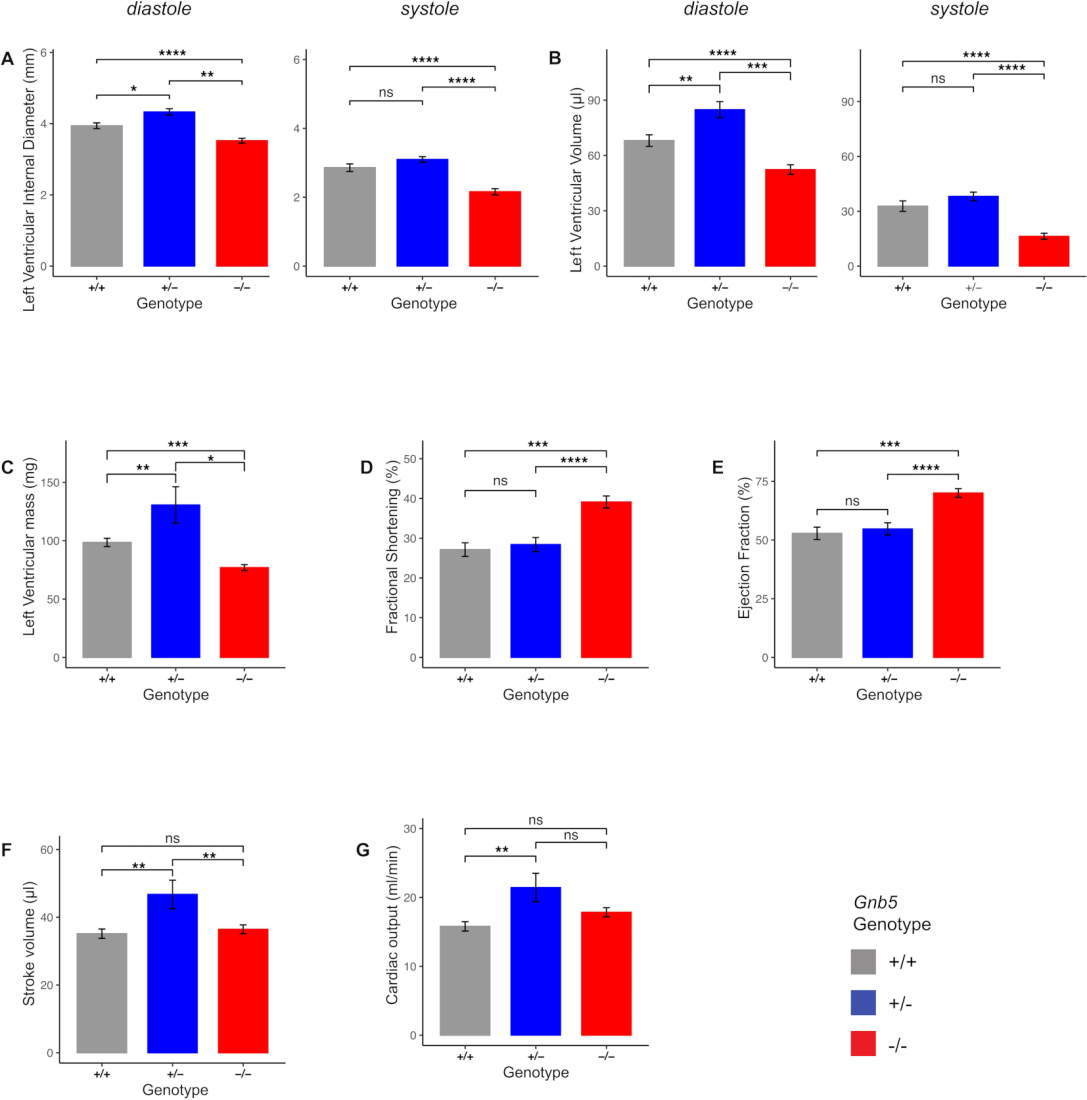
Morphological and functional parameters measured by ultrasound scan in *Gnb5^+/+^*, *Gnb5^+/^* ^−^ and *Gnb5^−/^* ^−^ male mice. (A) Left ventricular internal diameter at diastole (left) and systole (right). (B) Left ventricular volume in diastole (left) and systole (right). (C) Mass of the left ventricle. (D–G) Cardiac function expressed as fractional shortening (D), ejection fraction (E), cardiac output (F) and stroke volume (G). Parameters unchanged between the three genotypes are shown in online supplemental figure S2A, B.

To characterise the *Gnb5* knockout mouse line at cardiac level, we first performed ultrasound scans in baseline conditions. We used 16 *Gnb5^+/+^*, 8 *Gnb5^+/–^* and 16 *Gnb5^–/–^* male mice (9 wo (weeks of age)) and analysed heart morphology and function (see Materials and methods section). Echocardiography confirmed that *Gnb5^–/–^* animals had smaller hearts, as demonstrated by reduced ventricular chambers both in diastole and systole (figure 3A). Consequently, ventricular volume was also smaller (figure 3B). Estimated left ventricular weight was significantly lower in *Gnb5^–/–^* compared with *Gnb5^+/–^* and wild-type (figure 3C). Left posterior ventricular wall and interventricular septum thickness were not substantially modified (online supplemental figure S2A, B). Interestingly, *Gnb5^–/–^* mice demonstrated improved cardiac function, as judged by increased fractional shortening (figure 3D) and ejection fraction (figure 3E). However, stroke volume (figure 3F) and cardiac output (figure 3G) were similar to those measured in wild-type mice. Notably, and unexpectedly, *Gnb5^+/–^* have a bigger heart (figure 3A–C) compared with wild type, while their cardiac function remains unchanged (figure 3D–E). Therefore, the increased stroke volume (figure 3F) and cardiac output (figure 3G) reflected an increased volume of blood pumped by the ventricle.

Taken together, these results indicated that *Gnb5^–/–^* mouse hearts were smaller than that of the other two genotypes but compensated their smaller size by increased cardiac efficiency.

### Loss of functional *Gnb5* determines the onset of sinus arrhythmias

As cardiac arrhythmia in the form of bradycardia and ectopic beats is one of the core symptoms in IDDCA, we examined HRVs in *Gnb5* mouse models with in vivo ECG monitoring. ECG was performed at 12 weeks on the same male mice used for echocardiography. Baseline ECG parameters were not different among *Gnb5^+/+^*, *Gnb5^+/–^* and *Gnb5^–/–^* animals, except for HR in knocked-out animals that showed a trend toward higher values (online supplemental figure S2C, minimum and maximum HR values registered in wild-type were 313 and 741 beats/min, while lowest and greatest HR values in knockout were 349 and 744 beats/min, significance varying between p=1.28E-04 and p=0.9971, over 36 daylight time points).

**Figure 4:**
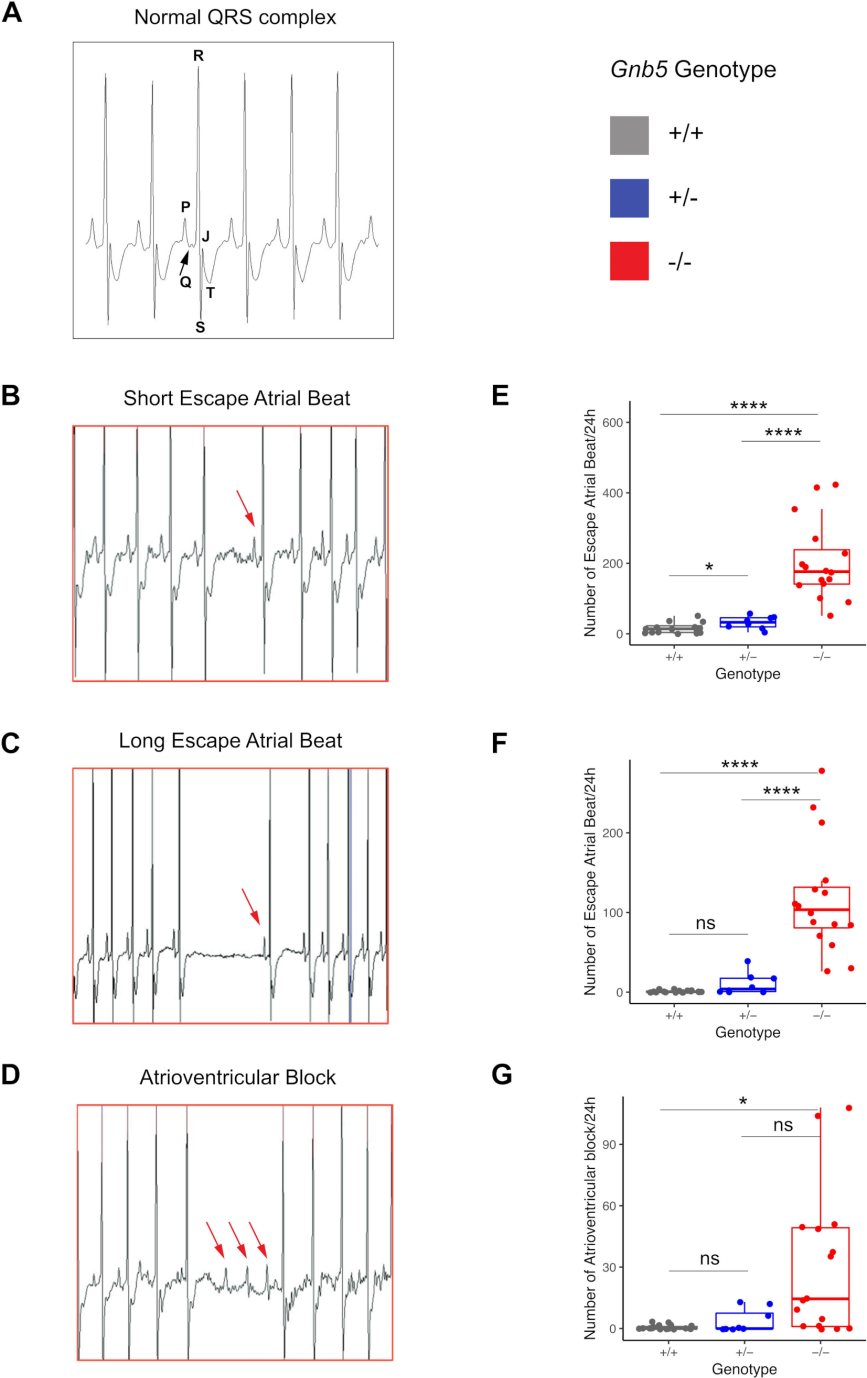
Cardiac arrhythmias recorded in *Gnb5* mouse line. (A) Normal ECG trace recorded in wild-type male mice with the main spikes specified as used in the text. (B, C) *Gnb5^−/−^* male mice ECG traces showing escape atrial beats classified as short (B) and long (C) and characterised by the occurrence of a late P-wave (red arrow). (D) *Gnb5^−/^* ^−^ male mice ECG trace demonstrating atrioventricular blocks, with more than one P-wave per QRS complex (consecutive red arrows). (E–G) Respective box plots indicating the number of arrhythmias, that is, the number of short (E) and long (F) escape atrial beats and atrioventricular blocks (G) per 24 hours. ns, not significant.

Close inspection of baseline ECG over a 24-hour window allowed the quantification and characterisation of small changes in the intervals between successive heartbeats (RR interval) corresponding to cardiac arrhythmias (Materials and methods). The 24-hour ECGs unearthed a significant increase in arrhythmic events in *Gnb5^–/–^* mice compared with heterozygous and wild-type littermates. We counted on average 53 short atrial escape beats in *Gnb5^+/+^* and 204 in *Gnb5^–/–^* animals over 24 hours (figure 4B–E, p=4.936e-06). Long atrial escape beats (figure 4C–F; 1 vs 117 events, p=7.792e-06) and atrioventricular blocks (figure 4D–G; 0.6 vs 30 events, p=0.04078) were similarly observed significantly more frequently in knockout animals. Few episodes of tachycardia followed by bradycardia and premature beats were also observed in homozygous knockouts.

These results demonstrated that *Gnb5^–/–^* mice have a defect at the level of sinus node as well as cardiac conduction anomalies linked to the atrioventricular node. Of note, we did not assess for other anomalies.

### *Gnb5*-deficient mice exhibit higher cholinergic sensitivity and normal sympathetic activity

Human homozygote carriers of *GNB5* pathogenic variants show severe bradycardia at rest with a maximal HR unchanged during exercise. Zebrafish and human cell modelling supported predominantly parasympathetic modulation in the aetiology of HR disturbances in IDDCA individuals.^1 7^ To better assess the possible involvement of the autonomic innervation, we used an in vivo mammalian model system whose physiology is closer to humans. We used ECG telemetry to monitor HR and observed higher HR in baseline conditions (online supplemental figure S2C) possibly reflecting a higher rate of activity of the *Gnb5^–/–^* mice, as measured qualitatively here and reported previously^22^ (online supplemental figure S2D, E), or alternatively differences in the HR regulation.

**Figure 5:**
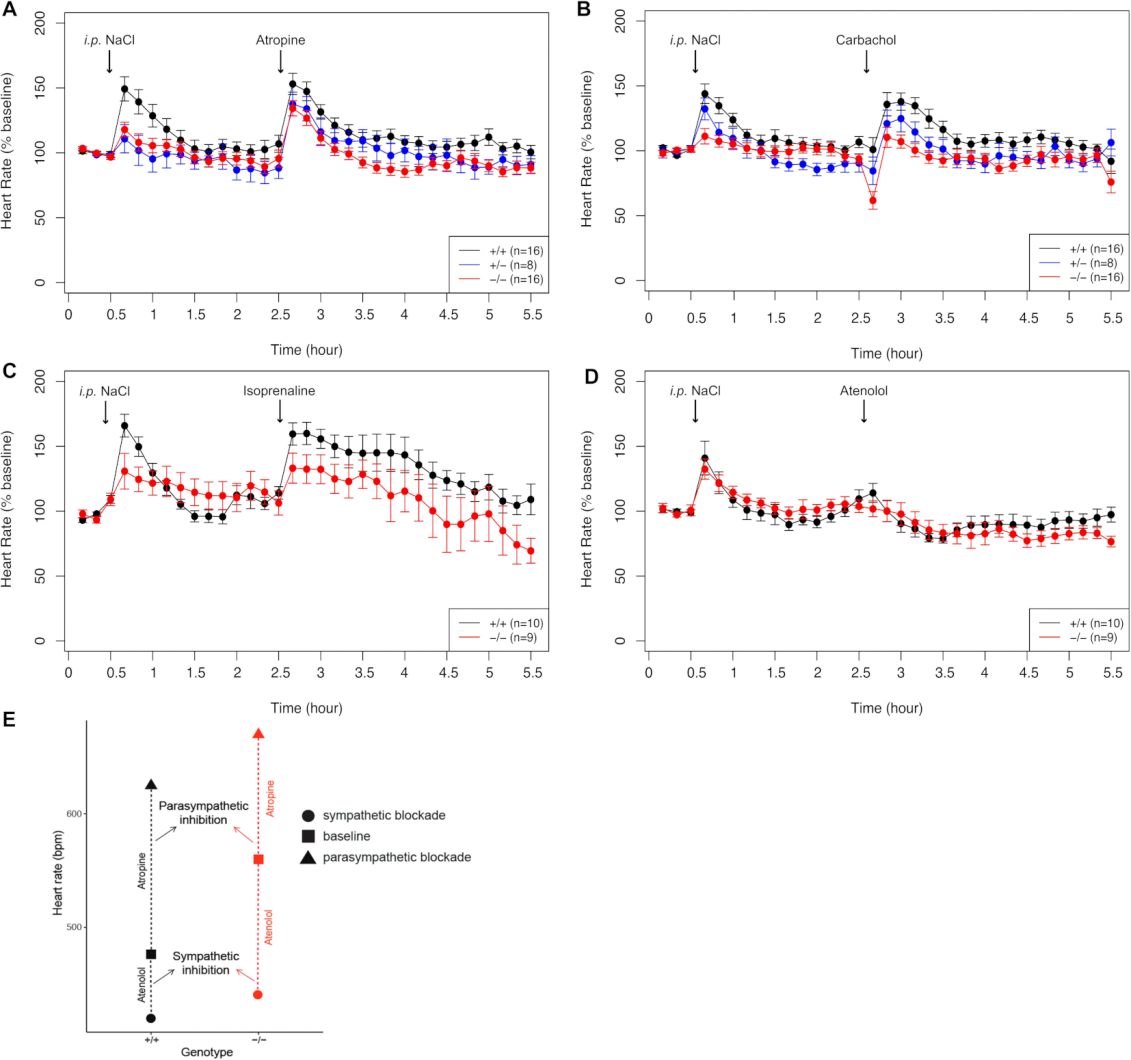
Pharmacological administration of compounds mimicking parasympathetic and sympathetic stimulation. (A) HR monitoring after injection of atropin (intraperitoneal 1 mg/kg). (B) Bradycardia measured in response to carbachol (intraperitoneal 0.1 m/kg). (C) HR variation in response to atenolol (intraperitoneal 2 mg/kg). (D) Increased HR after isoprenaline administration (intraperitoneal 4 mg/kg). Data points are expressed as percentage of the baseline values. (E) Smaller parasympathetic and bigger sympathetic blockade in *Gnb5^−/−^* mice (red), compared with wild-type littermates (black), indicative of lower parasympathetic and higher sympathetic tones in basal conditions. HR, heart rate.

Parasympathetic blockade with atropine (1 mg/kg) had a positive chronotropic effect; that is, HR increased (HR *Gnb5^+/+^* =690 beats/min±68, HR *Gnb5^+/–^* =700 beats/min±34, HR *Gnb5^–/–^* =758 beats/min±28 (p_+/+ vs. +/–_=0.63, p_+/+ vs –/–_=2.62E-03, p_+/– vs –/–_=2.38E-03)) (online supplemental figure S3A). In contrast, carbachol administration (0.1 mg/kg) triggered a rapid decrease of the HR in the three groups, with a significant effect in *Gnb5^–/–^* mice, whose HR dropped until 335 beats/min (HR *Gnb5^+/+^* =448 beats/min±147, HR *Gnb5^+/–^* =415 beats/min±157, HR *Gnb5^–/–^* =335 beats/min±141 (p_+/+ vs –/–_=3E-02; online supplemental figure S3B). HR quickly recovered in all genotypes. The duration of the bradycardia was similar in the three groups (~1 hour). Moreover, since the baseline HR of knockout animals was higher, when expressed in relation to the basal values, the effect of atropine was not different in the three groups of mice (p_+/+ vs +/–_=0.22, p_+/+ vs –/–_=0.07, p_+/– vs –/–_=0.77; figure 5A), while the carbachol-induced bradycardia was more severe in *Gnb5^–/–^* (p=1.79E-03, figure 5B).

To mimic the sympathetic response and investigate a possible role of the β-adrenergic response in the heart rhythm perturbations of IDDCA syndrome, we challenged *Gnb5^+/+^* and *Gnb5^–/–^* animals with either isoprenaline or atenolol. Injection of the sympathetic agonist isoprenaline (4 mg/kg) resulted in a prolonged (~1 hour) increase of HR, with values comparable in both genotypes (p=0.41, online supplemental figure S3C). HR slowly decreased to baseline; this reduction reached lower than baseline values in *Gnb5^–/–^* mice (online supplemental figure S3C). However, when expressed in percentage of the baseline, the tachycardia seemed stronger in wild-type (p=0.09, figure 5C).

The sympathetic antagonist atenolol (2 mg/kg) induced a similar decrease of HR in both groups (p=0.73, p=0.22 when compared with baseline (online supplemental figure S3D and figure 5D).

These results indicate that the *Gnb5^–/–^* mice bradycardia results from enhanced parasympathetic (cholinergic) stimulation/reflex. Our data also suggest an increased sympathetic activation when animals are under stress, that is, when they are challenged by the injection of drugs.

In contrast to individuals affected with IDDCA, *Gnb5^–/–^* mice showed a higher HR in baseline conditions (online supplemental figure S2C and S3A-D, first three data points). We hypothesised that such discrepancy could be linked to different autonomic nervous system tone between human and mouse.^42^ We therefore analysed the response to the administration of drugs to determine which of the parasympathetic and sympathetic system mostly influences baseline HR. Atropine-mediated parasympathetic inhibition induced an increase in HR with a variation from the baseline, which was smaller in knockout animals than in wild types (figure 5E), pinpointing that the parasympathetic tone is lower in *Gnb5^–/–^* mice. Conversely, the sympathetic blockade by atenolol induced a greater reduction of the HR in *Gnb5^–/–^* than control littermates (figure 5E) evocative of the greater sympathetic tone observed in basal conditions. Of note, the control responses mediated by NaCl injection also showed a difference in HR attributable to increased parasympathetic/sympathetic balance; the HR increase, due to the stress caused by the injection and animal handling, was less pronounced in *Gnb5^–/–^* mice. Our results suggest that higher basal HR in *Gnb5^–/–^* mice could be due to lower parasympathetic tone and higher sympathetic tone.

To further assess the functioning of cardiac autonomic regulation, we performed time domain analysis of HRV in *Gnb5^+/+^* and *Gnb5^–/–^* mice. We measured NN, SDNN, RMSSD and pNN6 during light (day) and dark (night) phases in each genotype. Whereas all parameters differ in the wild-types, the knockouts showed no significant HRV differences between light and dark phases (table 2) confirming that differences in RR intervals (NN), and hence in HR, were greater in *Gnb5^+/+^* (p_NN_=1.25E-08, table 2) than in *Gnb5^–/–^* mice (p_NN_=0.72, table 2). This suggests abnormal autonomic regulation, in particular via the parasympathetic system in the *Gnb5^*–*/*–*^* mouse. Concordantly, RMSSD, which reflects short-term variations in HR, and pNN6, both measures of the parasympathetic nervous system regulation, were significantly different between night and day in *Gnb5^+/+^* mice (p_RMSSD_=4.38E-05, p_pNN6_=9.71E-06; table 2), while they were almost unchanged in *Gnb5^*–*/*–*^* mice (p_RMSSD_=0.65, p_pNN6_=0.9, table 2). Similarly, SDNN that indicates the total autonomic variability, fluctuated in *Gnb5^+/+^* mice (p_SDNN_=1.16E-02, table 2) but not in *Gnb5^*–*/*–*^* mice (p_SDNN_=0.61, table 2) at night.

**Table 2:** Results of HRV analysis

Genotype	*Gnb5^+/+^*							*Gnb5^−/−^*							*Gnb5^+/+^* vs *Gnb5^−/−^*	
Night/day	Night			Day			P value	Night			Day			P value	P value	P value
	Mean	SD	SEM	Mean	SD	SEM	Night versus day	Mean	SD	SEM	Mean	SD	SEM	Night versus day	Night	Day
Mean RR (NN)	112.27	10.32	2.66	136.81	13.25	3.31	1.25E-08	110.00	7.45	1.92	111.63	24.18	6.04	0.72	0.21	3.45E-03
SDNN	23.47	3.44	0.89	26.56	5.02	1.25	0.012	24.05	4.16	1.11	23.17	3.31	0.83	0.61	0.94	4.76E-02
RMSSD	4.77	1.74	0.45	6.54	2.10	0.53	4.38E-05	5.05	2.49	0.64	4.69	1.05	0.26	0.65	0.86	9.07E-03
pNN6	13.81	8.96	2.31	24.13	10.49	2.62	9.71E-06	13.33	7.98	2.06	13.21	5.00	1.25	0.90	0.88	7.34E-04

HRV parameters measured in time-domain HRV analysis over 12-hour nocturnal and 12-hour diurnal intervals. Values are reported as mean; SD and SEM were calculated and reported. Standard t-test was used to assess differences between Gnb5^+/+^ and Gnb5^−/−^ groups, as well as for differences between day and night. Description of the parameters used can be found in the Materials and Methods section.

HRV, heart rate variation; NN, mean R–R interval; RMSSD, square root of the mean square difference; SDNN, SD of all normal R–R intervals.

All parameters were significantly reduced during the light phase when comparing *Gnb5^+/+^* and *Gnb5^*–*/*–*^* mice (p_NN_=3.45E-03, p_SDNN_=4.76E-02, p_RMSSD_=9.07E-03, p_pNN6_=7.34E-04; table 2).

Taken together, these results demonstrated an impaired autonomic regulation in *Gnb5^*–*/*–*^* mice, in particular a lower modulation of the parasympathetic activity.

### Transcriptome analysis of *Gnb5* mice

**Figure 6:**
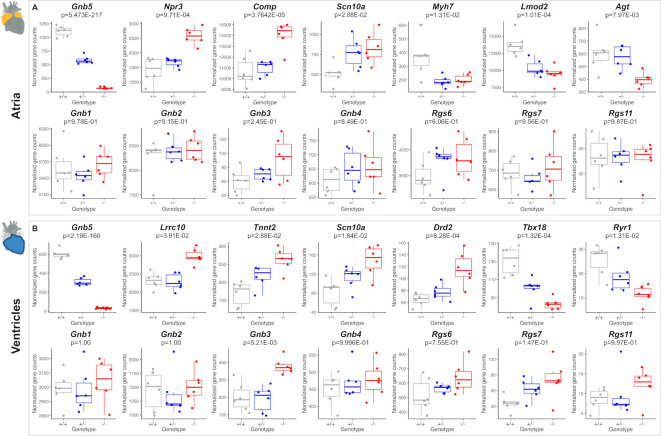
Comparison of transcriptome profiles of atria and ventricles in *Gnb5^+/+^*, *Gnb5^+/^* and *Gnb5^−/−^* male mice. (A) Atrial expression profiles of *Gnb5* (top, left) and other DEGs (top Lane); *Gnb* and *Rgs* transcripts quantification in atria (bottom lane). (B) Ventricular expression profiles of *Gnb5* gene (top, left) and other DEGs (top lane); *Gnb* and *Rgs* transcripts quantification in ventricles (bottom). DEG, differentially expressed gene.

To explore the transcriptional consequences of *Gnb5* loss in the heart, we profiled the transcriptomes of atria and ventricles of *Gnb5^*–*/*–*^*, *Gnb5^+/*–*^* and wild-type male mice at 18 weeks of age using RNA-sequencing (RNA-seq) (online supplemental table S3). RNA-seq libraries were sequenced to a median depth of ~50 000 000 single-end reads per samples. Samples clustering (using Poisson model^44^) to define the global relationship among all samples, showed a very clear separation of atria and ventricles (online supplemental figure S4A) as well as of hippocampi, cerebellum and cerebral cortex (online supplemental figure S4B). Transcripts quantification confirmed that *Gnb5*, the orthologue of *GNB5*, is expressed in the brain (online supplemental figure S5) and cardiac tissues (atria and ventricles; figure 6A, B, top) of adult mice and that its expression levels correlated with gene dosage. We identified 98 significantly DEGs in atria (online supplemental table S4) and 63 in ventricles (online supplemental table S5) applying a false discovery rate method for multiple testing with a 5% threshold. Consistent with the phenotype described in *Gnb5^*–*/*–*^* mice, we found altered expression of genes involved in cardiac muscle contractility, HR regulation and cardiac conduction. Among the upregulated genes falling into these categories in atria were *Npr3* (p=9.71E-04), *Comp* (p=3.7E-05) and *Scn10a* (p=2.88E-02), while downregulated transcripts were *Myh7* (p=1.31E-02), *Lmod2* (p=1.04E-04) and *Agt* (p=7.97E-03) (figure 6A, top). Profiling of the ventricles demonstrated significant upregulation of *Lrrc10* (p=3.91E-02), *Tnnt2* (cardiac troponin, p=2.88E-02), *Scn10a* (p=1.84E-02) and *Drd2* (p=8.28E-04) (figure 6B, top), and reduced expression of *Tbx18* (p=1.32E-04). Notably, the expression level of *TNNT2* gene was not modified in GNB5-Ser81Leu hiPSC.^7^ Correspondingly, nominally enriched gene sets were associated with development of the cardiac conduction system (GO:0003161, online supplemental table S6, S7), regulation of HR (GO:0002027, online supplemental table S6, S7) and cardiac muscle contraction (GO:0060047, GO:0060048; online supplemental table S6, S7). *MYH7* and *TNNT2* encode sarcomeric proteins, essential for contraction and relaxation of the heart muscle, and mutations in these genes have been linked to cardiomyopathy.^45^

*Scn10a* encodes the voltage-gated sodium channel Na_V_1.8. Its human orthologue was associated with HR regulation and genome-wide association studies suggested it as a modulator of PR interval duration, that is, atrial conduction time.^46^ *Lrrc10* and *Lmod2* are associated with dilated cardiomyopathy in both human and mice^47–50^; while *Lrrc10* functions in the sarcomeric Z-disc and the T-tubule components, involved in muscle contraction,^47^ loss of functional *Lmod2* has been linked to short thin filaments and reduction in maximum calcium-activated force production.^48^ Rodent ventricular cardiomyocytes are converted into spontaneous firing cells (eg, sinoatrial-node pacemaker cells) by expression of the transcription factor *Tbx18*,^51^ whereas a polymorphism in *DRD2* has been associated with motor learning and HR.^52^ *RYR1* has a dominant role in muscles contractility, even though it is more prominently associated with skeletal muscle.^53^ Common variants in *RYR1* are associated with left ventricular hypertrophy.^54^

Given that (1) *Gnb5* has four paralogous genes, with ~50% sequence identity^55 56^; (2) the respective encoded proteins may have redundant function; (3) the propagation of the Gnb5-mediated signal is controlled by RGS^15 16 57^; and (4) Gnb5 and Rgs (ie, Rgs6 and Rgs7) are coexpressed both at RNA (online supplemental figure S6A, B) and protein levels,^17^ we investigated the expression patterns of these two families of genes in our transcriptome profiles to investigate possible compensatory mechanisms. We found significant upregulation of *Gnb3* in ventricles (figure 6B, bottom, p=5.21E-03) and a trend toward increased expression level in atria (figure 6A, bottom, p=0.24). The genes encoding the other Gβ subunits, named *Gnb1*, *Gnb2* and *Gnb4*, did not change expression (figure 6A, B, bottom). In ventricles, the three R7-Rgs expressed in heart, *Rgs6*, *Rgs7* and *Rgs11*, showed a trend towards increased expression when *Gnb5* was knocked out (figure 6B, bottom). Only *Rgs6* showed a similar trend in atria (figure 6A, bottom). *Gnb5^+/−^* mice showed no or very subtle changes of their transcriptome compared with controls; we detected only six DEGs in atria (online supplemental table S8) and one in ventricles (online supplemental table S9) beside the engineered *Gnb5*. Profiling Gnb5 protein expression revealed detectable levels in the heart of wild-type mice, along with abundant presence in three brain regions (cortex, cerebellum and hippocampi), while immunoblotting of *Gnb5^−/−^* tissues verified the complete absence of the protein (online supplemental figure S6C). Notably, we detected no compensatory changes in either Gnb3 or Rgs7 levels; the two proteins were undetectable in *Gnb5^−/−^* atria and ventricles (online supplemental figure S6C). This suggests that Gnb3 protein stability may be decreased and further demonstrates that the physical association with Gnb5 is critical for the stability of the Gnb5/R7-Rgs complex, as previously described for Rgs6.^17^ Gnb5 and Rgs7 proteins were enriched in atria compared with ventricles in wild-type mice (online supplemental figure S6C).

We further investigated transcriptome signatures in three brain regions relevant to the IDDCA pathology: the cortex because of its role in higher cognitive function, the hippocampus as it participates in the formation of long-term and spatial memories and the cerebellum for its motor control and language processing. Cerebellar and hippocampal anomalies were documented in *Gnb5* knockout pups.^22^ Overall, we observed 206 DEGs in cerebellum, 105 in hippocampus and 48 in cerebral cortex (at false discovery rate (FDR) 5%) (online supplemental table S10-S12). These genes resulted in over-representation of gene ontology (GO) terms involved in regulation of excitatory postsynaptic membrane potential (GO:0060079), regulation of neurotransmitters secretion (GO:0046928), learning (GO:0007612) and synapse organisation (GO:0050808), regulation of inositol 3-phosphate (GO:0014065), regulation of cAMP-mediated signalling (GO:0043951) (in cerebellum, online supplemental table S13); visual perception (GO:0007601), phototransduction (GO:007602), guanylate cyclase activity (GO:0031282) and regulator of signalling receptors (GO:2000272) (in hippocampus, online supplemental table S14); and sensory perception of light stimuli (GO:0050953) and lens development (GO:0002088) (in cortex, online supplemental table S15).

Quantitative RT-PCR and microarray data previously indicated altered expression levels of genes implicated in neuronal development and function, such as down-regulation of *Grid2* (glutamate ionotropic receptor, delta 2) and upregulation of *Synpo* (synaptopodin),^22^ as well as increased expression of *Guca1a* and *Guca1b,* calcium-binding protein activating photoreceptor guanylate cyclases. *Guca1a* (p_hippocampus_=1.81E-03) and *Guca1b* (p_cerebellum_=1.56E-302, p_hippocampus_=1.9589E-196, p_cortex_=2.209E-178) were DEGs in the brain of *Gnb5* knockout mice; they showed significant upregulation (online supplemental figure S5). *Synpo* showed a trend of increased expression in the hippocampus (online supplemental figure S5B, p=0.55).

A subset of additional DEGs included *Grin2a* (p=1.044E-06), *Snca* (p=7.96E-03), *Gnai2* (p=7.79E-03) and *Kcnj2* (p=0.04), in the cerebellum (online supplemental figure S5A), *Hes5* (p=0.04) and *Sox6* (p=0.02) in the hippocampus (online supplemental figure S5B) and *Ntrk3* (p=9.69E-04) and *Cacna2d4* (p=0.03) in the cortex (online supplemental figure S5C). The glutamate-gated ion channel Grin2a protein plays important roles in long-term potentiation and in efficient synaptic transmission. Disruption of this gene is associated with focal epilepsy and speech disorder with or without cognitive disability.^58^ *Hes5* and *Sox6* genes, respectively, are a transcriptional repressor and activator, required for the regulation of transition timing of neurogenesis and gliogenesis in mammalian neocortical development^59^ and in the normal development of the central nervous system.^60^ *Cacna2d4* encodes for a calcium channel whose mutations are associated with retinal dysfunction in human.^61^ The expression of transcripts encoding for different Gβ subunits and R7-RGS genes remained unchanged (online supplemental figure S5 A-C, bottom), suggesting that the brain could be less proficient than the heart in compensating dysregulated pathways. Rgs7 protein level was greatly reduced in *Gnb5^−/−^* brain, further emphasising the Gnb5/R7-Rgs codependence (online supplemental figure S6C).

## Discussion

The advent of high-throughput sequencing allowed identification of the cause of hundreds of Mendelian diseases,^62^ in particular those involving intellectual disability.^63^ For example, data aggregation of exome sequencing uncovered a link between variants in genes encoding the Gβ subunits of the heterotrimeric G-proteins with a group of neuropsychiatric conditions with cardiac manifestations and ophthalmic pathologies.^1–4 6 8–10 64–79^ Mutations in *GNB5*, encoding the divergent Gβ_5_ subunit of the guanine–nucleotide binding protein family, were recently shown to be causative of the autosomal recessive IDDCA syndrome. Differently from Gβ_1–4_, Gβ_5_ forms irreversible dimer with the G-protein γ-like domain^80^ present in the R7 regulator group of G-protein signalling proteins (R7 RGS). Dimerisation with Gβ_5_ is absolutely required for the stability of the R7 RGS proteins^16^.

Genotype–phenotype correlation showed that carriers of truncating *GNB5* variants present with the severe form of this syndrome, while missense alleles are associated with milder phenotypical manifestations. Recently, an individual carrying a homozygous deletion spanning three genes, *BCL2L*, *GNB5* and *MYO5C*, was reported to display a phenotype which overlaps significantly with the IDDCA manifestations,^81^ further confirming that this phenotype is associated with loss of function of *GNB5*. In this work, we increased the number of reported individuals with IDDCA and associated variants, through identification of nine additional families and five novel causative variants. The core symptoms of IDDCA include cognitive disability, epilepsy, retinopathy and cardiac arrhythmia.^1–10^ This is a life-long condition with a risk of sudden death. Six of the seven deceased IDDCA individuals are suspected to have died from a sudden cardiac arrest secondary to an arrhythmia. Although the mutational spectrum of this syndrome has already been partly defined, the overall molecular mechanism by which perturbations of *GNB5* translate into IDDCA phenotypical manifestations remains unclear. Modelling in zebrafish and cardiomyocytes differentiated from human iPSCs provided some initial answers^1 7^; however, we thought that the mouse would be a more appropriate tool to investigate consequences of this human syndromic neurodevelopmental condition. As little was known about possible cardiac conduction anomalies in mice, we characterised both the cardiac phenotype and molecular outcome of knocking-out *Gnb5. Gnb5^−/−^* animals were smaller and had smaller ventricles, and therefore the chambers would contain less blood as a consequence. However, they exhibited an increase in fractional shortening and ejection fraction, a sign of compensatory cardiac function. The quantity of blood ejected by the ventricles, as defined by the cardiac output and stroke volume, remained unchanged between *Gnb5^−/−^* and *Gnb5^+/+^* mice, indicating that *Gnb5^−/−^* heart has adapted to ensure that it efficiently meets the body’s demands for perfusion.

ECG measurements showed that, conversely to IDDCA affected individuals who present with severe bradycardia at rest, *Gnb5^−/−^* mice have higher HRs in basal condition, especially during the day, the mouse sleeping phase. Zebrafish knocked-out for all *gnb5a* and *gnb5b* copies present in the fish genome, showed a similar trend of increased basal HR.^1^ It is plausible that these differences are linked to a lower parasympathetic tone in fish and rodents compared with humans. Of note, we showed that the parasympathetic and sympathetic tones of *Gnb5* ^−/−^ mice were, respectively, lower and higher than those of controls (figure 5E), thus possibly influencing the basal HR. Whereas we cannot conclude that this higher basal HR is exclusively due to changes in HR regulation as we and others observed an increase in activity of *Gnb5* ^−/−^ mice^21 22^ (online supplemental figure S2D), the zebrafish model showed no sign of hyperactivity.^1^ In normal conditions, HR is balanced by the synergic interactions between the sympathetic and parasympathetic nervous systems. Our HRV analysis, aimed at assessing such sympathovagal balance, confirmed a reduced modulation of the parasympathetic stimulation in the heart of *Gnb5* ^−/−^ mice (table 2).

ECG recordings additionally revealed a high number of arrhythmias in knockout animals, including escape beats, tachycardia/bradycardia episodes and atrioventricular block. These sinus arrhythmias and conduction problems, reminiscent of arrhythmias observed in IDDCA subjects, further corroborate the involvement of *Gnb5* gene in altered cardiac function and irregular heartbeat. Finally, our mice study showed that *Gnb5*-inhibitory signalling is essential for the parasympathetic control of the HR as suggested by previous studies in other models.^1 7^ Knockout mice treated with a parasympathomimetic presented with bradycardia, while injection of an antiparasympathetic drug atropine had the same effect as in wild type. The β-adrenergic activity of *Gnb5* ^−/−^ mice was unaltered, suggesting a normal sympathetic modulation of the cardiac stimulation, and further confirming that the more efficient cardiac function is an adaptation to counteract the reduced size of the *Gnb5* ^−/−^ heart.

Consistent with this observation, transcriptome profiling provided insights into modifications of cardiac contraction properties, along with reduced ventricular expression of genes required for development of pacemaker cells. Instead of specific signals (eg, pacemaker function genes in atria), genes related to heart muscle contraction (eg, *Myh7* and *Lmod2* in atria, *Tnnt2* and *Lrrc10* in ventricles) and conduction system functions (eg, *Agt* and *Npr3* in atria, *Tbx18* in ventricles and *Snc10a* in both) were identified as DEGs in both atria and ventricles in *Gnb5* ^−/−^ animals. These transcriptome alterations may shed light on the mechanisms that result in the pathology of IDDCA. Approaches like single-cell RNA-sequencing combined with the isolation of sinoatrial node cells are warranted to complement our findings in the future. Globally, these data further challenge our study, since we cannot unravel whether the transcriptome modifications are cause or consequence of the augmented contractility. We argue, in line with the aforementioned results, that we are possibly investigating a compensatory mechanism. Interestingly, mice lacking *Gnb5* overexpress *Gnb3*, a gene encoding a different Gβ subunit and involved in the activation of GIRK channels.^82^ In contrast to *Gnb1* and *Gnb4* that have a well-documented role in the nervous system,^76 83^ *Gnb3*-null mice presented with cardiac manifestations, including slower HRs. However, their isolated hearts responded equivalently to muscarinic receptor-adrenergic and β-adrenergic receptor-stimulations, thus suggesting that *Gnb3* is unlikely to be involved directly in the G-protein signalling controlling heart pacemaker activity.^84^ Nonetheless, its higher expression may play a role in coping with the loss of *Gnb5*; further studies are warranted to elucidate such compensatory mechanisms. The expression of *Gnb2* is unchanged in the cardiac tissue of *Gnb5* knockouts; however, it was also linked to heart functions in mouse and human^66 69^ with *Gnb2* knockout mice having increased HR, and ECG trace revealing shortened RR and PQ intervals and ST segment (https://www.mousephenotype.org/data/genes/MGI:95784).^85^

The transcriptome data provided molecular data supporting an increased cardiac function of *Gnb5* ^−/−^ mice. It probably results from both a compensatory mechanism, as shown by misexpression of genes involved in cardiac muscle contractility, and an alteration of the expression of genes involved in the regulation of the HR. We also documented transcriptional changes in three brain regions of knockout mice which correlate with the IDDCA neuropsychiatric disease spectrum.

HR regulation by the parasympathetic and sympathetic branches of the autonomic nervous system takes place in the pacemaker cells of the sinoatrial node. While sympathetic modulation increases pacemaker cell firing rate, the vagal parasympathetic activity decreases the HR. Both activities are mediated by G-protein-coupled signalling on β-adrenergic (sympathetic) and cholinergic M_2_ muscarinic (parasympathetic) receptors, respectively. Autonomic innervation is regulated in vivo by RGS proteins. In particular, in the heart, the M_2_ receptor signalling is mediated by the Rgs6–Gnb5 complex.^17^ As *Gnb5* and *Rgs* genes are coexpressed and as the physical association of the encoded proteins is critical for complex stability, we expected to observe reduced *Rgs* expression levels. In a previous report,^17^ no Rgs6 was detected in the atria and in the brain of *Gnb5* knockout mice. Surprisingly, we detected a trend of increased expression of *Rgs* subunits, in particular *Rgs6*, contrary to findings in reference^7^ where expression of *RGS6* was not different between GNB5-Ser81Leu and wild-type hiPSCs. These discrepancies may stem from the fact that we are investigating different model systems, that is, mouse tissues versus human cells, as well as by the use of different technologies to detect mRNA abundance; that is, RNA-sequencing versus qRT-PCR and that in case of hiPSCs data were generated from one clone per genotype.

Altogether, our results unveil that potentially compensatory changes occur at a transcriptional level in *Gnb5* ^−/−^ mice through higher expression of *Gnb3* and *Rgs* genes in heart tissues. Additional evidence at protein level did not confirm compensatory changes. The previously cited link between *GNB5* and the R7-RGS *RGS6* and *RGS11* suggests that they are part of the same pathway. Consistent with this hypothesis, the members of a Tunisian family presenting with cataract, mental retardation and microcephaly were carriers of biallelic mutations in *RGS6* 86. Another unpublished case harbouring mutation in *RGS11* presented with overlapping neuropsychiatric phenotype (Sarah Montgomery, *in litt*). No specific cardiac evaluation is reported in these individuals, but our data suggest that this should be considered. Co-occurrence of neuropsychiatric symptoms with visual and cardiac manifestations could represent a unique combination associated with Gβ and the R7-RGS related proteins. We hypothesise that the disease mechanism responsible for HR perturbations is the reduction or the loss of negative regulation by Rgs6 on the inhibitory Gβ_5_ signalling, resulting in enhanced parasympathetic activity.

Overall, our work highlights that *Gnb5* ^−/−^ mice not only recapitulate IDDCA’s neurological manifestations previously outlined elsewhere^22^ but also mimic its cardiac perturbations, especially regarding heart arrhythmias and autonomic nervous system control, allowing for future screening of drugs modulating the parasympathetic branch of the autonomic nervous system, in view of the development of patients’ therapy.

## Web resources

GnomAD: https://gnomad.broadinstitute.org/about

IMPC: http://www.mousephenotype.org/MutationTaster2: http://www.mutationtaster.org/

PolyPhen-2: http://genetics.bwh.harvard.edu/pph2/index.shtml

PROVEAN: http://provean.jcvi.org/index.phpSIFT: http://sift.jcvi.org/

UMD Predictor: http://umd-predictor.eu/

Functional Analysis through Hidden Markov Models V.2.3: http://fathmm.biocompute.org.uk/

Splice Site Prediction by Neural Network (NNSplice): https://www.fruitfly.org/seq_tools/splice.html

NetGene2 server: http://www.cbs.dtu.dk/services/NetGene2/

Human Splicing Finder: http://www.umd.be/HSF/

CADD: https://cadd.gs.washington.edu/

Greater Middle East Variome Project: http://igm.ucsd.edu/gme/

## Accession Numbers

The RNA-seq data have been deposited in the Gene Expression Omnibus database under accession number GSE156898.

10.1136/jmedgenet-2020-107015.supp1 Supplementary data

10.1136/jmedgenet-2020-107015.supp5 Supplementary data

## Data Availability

Data are available in a public, open access repository. The RNA-seq data have been deposited in the Gene Expression Omnibus database under accession number GSE156898.
