# Supplementary material for: Inhibition of G-protein signalling in cardiac dysfunction of intellectual developmental disorder with cardiac arrhythmia (IDDCA) syndrome
Source: J Med Genet. 2020 Nov 10;58(12):815–31. doi: 10.1136/jmedgenet-2020-107015 (PMC8639930; doi:10.1136/jmedgenet-2020-107015)
Supplement: Supplementary data [file jmedgenet-2020-107015supp003.pdf]

Figure S1

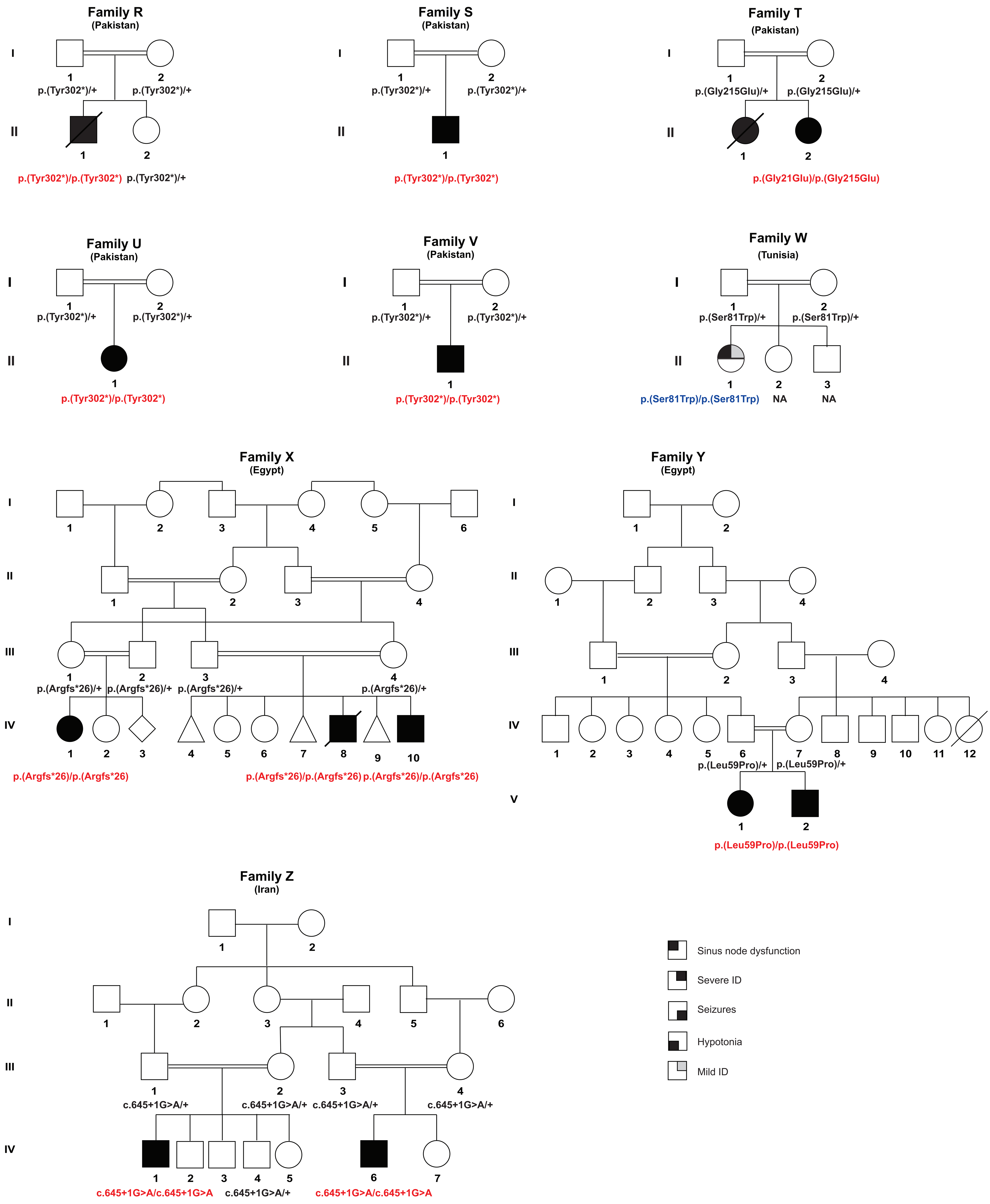

Figure S1. IDDCA and LADCI family trees. Pedigrees of the nine consanguineous families described in this study; affected individuals carry homozygous GNB5 variants inherited from heterozygous parents. Filled symbols represent individuals with severe sinus node dysfunction (top left quarter), intellectual disability (ID, top right quarter), seizures (bottom right quarter) and hypotonia (bottom left quarter). The light grey top quarter indicates the occurrence of mild ID. The affected individuals of families T, W and Y harbor novel variants which are modeled in panel A of **Figure 1**. The variants of IDDCA affected individuals are displayed in red (LoF), whereas missense LADCI variants are in blue.

Figure S2

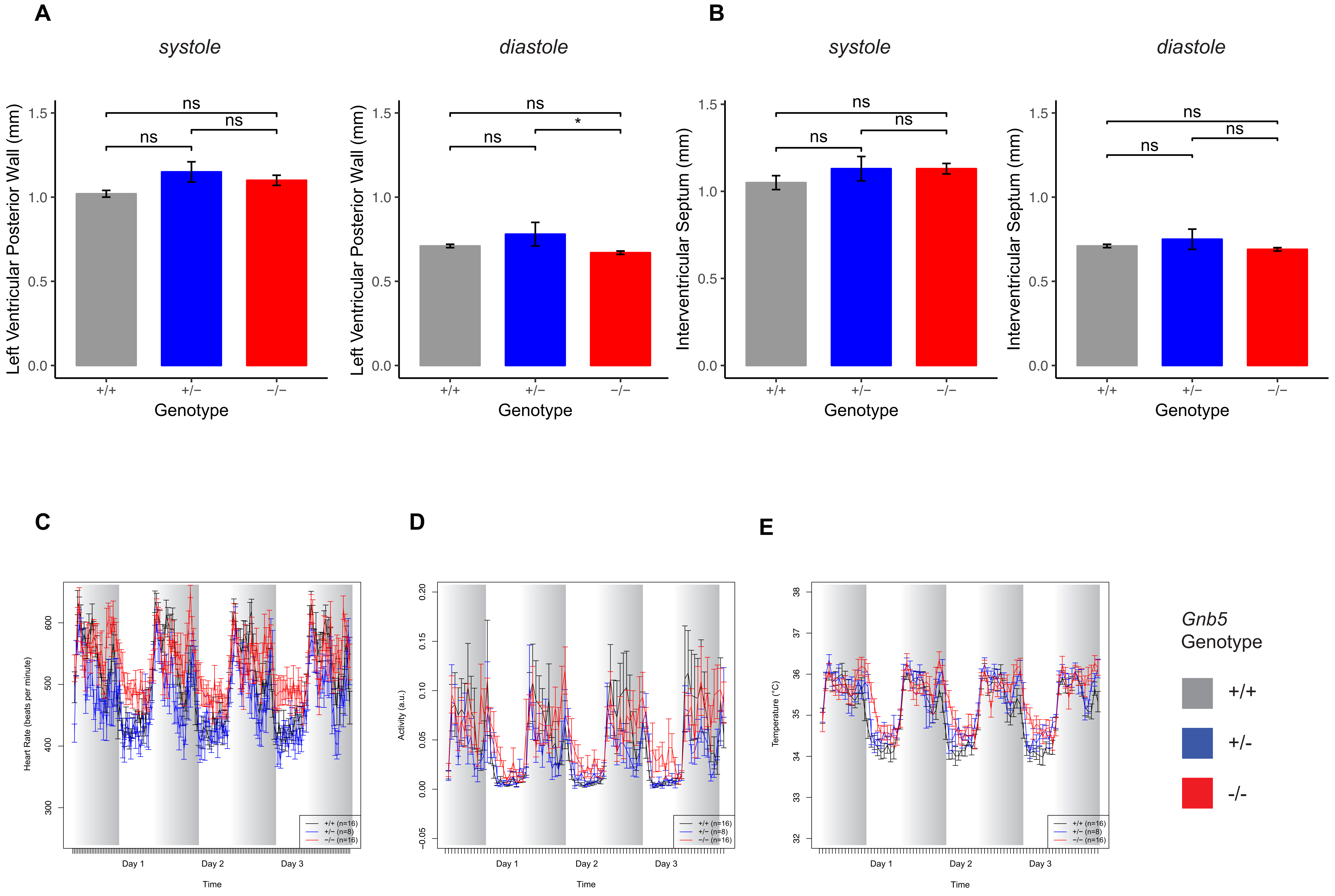

Figure S2: **Unchanged morphological parameters measured by echocardiography.** (**A**) Cardiac wall thickness in systole (right) and diasto-le (left). (**B**) Interventricular septum (at systole and diastole). (**C**) Heart rate measured in baseline condition through *in vivo* ECG. Values of knock-out (red) mice show a trend toward increased heart rate both during day and night (grey windows). (**D**) Qualitative measure of the mouse activity, consisting in displacement of the telemetric device. *Gnb5*$^{-/-}$ animals seem to be more active during the day, usually representing the sleeping phase for a mouse. (**E**) Measurements of the body temperature showing a similar trend to the activity (significance between knock-out and wild-type mice oscillating between p = 1.35E-04 and p = 0.9163 in the course of 36 daylight time points).

Figure S3

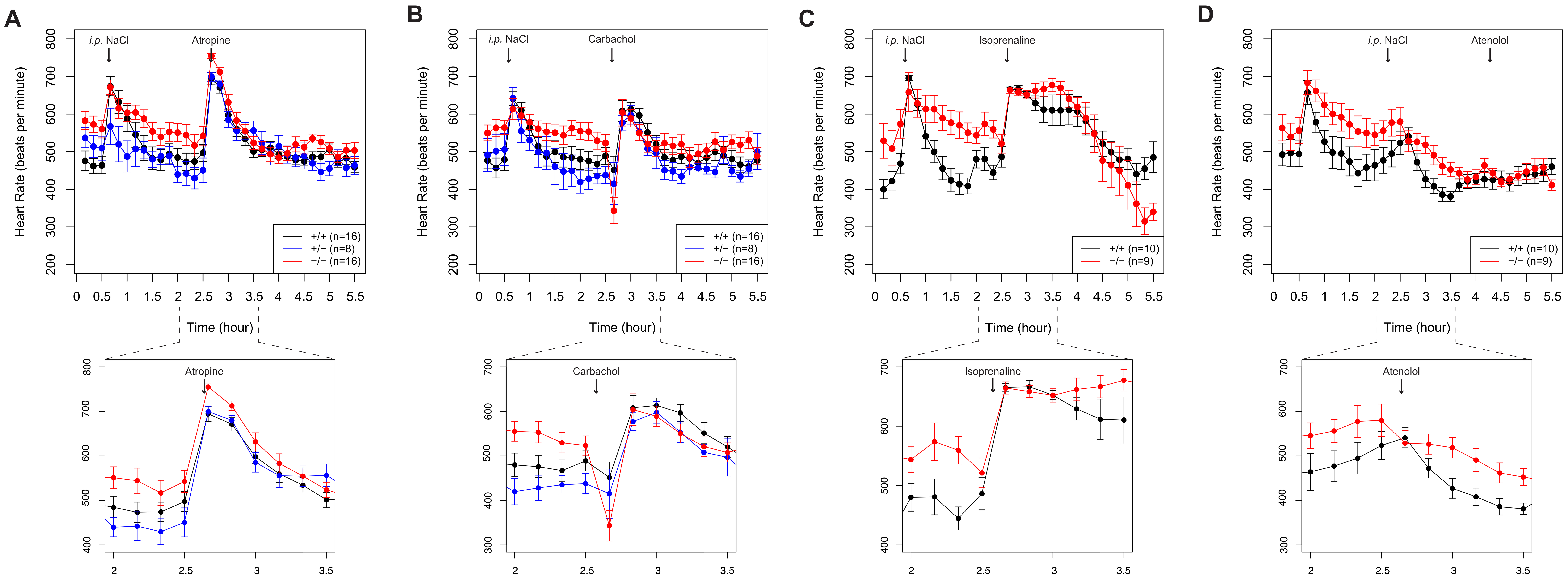

Figure S3: **Pharmacological challenges expressed in raw values.** Parasympathetyc (**A-B**) and sympathetyc (**C-D**) stimulation expressed in raw values (top). Zoom in on times ~ 2 to 3.5 hours illustrating peak and nadir heart rate differences among the genotypes (bottom).

Figure S4

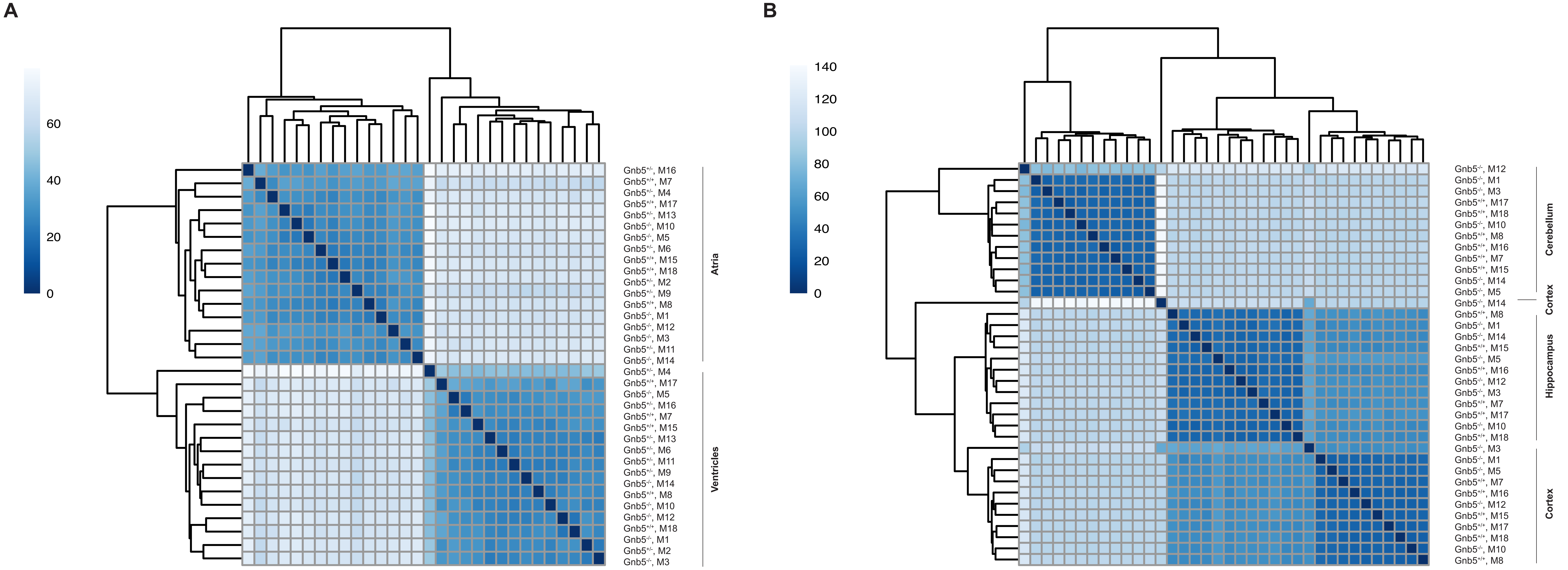

Figure S4: Samples distance of RNA samples. Heat map depicting hierarchical clustering of sample-to-sample distances for heart tissues (**A**) and brain regions (**B**) using normalized RNA-seq gene read counts. Blue color represents the nearest distance and light grey color indicates long sample-sample distance. Samples are clustered by tissue: (**A**) atria (top), and ventricles (bottom); (**B**) cerebellum (top), hippocampus (middle), and cortex (bottom). Samples M12-cerebellum, M14-cortex and M3-cortex were excluded from the analysis due to pronounced 3’ mRNA degradation observed at the quality control step.

BMJ Publishing Group Limited (BMJ) disclaims all liability and responsibility arising from any reliance
placed on this supplemental material which has been supplied by the author(s)

[redacted]

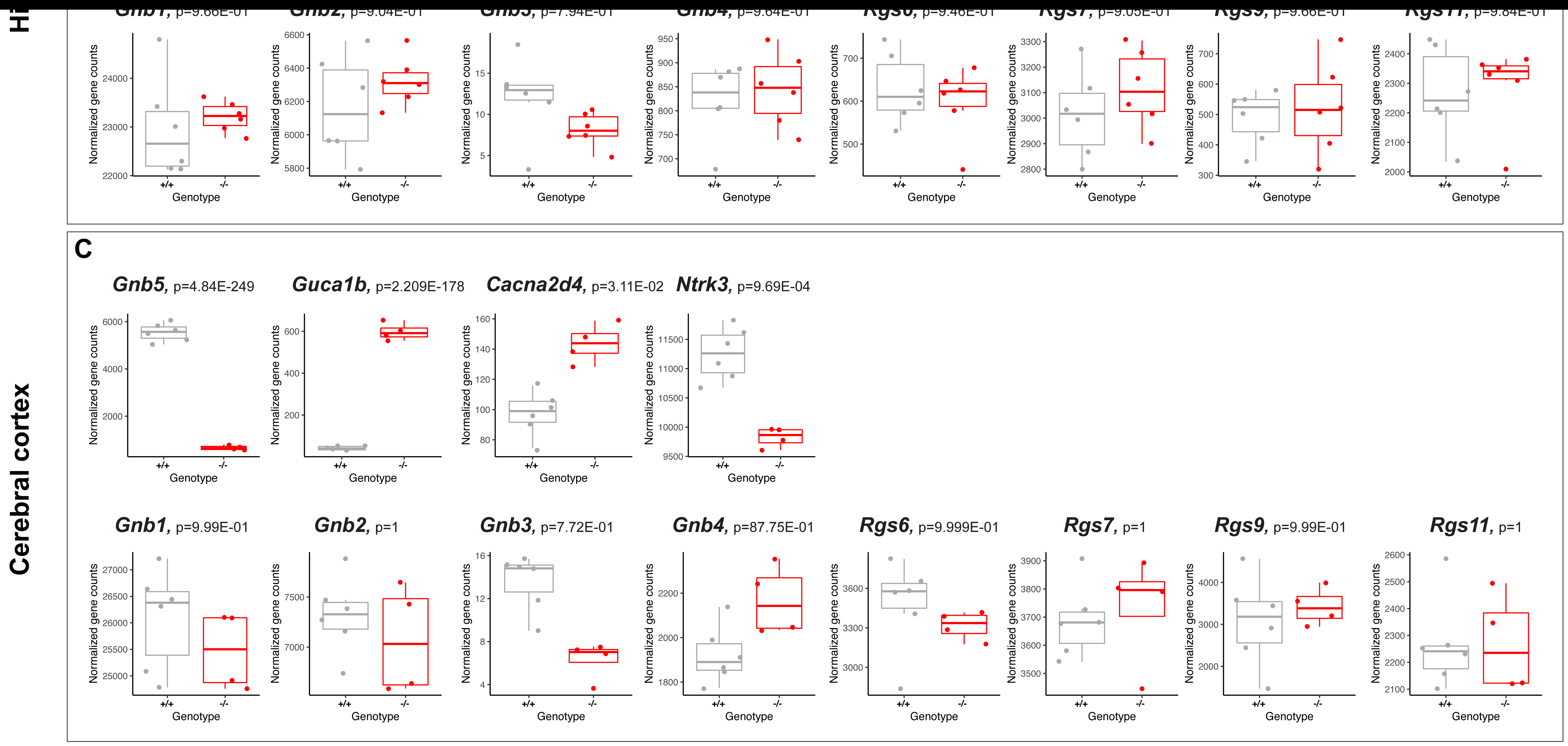

Figure S5: Expression profile of relevant genes in cerebellum, hippocampus and cerebral cortex of *Gnb5*$^{-/-}$, *vs. Gnb5*$^{+/+}$ mice. Expression levels of *Gnb5* (top left in each panel) and other differentially expressed genes in cerebellum (**A**, top), hippocampus (**B**, top) and cerebral cortex (**C**, top); *Gnb* and *Rgs* transcripts quantification in cerebellum (**A**, bottom), hippocampus (**B**, bottom) and cerebral cortex (**C**, bottom).

BMJ Publishing Group Limited (BMJ) disclaims all liability and responsibility arising from any reliance
placed on this supplemental material which has been supplied by the author(s)

Figure S6

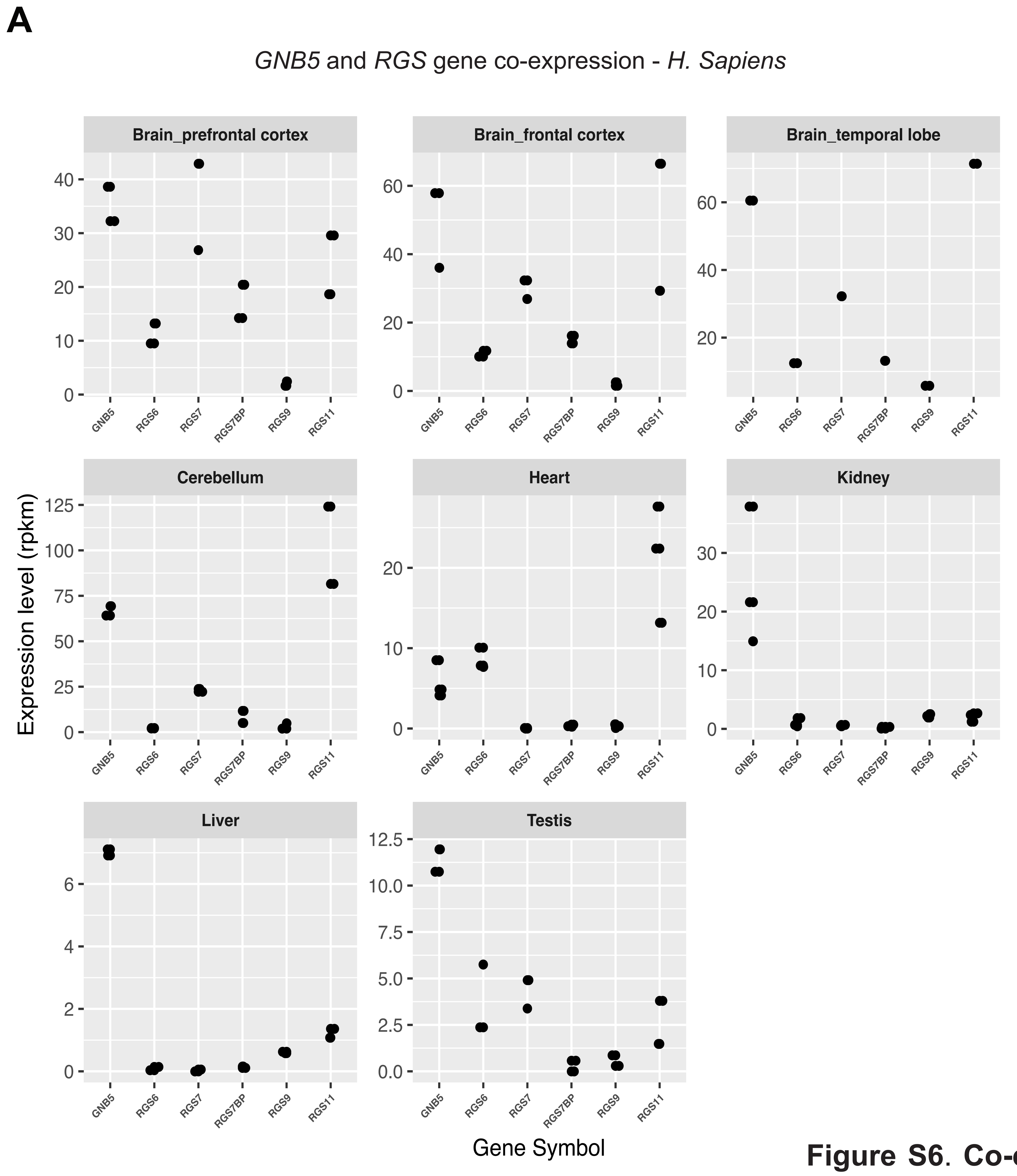

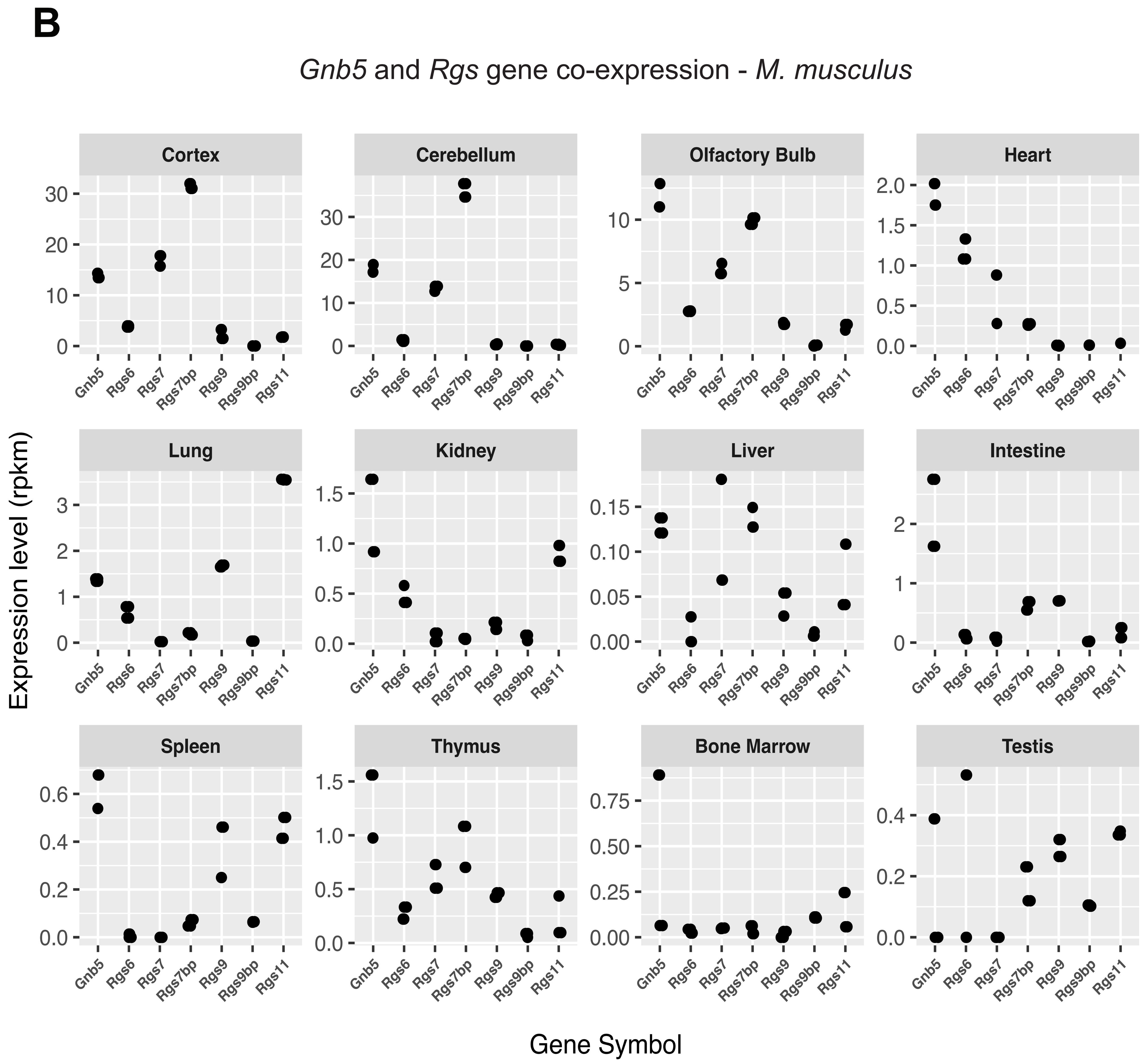

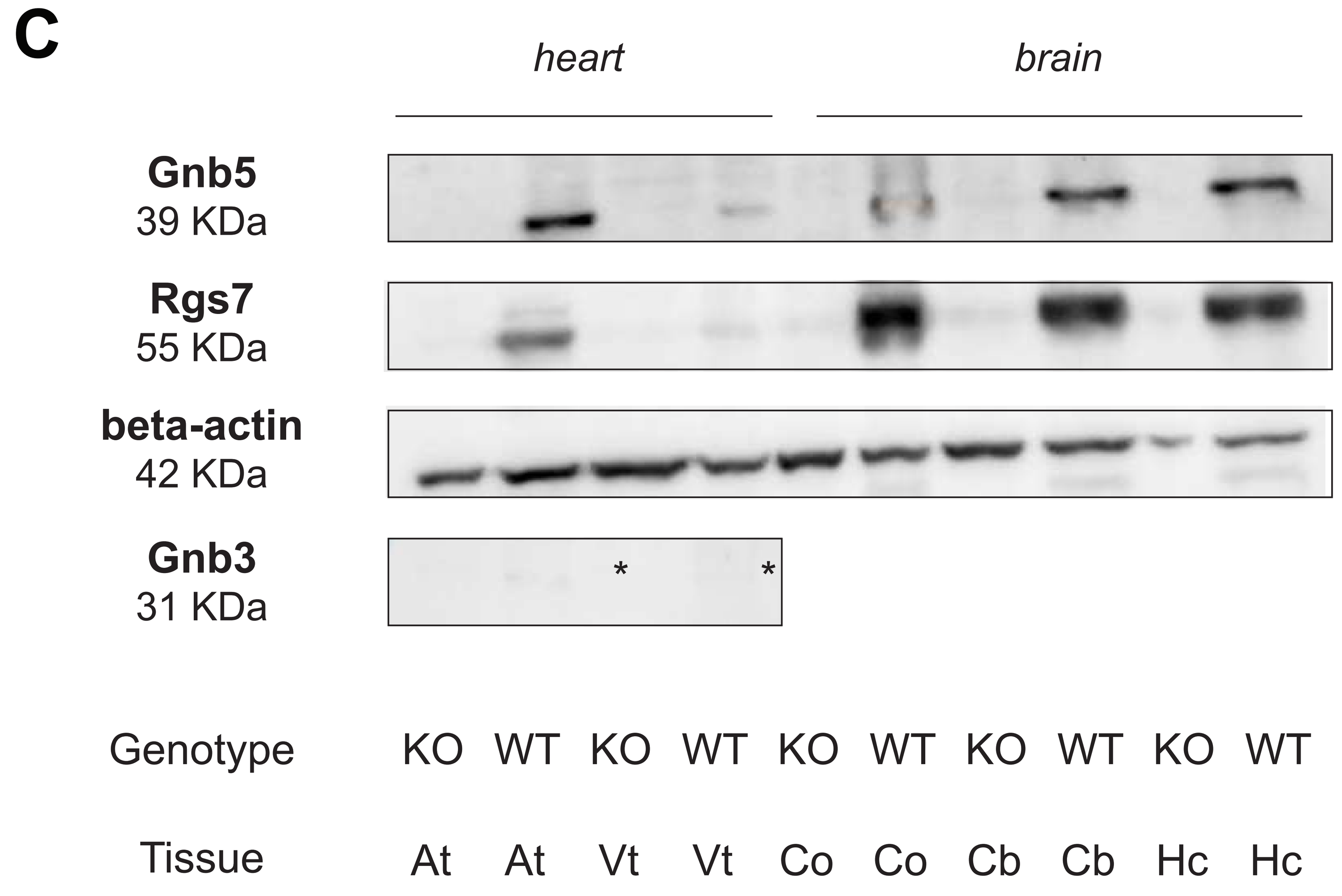

Figure S6. **Co-expression of Gnb5 and R7-Rgs genes in human and mouse.** *Gnb5* and *Rgs* genes expression was evaluated using publicly available transcritpome datasets in human (**A**) and mouse (**B**). Human data were re-trieved from (Brawand D. Nature. 2012); gene expression files for mouse tissues were obtained through the ENCODE website. Each point in the plots represents a replicate. Genes encoding for the R7 family of RGS proteins, known partners of GNB5, were inspected. In human, *GNB5* shows co-expression with *RGS7* and *RGS11* in the brain regions, while in heart co-epxression occurs between *GNB5* and *RGS6* and *RGS11*. A similar trend is observed in mouse, with *Gnb5* being co-expressed with *Rgs7* in brain, and with *Rgs6* in heart. Accordingly, *RGS9* is only expressed in eye, therefore we do not capture any co-expression profile with this subunit. (**C**) Gnb5, Rgs7, and Gnb3 protein levels across heart and brain mouse tissues by Western blotting. Beta-actin antibody was used as a loading control. ”KO” and “WT” indicate tissues harvested from *Gnb5*$^{-/-}$ and *Gnb5*$^{+/+}$ mice, respectively (At = Atria, Vt = Ventricles, Co = Cortex, Cb = Cerebellum, Hc = Hippocampi). Asterisks indicate the faint signal corresponding to Gnb3 protein expression.
