# Supplementary material for: Inhibition of G-protein signalling in cardiac dysfunction of intellectual developmental disorder with cardiac arrhythmia (IDDCA) syndrome
Source: J Med Genet. 2020 Nov 10;58(12):815–31. doi: 10.1136/jmedgenet-2020-107015 (PMC8639930; doi:10.1136/jmedgenet-2020-107015)
Supplement: Supplementary data [file jmedgenet-2020-107015supp004.pdf]

Table S1. Detailed clinical features of individuals with IDDCA and LADCI syndromes

	Lodder E., De Nittis P., Koopman C. *et al.*, 2016								
	**Family A**		**Family B**	**Family C**		**Family D**	**Family E**		**Family F**
**Individual**	**1**	**2**	**3**	**4**	**5**	**6**	**7**	**8**	**9**
**Gender, Age (years)**	F, 22	F, 20	F, 6	F, 11	M, 9	F, 12	F, 13	M, 8	M, 23
**Nucleotide change (NM_006578.3)**	c.249G>A,r.249_250 ins249+1_249+25/ c.994C>T	c.249G>A,r.249_250 ins249+1_249+25/ c.994C>T	c.249+1G>T/ c.249+1G>T	c.249+3G>T/ c.249+3G>T	c.249+3G>T/ c.249+3G>T	c.906C>G/ c.906C>G	c.242C>T/ c.242C>T	c.242C>T/ c.242C>T	c.242C>T/ c.242C>T
**Amino acid change (NP_006569.1)**	p.Asp84Valfs*52/ p.(Arg332*)	p.Asp84Valfs*52/ p.(Arg332*)	p.Asp84Leufs*31/ p.Asp84Leufs*31	p.Asp84Valfs*31/ p.Asp84Valfs*31	p.Asp84Valfs*31/ p.Asp84Valfs*31	p.Tyr302*/ p.Tyr302*	p.(Ser81Leu)/ p.(Ser81Leu)	p.(Ser81Leu)/ p.(Ser81Leu)	p.(Ser81Leu)/ p.(Ser81Leu)
**Birth weight**	3580 g (50th percentile)	NA	NA	2751 g (15 th percentile)	NA	2845 g (15th percentile)	NA	NA	NA
**Ethnicity**	Italy	Italy	Jordan	Puerto Rico	Puerto Rico	India	Morocco	Morocco	Brazil
**Consanguinity**	–	–	+	+	+	–	–	–	+
**Altered speech development**	+	+	NR	+	+	+	+	+	NA
**- Verbal understanding**	NA	NA	nonverbal	unremarkable	unremarkable	NA	NA	NA	NA
**- Lexical production**	NA	NA	nonverbal	delayed	delayed	nonverbal	delayed	delayed	NA
**Intellectual disability (ID)**	+	+	+	+	+	+	mild	mild	mild
**Epilepsy**	+	+	+	-	-	+	-	-	-
**Sinus Sick Syndrome (SSS)**	+	+	+	+	+	increased PR interval (intermittent Weckenbach)	+	+	+
**- Minimum heart rate**	24	39	NA	paced	paced	NA	20	16	NA
**- Maximum heart rate**	163	192	NA	paced (27% heartbeats on Holter)	paced (20% heartbeats on Holter)	NA	176	180	NA
**- Chronotropic response**	NA	NA	NA	+	+	NA	unremarkable	unremarkable	NA
**- Escape beats**	+	+	NA	paced	paced	NA	+	+	NA
**Pacemaker implantation**	-	-	-	+	+	-	-	+	NA
**Heart structural abnormalities**	-	PFO	NA	-	-	-	-	-	NA
**Hypotonia**	+	+	+	+	+	+	-	impaired fine motor skills	-
**Pathological gastric reflux**	+	+	NA	+	+	+	-	-	NA
**Nystagmus**	+	+	+	+	+	+	NA	-	NA
**Retinal Disease**	+	NA	NA	NA	NA	+	NA	NA	NA
**Metabolic work-up:** **- Plasma amino acids chromatography**	938 µm/l (restored)	+ (restored)	NA	unremarkable	unremarkable	unremarkable	444 µm/l	unremarkable	NA
**- Urine organic acids**	unremarkable	unremarkable	increased excretion of 3-methyl-glutaconic acid	unremarkable	unremarkable	unremarkable	NA	NA	NA
**Others**	-	-	-	-	-	-	-	-	-
**Dysmorphic feature(s)**	-	-	-	-	-	-	-	-	-
**Family history**	-	-	-	-	-	-	-	-	-

	Shamseldin *et al.*, 2016					Turkdogan D. *et al.*, 2016					
	**Family G**					**Family H**					
**Individual**	**10**	**11**	**12**	**13**	**14**	**15**	**16**	**17**	**18**	**19**	**20**
**Gender, Age (years)**	F, 10	F, 9	F, 3	F, 5	F, 9	M, 2 (second degree cousin of pt 16)	F, 10 - pair of a monozygotic twin	F (deceased 5.5months)	M (deceased 7months)	F - pair of a monozygotic twin (deceased 8months)	M (deceased 7yo)
**Nucleotide change (NM_006578.3)**	c.242C>T/ c.242C>T	c.242C>T/ c.242C>T	c.242C>T/ c.242C>T	c.242C>T/ c.242C>T	c.242C>T/ c.242C>T	c.355delG/ c.355delG	c.355delG/ c.355delG	c.355delG/ c.355delG	c.355delG/ c.355delG	c.355delG/ c.355delG	c.355delG/ c.355delG
**Amino acid change (NP_006569.1)**	p.(Ser81Leu)/ p.(Ser81Leu)	p.(Ser81Leu)/ p.(Ser81Leu)	p.(Ser81Leu)/ p.(Ser81Leu)	p.(Ser81Leu)/ p.(Ser81Leu)	p.(Ser81Leu)/ p.(Ser81Leu)	p.Ala119Profs16/ p.Ala119Profs16*	p.Ala119Profs16/ p.Ala119Profs16*	p.Ala119Profs16/ p.Ala119Profs16*	p.Ala119Profs16/ p.Ala119Profs16*	p.Ala119Profs16/ p.Ala119Profs16*	p.Ala119Profs16/ p.Ala119Profs16*
**Birth weight**	50th centile	50th centile	NA	50th centile	NA	NA	1800 g (<1st)	NA	NA	NA	NA
**Ethnicity**	Saudi	Saudi	Saudi	Saudi	Saudi	Turkish	Turkish	Turkish	Turkish	Turkish	Turkish
**Consanguinity**	+	+	+	-	-	+	+	+	+	+	+
**Altered speech development**	+	+	+	-	-	+	+	NA	NA	NA	NA
**- Verbal understanding**	+	+	+	+	+	no developmental milestones	no developmental milestones	NA	NA	NA	NA
**- Lexical production**	delayed	delayed	delayed	delayed	delayed	no developmental milestones	no developmental milestones	NA	NA	NA	NA
**Intellectual disability (ID)**	normal IQ, but school performance issues	normal cognitive development	NA	normal IQ	NA	+	+	+	+	+	+
**Epilepsy**	NA	NA	NA	NA	NA	+	+	NA	+	+	NA
**Sinus Sick Syndrome (SSS)**	NA	NA	NA	NA	NA	+	+	NA	NA	NA	+
**- Minimum heart rate**	NA	NA	NA	NA	NA	NA	NA	NA	NA	NA	NA
**- Maximum heart rate**	NA	NA	NA	NA	NA	NA	NA	NA	NA	NA	NA
**-Chronotropic response**	NA	NA	NA	NA	NA	NA	NA	NA	NA	NA	NA
**- Escape beats**	NA	NA	NA	NA	NA	NA	NA	NA	NA	NA	NA

**Pacemaker implantation**	NA	NA	NA	NA	NA	– (suggested, but refused by parents)	– (suggested, but refused by parents)	NA	NA	NA	NA
**Heart structural abnormalities**	NA	NA	NA	NA	NA	–	–	NA	NA	NA	NA
**Hypotonia**	-	-	NA	+	NA	+	+	NA	NA	NA	NA
**Pathological gastric reflux**	NA	NA	NA	NA	NA	NA	NA	NA	NA	NA	NA
**Nystagmus**	NA	NA	NA	NA	NA	+	+ (horizontal)	NA	NA	NA	NA
**Retinal Disease**	NA	NA	NA	NA	NA	+	+	NA	NA	NA	NA
**Metabolic work-up -Plasma amino acids chromatography**	NA	NA	NA	NA	NA	NA	unremarkable	NA	NA	NA	NA
**-Urine organic acids**	NA	NA	NA	NA	NA	NA	unremarkable	NA	NA	NA	NA
**Others**	ADHD, marked hyperactivity	inattentive ype ADHD	-	motor delay	ADHD, mild motor delay	-	autistic (midline hand automatisms, lack of eye contact)	NA	NA	NA	NA
**Dysmorphic feature(s)**	-	-	-	-	-	-	prominent forehead, acquired micro-brachycephaly	NA	NA	NA	NA
**Family history**	-	-	-	-	-	-	-	NA	NA	NA	NA

	Vernon H. *et al.*, 2017	Malerba N. *et al.*, 2018	Poke G. *et al.*, 2019					Tang M. *et al.*, 2020	Yadzani S. *et al.*, 2020		Sciacca F.L *et al.*, 2020
	**Family I**	**Family J**	**Family K**	**Family L**	**Family M**		**Family N**	**Family O**	**Family P**		**Family Q**
**Individual**	**21**	**22**	**23**	**24**	**25**	**26**	**27**	**28**	**29**	**30**	**31**
**Gender, Age (years)**	M, 2	F, 2.5	M, 10	M, 3	F (deceased 13yo)	F, 2	F, 3	M, 6 months	M, 22	M, 18	M
**Nucleotide change (NM_006578.3)**	c.222_226delTAAA/ c.737G>A	c.222_226delTAAGA/ c.242C>T	c.136delG/ c.136delG	c.906C>G/ c.906C>G	c.242C>A/ c.242C>A	c.242C>A/ c.242C>A	c.906C>A/ c.906C>A	c.332G>A/ c.906C>A	c.906C>G/ c.906C>G	c.906C>G/ c.906C>G	Arr[Hg19} 15q21.2: (52385564_ 52579282)x0
**Amino acid change (NP_006569.1)**	p.Asp74Glufs*52/ p.(Arg246Gln)	p.Asp74Glufs* 52/ p.(Ser81Leu)	p.Glu46fs*68/ p.Glu46fs*68	p.Tyr302*/ p.Tyr302*	p.Ser81*/ p.Ser81*	p.Ser81*/ p.Ser81*	p.Tyr302*/ p.Tyr302*	p.Cys111Tyr/ p.Tyr302*	p.Tyr302*/ p.Tyr302*	p.Tyr302*/ p.Tyr302*	/
**Birth weight**	3,311 g (50th)	1698 g (< 1st percentile)	NA	NA	NA	NA	NA	2700 g	3000 g (15th-25th percentile)	2540 g (3rd-15th percentile)	NA
**Ethnicity**	European/Caucasian	European/Caucasian	Cambodia	Pakistan	Algeria	Algeria	Pakistan	China (Han)	Pakistan	Pakistan	Egypt
**Consanguinity**	–	-	+ (second cousins)	+ (first cousin once removed)	+ (first cousins)	+ (first cousins)	-	-	+ (first cousins)	+ (first cousins)	+ (first cousins)
**Altered speech development**	+	+	+	+	+	+	+	NA	+	+	+
**- Verbal understanding**	nonverbal	+	nonverbal	nonverbal	nonverbal	nonverbal	nonverbal	NA	+	+	+
**- Lexical production**	nonverbal	expressive speech delay (spoken vocabulary of ~ 12 words)	nonverbal	nonverbal	nonverbal	nonverbal	nonverbal	NA	+	+	+
**Intellectual disability (ID)**	+	mild	+	+	+	+	+	+	+	+	+
**Epilepsy**	–	-	+	+	+	+	+	+	+	-	+
**Sinus Sick Syndrome (SSS)**	+	+	+	+	normal ECG	+	+	+	+	+	+
**- Minimum heart rate**	71	36	NA	NA	NA	NA	NA	NA	NA	NA	39
**- Maximum heart rate**	183	176	NA	NA	NA	NA	NA	NA	NA	NA	NA
**- Chronotropic response**	NA	NA	NA	NA	NA	NA	NA	NA	NA	NA	NA
**- Escape beats**	+ (prior to pacing)	+	+	+	NA	NA	NA	NA	+	NA	NA
**- Pacemaker implantation**	+	+	NA	NA	NA	NA	NA	NA	+	-	-
**- Heart structural abnormalities**	–	-	NA	NA	NA	NA	NA	NA	NA	NA	PFO and ductus arteriosus (spontaneously closed)
**Hypotonia**	+	+	+ (contractures, nonambulatory, no head control)	+ nonambulatory	+ nonambulatory	+ nonambulatory	+ nonambulatory	+	+ (lost gross- and fine- motor functions)	NA	+
**Pathological gastric reflux**	+	-	+	NA	NA	NA	NA	+	+	+ (rare and mild)	+
**Nystagmus**	+	strabismus (surgically corrected at 16 mo)	NA	+	+	+	+	NA	+ (lateral gaze nystagmus), strabismus	+ (lateral gaze nystagmus), strabismus, poor eye fixation	+
**Retinal Disease**	+	-	+	NA	NA	+	+	NA	NA	NA	+
**Metabolic work-up - Plasma amino acids chromatography**	NA	NA	NA	NA	NA	NA	NA	NA	NA	NA	NA

**- Urine organic acids**	NA	NA	NA	NA	NA	NA	NA	NA	NA	NA	NA
**Others**	left-sided hearing loss, intermittent extremity hypertonia, intermittent upper extremity jerking motions - sometimes in conjunction with a stimulus, laryngomalacia hypertonia, clenched hands, high-voltage cortical activity (EEG), pain of unknown etiology thin corpus callosum (brain MRI)	bilateral tympanostomy tubes; high activity level and short attentions span compared to peers	pyloric stenosis, G-tube, scoliosis	NA	NA	NA	long posterior corpus callosum, central sleep apnea	Adams-Stokes syndrome	cerebral palsy; spasticity of upper and lower extremities, erratic sleep pattern; G-tube feeding; food allergy; intermittent urinary retention	Indication of optic nerve atrophy (small, anomalous, tilted, pale discs)	bilateral ventricular and cerebral sulci enlargement on MRI; episodic bradypnea
**Dysmorphic feature(s)**	NA	plagiocephaly associated with torticollis at 8 mo (solved with molding helmet therapy)	NA	microcephaly	NA	NA	NA	-	-	-	-
**Family history**	maternal uncle and paternal first-cousin suspected to have ASD	NA	NA	NA	NA	NA	-	-	one sibling (individual 30) with similar phenotype	-	parental couple experienced five previous miscarriages

	This study												
	**Family R**	**Family S**	**Family T**	**Family U**	**Family V**	**Family W**	**Family X**			**Family Y**		**Family Z**	
**Individual**	**32**	**33**	**34**	**35**	**36**	**37**	**38**	**39**	**40**	**41**	**42**	**43**	**44**
**Gender, Age (years)**	M, 8 months (deceased)	M, 15	F, 1.2	F, 4	2, M	F, 7	F, 5	M deceased (7 yo)	M, 2	F, 19	M, 16	M, 8	M, 6
**Nucleotide change (NM_006578.3)**	c.906C>G / c.906C>G	c.906C>G/ c.906C>G	c.644G> A/ c.644G> A	c.906C>A / c.906C>A	c.906C>G/ c.906C>G	c.242C>G/ c.242C>G	c.88_89ins CTCAA/ c.88_89ins CTCAA/	c.88_89ins CTCAA/ c.88_89ins CTCAA/	c.88_89ins CTCAA/ c.88_89ins CTCAA/	c.176T>C/ c.176T>C	c.176T>C/ c.176T>C	c.645+1G >/ c.645+1G>A	c.645+1G >/ c.645+1G>A
**Amino acid change (NP_006569.1)**	p.Tyr302*/ p.Tyr302*	p.Tyr302*/ p.Tyr302*	p.(Gly215Glu)/ p.(Gly215Glu)	p.Tyr302*/ p.Tyr302*	p.Tyr302*/ p.Tyr302*	p.(Ser81Trp)/ p.(Ser81Trp)	p.Argfs*26/ p.Argfs*26	p.Argfs*26/ p.Argfs*26	p.Argfs*26/ p.Argfs*26	p.(Leu59Pro)/ p.(Leu59Pro)	p.(Leu59Pro)/ p.(Leu59Pro)	p.?	p.?
**Birth weight**	NA	NA	NA	NA	NA	2900 g	3000 g	2800 g	2500 g	2600 g	2500 g	NA	NA
**Ethnicity**	Pakistan	Pakistan	Pakistan	Pakistan	Pakistan	Tunisia	Egypt	Egypt	Egypt	Egypt	Egypt	Iran	Iran
**Consanguinity**	+	+	+	+	+	+ (first cousins)	+ (first cousins)	+ (first cousins)	+ (first cousins)	+ (first cousins)	+ (first cousins)	+ (first cousins)	+ (first cousins)
**Altered speech development**	+	+	+	-	+	+	+	+	+	+	+	+	+
**- Verbal understanding**	+	+	no developmental milestones	unremarkable	NA	low	nonverbal	nonverbal	nonverbal	nonverbal	nonverbal	no developmental milestones	delayed
**- Lexical production**	+	+	no developmental milestones	unremarkable	delayed (vocalization at 1.10yo)	nonverbal	vocalized	vocalized	nonverbal	meaningless single syllable words	vocalized	nonverbal	NA
**Intellectual disability (ID)**	+	+	-	+	NA	+ (no neuro-psychological assessment)	+	+	+	+	+	+	+
**Epilepsy**	-	+	-	-	+	normal EEG	+	+	+	+	+	+	+
**Sinus Sick Syndrome (SSS)**	NA (inconsistent heart rate noted at Echo)	NA	+	NA	NA	+	NA	NA	NA	+	+	NA	NA
**- Minimum heart rate**	NA	NA	18	32	29	30	NA	NA	NA	NA	NA	NA	NA
**- Maximum heart rate**	NA	154	142	152	158	160	NA	NA	NA	NA	NA	NA	NA
**- Chronotropic response**	NA	NA	NA	NA	NA	NA	NA	NA	NA	NA	NA	NA	NA
**- Escape beats**	NA	-	+	NA	NA	+	NA	NA	NA	NA	+	NA	NA
**Pacemaker implantation**	-	-	-	-	-	-	-	-	-	-	-	-	-
**Heart structural abnormalities**	-	-	NA	NA	-	-	small atrial septal defect	-	small atrial septal defect secundum with minimal left to right shunt; small PDA	-	-	NA	NA
**Hypotonia**	+	+ (no head control)	+ (no neck holding)	+ (no neck holding)	+ (along with hyporeflexia)	+ (impaired fine motor skills)	+	+	+	+	+	+	+

**Pathological gastric reflux**	NA	+	-	NA	-	-	+	-	+	+	+	+	+
**Nystagmus**	_	+	+	no eye contact	no eye contact, convergent squint (strabismus) right eye	-	-	+	+	+ (mild)	+ (mild)	+ (strabismus, horizontal nystagmus)	+ (strabismus, horizontal nystagmus)
**Retinal Disease**	NA	NA	-	NA	myopia (-1.5)	-	-	+	+	+ (reduced pigmentation)	+ (reduced pigmentation)	-	-
**Metabolic work-up - Plasma amino acids chromatography**	NA	non specific	unremarkable	unremarkable	unremarkable	global hypoAA, High Phe (146, N: 39-77)	unremarkable	unremarkable	unremarkable	unremarkable	unremarkable	unremarkable	NA
**- Urine organic acids**	NA	unremarkable	NA	Fatty Acid Oxidation Disorder	unremarkable	normal	unremarkable	unremarkable	unremarkable	unremarkable	unremarkable	unremarkable	NA
**Others**	-	thining, hypogenesis of corpus callosum, modified hypsarrhythmia breath holding spell, taking EPIVAL (VAP) and fits are controlled	-	cerebral Atrophy on MRI	-	unexplained coma secondary to sepsis in the context of digestive infection; one episode of pyelonephritis without complications	hypogenesis of corpus callosum on MRI	autistic behavior	prominent ventricular system on MRI; dysmyelination	thin dysplastic corpus callosum, abnormal increased cortical gyration (polymicrogyria) deep white matter lesions defective myelination	thin dysplastic corpus callosum, abnormal increased gyration (polymicrogyria) white matter lesions defective myelination	muscle atrophy	behavioral anomalies (laughing and hand stereptypies)
**Dysmorphic feature(s)**	macrocephaly	-	-	deep philtrum, micrognathia, prominent ears, disproportion of skull to face	blond hair, frontal bossing, epicanthic folds, small palpebral fissure, depressed nasal bridge, high arched palate	shorter thumbs	hypotonic face, with open mouth, downslanting palpebral fissures, low set ears	NA	prominent forehead, synophrys, downslanting palpebral fissures, depressed nasal root, low set ears	long hypotonic face, with open mouth, arched eye brows, mild ptosis, prominent nose, upturned, long philtrum, thin lips, broad chin, low set ears	long hypotonic face, with open mouth, arched eye brows, mild ptosis, prominent nose, upturned, long philtrum, thin lips, broad chin, low set ears	NA	NA
**Family history**	respiratory stridor	one sibling with similar phenotype died, likely due to cardiac arrest	-	-	paternal uncle with severe cognitive impairment and no language; maternal aunt and uncle are severely handicapped and no language, similar to the son of another maternal aunt	-	two cousins (individuals 40 and 41) with similar phenotype	one sibling (brother) with similar phenotype (individual 41); parental couple experienced three miscarriages	parental couple experienced three miscarriages	-	-	one sibling (individual 44) with similar phenotype	-

Abbreviations are as follows: M: male; F: female; NA: Not Available; +: clinical trait present; -: clinical trait absent; PFO: Patent Foramen Ovale; ADHD: Attention Deficit Hyperactivity Disorder; IQ: Intelligence Quotient; ASD: Autism Spectrum Disorder; ECG: Electrocardiogram; Echo: Echocardiography; EEG: Electroencephalogram, MRI: Magnetic Resonance Imaging; G-tube: gastrostomy tube, PDA: Patent Ductus Arteriosus.

Table S2. Pathogenicity prediction of three novel *GNB5* variants

	GNB5 variants		
Prediction tool	p.(Ser81Trp)	p.(Gly215Glu)	p.(Leu59Pro)
Mutation Taster	Disease causing (0.99)	Disease causing (0.99)	Disease causing (0.99)
PolyPhen-2	Probably Damaging (1)	Probably Damaging (1)	Probably Damaging (1)
SIFT	Deleterious (0.00)	Deleterious (0.00)	Deleterious (0.00)
Provean	Deleterious (-5.274)	Deleterious (-7.426)	Deleterious (-6.156)
UMD-predictor	Pathogenic (100)	Pathogenic (99)	Pathogenic (87)
FATHMM	Damaging (-6.79)	Damaging (-6.92)	-
NNSplice	-	Donor increased	-
NetGene2	-	Activation of donor splice site	-
Splicing Finder	Activation of an exonic cryptic donor site, creation of an exonic ESS site	Creation of an exonic ESS site, alteration of an exonic ESE site	-
CADD	31	29.1	5.091

Supp. Table S3. Description of the mouse samples used for transcriptome

Sample name	Gnb5 genotype	Tissue
KO_At_M1	-/-	Atria
HET_At_M2	+/-	Atria
KO_At_M3	-/-	Atria
HET_At_M4	+/-	Atria
KO_At_M5	-/-	Atria
HET_At_M6	+/-	Atria
WT_At_M7	+/+	Atria
WT_At_M8	+/+	Atria
HET_At_M9	+/-	Atria
KO_At_M10	-/-	Atria
HET_At_M11	+/-	Atria
KO_At_M12	+/-	Atria
HET_At_M13	+/-	Atria
KO_At_M14	-/-	Atria
WT_At_M15	+/+	Atria
WT_At_M16	+/+	Atria
WT_At_M17	+/+	Atria
WT_At_M18	+/+	Atria
KO_At_M1	-/-	Ventricles
HET_At_M2	+/-	Ventricles
KO_At_M3	-/-	Ventricles
HET_At_M4	+/-	Ventricles
KO_At_M5	-/-	Ventricles
HET_At_M6	+/-	Ventricles
WT_At_M7	+/+	Ventricles
WT_At_M8	+/+	Ventricles
HET_At_M9	+/-	Ventricles
KO_At_M10	-/-	Ventricles
HET_At_M11	+/-	Ventricles
KO_At_M12	+/-	Ventricles
HET_At_M13	+/-	Ventricles
KO_At_M14	-/-	Ventricles
WT_At_M15	+/+	Ventricles
WT_At_M16	+/+	Ventricles
WT_At_M17	+/+	Ventricles
WT_At_M18	+/+	Ventricles
KO_At_M1	-/-	Cerebellum
KO_At_M3	-/-	Cerebellum
KO_At_M5	-/-	Cerebellum

WT_At_M7	+/+	Cerebellum
WT_At_M8	+/+	Cerebellum
KO_At_M10	-/-	Cerebellum
KO_At_M12	+/-	Cerebellum
KO_At_M14	-/-	Cerebellum
WT_At_M15	+/+	Cerebellum
WT_At_M16	+/+	Cerebellum
WT_At_M17	+/+	Cerebellum
WT_At_M18	+/+	Cerebellum
KO_At_M1	-/-	Hippocampus
KO_At_M3	-/-	Hippocampus
KO_At_M5	-/-	Hippocampus
WT_At_M7	+/+	Hippocampus
WT_At_M8	+/+	Hippocampus
KO_At_M10	-/-	Hippocampus
KO_At_M12	+/-	Hippocampus
KO_At_M14	-/-	Hippocampus
WT_At_M15	+/+	Hippocampus
WT_At_M16	+/+	Hippocampus
WT_At_M17	+/+	Hippocampus
WT_At_M18	+/+	Hippocampus
KO_At_M1	-/-	Cerebral cortex
KO_At_M3	-/-	Cerebral cortex
KO_At_M5	-/-	Cerebral cortex
WT_At_M7	+/+	Cerebral cortex
WT_At_M8	+/+	Cerebral cortex
KO_At_M10	-/-	Cerebral cortex
KO_At_M12	+/-	Cerebral cortex
KO_At_M14	-/-	Cerebral cortex
WT_At_M15	+/+	Cerebral cortex
WT_At_M16	+/+	Cerebral cortex
WT_At_M17	+/+	Cerebral cortex
WT_At_M18	+/+	Cerebral cortex

Supp Table S4. List of Differentially Expressed genes at FDR 5% in *Gnb5-/-* mice - atria

Ensembl ID	Gene Symbol	Gene name	baseMean	log2FoldChange	lfcSE	stat	pvalue	padj
ENSMUSG00000032192	Gnb5	guanine nucleotide binding protein (G protein), beta 5	588.3942165	-3.98100257	0.125384112	-31.7504546	3.13E-221	5.47E-217
ENSMUSG00000033419	Snap91	synaptosomal-associated protein 91	593.4008865	-2.204605213	0.126013386	-17.49500813	1.56E-68	1.37E-64
ENSMUSG00000048758	Rpl29	ribosomal protein L29	199.1838179	-3.250034673	0.22769114	-14.2738741	3.18E-46	1.39E-42
ENSMUSG00000032224	Fam81a	family with sequence similarity 81, member A	292.8574685	2.572498612	0.253223883	10.15898889	3.02E-24	1.06E-20
ENSMUSG00000032179	Bmp5	bone morphogenetic protein 5	205.0671832	1.328934231	0.136737848	9.718847054	2.51E-22	7.30E-19
ENSMUSG00000033590	Myo5c	myosin VC	163.7773657	-1.49164629	0.195137706	-7.644070025	2.10E-14	5.26E-11
ENSMUSG00000023979	Guca1b	guanylate cyclase activator 1B	58.67765502	1.533209378	0.212719605	7.207654307	5.69E-13	1.24E-09
ENSMUSG00000039716	Dock3	dedicator of cyto-kinesis 3	188.3304401	-1.168897128	0.168267704	-6.94665168	3.74E-12	7.27E-09
ENSMUSG00000032184	Lysmd2	LysM, putative peptidoglycan-binding, domain containing 2	586.6508467	0.667351463	0.097089163	6.873593728	6.26E-12	1.09E-08
ENSMUSG00000023249	Parp3	poly (ADP-ribose) polymerase family, member 3	1837.440683	-0.66820649	0.10214733	-6.541595295	6.09E-11	9.68E-08
ENSMUSG00000032556	Bfsp2	beaded filament structural protein 2, phakinin	57.65614782	-2.234680029	0.347888403	-6.423554248	1.33E-10	1.94E-07
ENSMUSG00000042073	Abhd14b	abhydrolase domain containing 14b	580.8699606	-0.801977921	0.127326525	-6.298592679	3.00E-10	4.04E-07
ENSMUSG00000032579	Hemk1	HemK methyltransferase family member 1	597.5715156	0.879217891	0.141144598	6.229199738	4.69E-10	5.56E-07
ENSMUSG00000032872	Cyb5r4	cytochrome b5 reductase 4	1656.745906	-0.509974877	0.081905477	-6.226383107	4.77E-10	5.56E-07
ENSMUSG00000066456	Hmgn3	high mobility group nucleosomal binding domain 3	282.7147136	-0.762798777	0.13316698	-5.728137557	1.02E-08	1.11E-05
ENSMUSG00000031849	Comp	cartilage oligomeric matrix protein	1890.434541	0.863942512	0.156893591	5.506550687	3.66E-08	3.76E-05
ENSMUSG00000043587	Pxylp1	2-phosphoxylose phosphatase 1	553.184341	-1.06209914	0.195018224	-5.446153283	5.15E-08	5.00E-05
ENSMUSG00000032563	Mrpl3	mitochondrial ribosomal protein L3	1799.161022	-0.582173662	0.107712274	-5.404896214	6.48E-08	5.97E-05

ENSMUSG00000010057	Nprl2	NPR2 like, GATOR1 complex subunit	671.0578114	0.433311959	0.080955871	5.352446372	8.68E-08	7.23E-05
ENSMUSG00000044938	Klhl31	kelch-like 31	2434.146223	-0.686725512	0.129907316	-5.286272829	1.25E-07	9.92E-05
ENSMUSG00000029683	Lmod2	leiomodin 2 (cardiac)	11173.11087	-0.54016025	0.102389096	-5.275564196	1.32E-07	1.01E-04
ENSMUSG00000015354	Pcolce2	procollagen C-endopeptidase enhancer 2	1381.444784	0.377432306	0.072416176	5.21198893	1.87E-07	1.36E-04
ENSMUSG00000113637	Gm7049	predicted gene 7049	15.78647891	2.22274028	0.431035014	5.156751094	2.51E-07	1.76E-04
ENSMUSG00000039313	Minar1	membrane integral NOTCH2 associated receptor 1	31.93558633	1.962031653	0.383199078	5.120136678	3.05E-07	2.05E-04
ENSMUSG00000078137	Ankrd63	ankyrin repeat domain 63	231.3397505	0.742695994	0.148629054	4.99697718	5.82E-07	3.77E-04
ENSMUSG00000063564	Col23a1	collagen, type XXIII, alpha 1	520.8704226	0.624586575	0.126165837	4.950520603	7.40E-07	4.62E-04
ENSMUSG00000038156	Spon1	spondin 1, (f-spondin) extracellular matrix protein	3543.885101	0.371254886	0.075961149	4.887431184	1.02E-06	6.16E-04
ENSMUSG00000032058	Ppp2r1b	protein phosphatase 2, regulatory subunit A, beta	374.2643884	0.599231525	0.123151982	4.86578874	1.14E-06	6.65E-04
ENSMUSG00000041112	Elmo1	engulfment and cell motility 1	700.2407007	0.415448791	0.085517427	4.858059972	1.19E-06	6.69E-04
ENSMUSG00000091537	Tma7	translational machinery associated 7	483.7361455	-0.685576063	0.143465638	-4.77867782	1.76E-06	9.64E-04
ENSMUSG00000022206	Npr3	natriuretic peptide receptor 3	11899.26299	0.399734149	0.083783676	4.771026642	1.83E-06	9.71E-04
ENSMUSG00000032370	Lactb	lactamase, beta	346.157966	-0.422281978	0.09066889	-4.65740759	3.20E-06	1.65E-03
ENSMUSG00000043719	Col6a6	collagen, type VI, alpha 6	1411.565721	0.828808973	0.179039549	4.629194943	3.67E-06	1.83E-03
ENSMUSG00000026638	Irf6	interferon regulatory factor 6	354.4281902	0.612952619	0.132744643	4.61753185	3.88E-06	1.89E-03
ENSMUSG00000033453	Adamts15	a disintegrin-like and metallopeptidase (reprolysin type) with thrombospondin type 1 motif, 15	1535.603828	0.567295151	0.123255032	4.60261251	4.17E-06	1.97E-03
ENSMUSG00000032498	Mlh1	mutL homolog 1	433.7803123	-0.38578188	0.084367758	-4.572622185	4.82E-06	2.22E-03
ENSMUSG00000028476	Reck	reversion-inducing-cysteine-rich protein with kazal motifs	744.0022468	0.325339922	0.071357088	4.559321694	5.13E-06	2.30E-03

ENSMUSG00000021596	Mctp1	multiple C2 domains, transmembrane 1	179.9515026	0.502011368	0.111477268	4.5032622	6.69E-06	2.93E-03
ENSMUSG00000090626	Tex9	testis expressed gene 9	278.1642117	-0.734429387	0.163584055	-4.489614741	7.14E-06	3.04E-03
ENSMUSG00000032567	Aste1	asteroid homolog 1	192.4949534	-0.666334448	0.14938301	-4.460577198	8.17E-06	3.40E-03
ENSMUSG00000032431	Crtap	cartilage associated protein	1361.165212	0.364784746	0.082147038	4.440631755	8.97E-06	3.65E-03
ENSMUSG00000049624	Slc17a5	solute carrier family 17 (anion/sugar transporter), member 5	349.6898644	0.434839012	0.09846974	4.415965889	1.01E-05	4.00E-03
ENSMUSG00000053040	Aph1c	aph1 homolog C, gamma secretase subunit	68.79153406	-0.82659287	0.187704162	-4.403700279	1.06E-05	4.14E-03
ENSMUSG00000024806	Mlana	melan-A	1627.583495	0.52838317	0.122891802	4.299580298	1.71E-05	6.51E-03
ENSMUSG00000028132	Tmem56	transmembrane protein 56	245.5306833	-0.815298268	0.191265107	-4.262660763	2.02E-05	7.52E-03
ENSMUSG00000090215	Trim34b	tripartite motif-containing 34B	11.6973086	-2.582632252	0.607008212	-4.254690792	2.09E-05	7.63E-03
ENSMUSG00000028396	2310002L09Rik	RIKEN cDNA 2310002L09 gene	344.3905263	-0.626139305	0.147377011	-4.248554772	2.15E-05	7.68E-03
ENSMUSG00000037736	Limch1	LIM and calponin homology domains 1	5228.144385	-0.312723246	0.073777902	-4.238711583	2.25E-05	7.86E-03
ENSMUSG00000031980	Agt	angiotensinogen (serpin peptidase inhibitor, clade A, member 8)	524.2715869	-0.609713334	0.144159559	-4.229433963	2.34E-05	7.97E-03
ENSMUSG00000040848	Sft2d2	SFT2 domain containing 2	2529.424876	0.234751352	0.055536798	4.226951515	2.37E-05	7.97E-03
ENSMUSG00000068227	Il2rb	interleukin 2 receptor, beta chain	82.42680251	0.659994674	0.156469105	4.218051048	2.46E-05	8.13E-03
ENSMUSG00000074059	Fbxw18	F-box and WD-40 domain protein 18	62.68513319	3.258911738	0.774283545	4.208938393	2.57E-05	8.31E-03
ENSMUSG00000078307	AI593442	expressed sequence AI593442	39.35947148	-1.838584277	0.438071347	-4.196997335	2.70E-05	8.60E-03
ENSMUSG00000019102	Aldh3a1	aldehyde dehydrogenase family 3, subfamily A1	66.67934433	1.063561484	0.253673991	4.192631185	2.76E-05	8.61E-03
ENSMUSG00000100782	Gm28231	predicted gene 28231	22.5434237	1.248506266	0.301217727	4.144863178	3.40E-05	1.04E-02
ENSMUSG00000051855	Mest	mesoderm specific transcript	455.3390815	0.528823455	0.129171073	4.093977412	4.24E-05	1.28E-02
ENSMUSG00000045594	Glb1	galactosidase, beta 1	946.3638084	-0.476991352	0.11686762	-4.081467171	4.48E-05	1.31E-02

ENSMUSG00000053093	Myh7	myosin, heavy polypeptide 7, cardiac muscle, beta	251.3134444	-0.866946809	0.212498462	-4.079779212	4.51E-05	1.31E-02
ENSMUSG00000006941	Eif1b	eukaryotic translation initiation factor 1B	1363.496793	-0.405756831	0.100156813	-4.051215455	5.10E-05	1.40E-02
ENSMUSG00000032342	Mto1	mitochondrial tRNA translation optimization 1	741.1169021	-0.255310451	0.063047704	-4.04948058	5.13E-05	1.40E-02
ENSMUSG00000111278	Gm32743	predicted gene 32743	25.8092539	-1.363865868	0.336722402	-4.050416183	5.11E-05	1.40E-02
ENSMUSG00000050640	Tmem150c	transmembrane protein 150C	40.08771757	-1.128379631	0.279418769	-4.038310072	5.38E-05	1.45E-02
ENSMUSG00000056880	Gadl1	glutamate decarboxylase-like 1	41.12226685	-1.172852268	0.291809453	-4.019240144	5.84E-05	1.55E-02
ENSMUSG00000006587	Snai3	snail family zinc finger 3	38.46789747	-1.556170083	0.3914073	-3.975833063	7.01E-05	1.83E-02
ENSMUSG00000032860	P2ry2	purinergic receptor P2Y, G-protein coupled 2	427.8475396	-0.417060355	0.105028679	-3.970918781	7.16E-05	1.84E-02
ENSMUSG00000021622	Ckmt2	creatine kinase, mitochondrial 2	30570.15221	-0.358321877	0.09037697	-3.964747643	7.35E-05	1.86E-02
ENSMUSG00000104344	Gm38077	predicted gene 38077	110.0410307	-0.694683493	0.17726808	-3.918830127	8.90E-05	2.22E-02
ENSMUSG00000032181	Scg3	secretogranin III	41.68914485	1.290206572	0.330188439	3.907485597	9.33E-05	2.30E-02
ENSMUSG00000020253	Ppm1m	protein phosphatase 1M	612.6332904	0.364501693	0.094443251	3.859478465	1.14E-04	2.76E-02
ENSMUSG00000097088	Gm26615	predicted gene 26615	43.50706638	0.836267574	0.216957467	3.854523129	1.16E-04	2.78E-02
ENSMUSG00000010064	Slc38a3	solute carrier family 38, member 3	1610.204165	-0.651101006	0.169436841	-3.842735747	1.22E-04	2.80E-02
ENSMUSG00000031709	Tbc1d9	TBC1 domain family, member 9	542.8723027	0.411804108	0.107166775	3.842647212	1.22E-04	2.80E-02
ENSMUSG00000034533	Scn10a	sodium channel, voltage-gated, type X, alpha	703.8602467	0.70040763	0.183064182	3.826022233	1.30E-04	2.88E-02
ENSMUSG00000040875	Osbpl10	oxysterol binding protein-like 10	79.84746132	-0.766643335	0.200280981	-3.82783892	1.29E-04	2.88E-02
ENSMUSG00000101257	2310015K22Rik	RIKEN cDNA 2310015K22 gene	12.05415905	-1.633324502	0.426600907	-3.828694395	1.29E-04	2.88E-02
ENSMUSG00000019817	Plagl1	pleiomorphic adenoma gene-like 1	2018.952602	0.453482958	0.119673938	3.789320943	1.51E-04	3.29E-02
ENSMUSG00000019890	Nts	neurotensin	85.16574876	-0.855082132	0.225793704	-3.787006094	1.52E-04	3.29E-02
ENSMUSG00000030515	Tarsl2	threonyl-tRNA synthetase-like 2	1170.685678	-0.243655404	0.064511345	-3.776938834	1.59E-04	3.39E-02
ENSMUSG00000006611	Hfe	hemochromatosis	890.846647	0.435320115	0.11562911	3.764796886	1.67E-04	3.51E-02

ENSMUSG00000045967	Gpr158	G protein-coupled receptor 158	373.298715	-0.686733554	0.18272265	-3.75833841	1.71E-04	3.52E-02
ENSMUSG00000047686	Rtl3	retrotransposon Gag like 3	266.2423324	0.760639578	0.20238611	3.758358611	1.71E-04	3.52E-02
ENSMUSG00000035606	Ky	kyphoscoliosis peptidase	125.6420813	-0.986271443	0.263125553	-3.748292139	1.78E-04	3.61E-02
ENSMUSG00000074207	Adh1	alcohol dehydrogenase 1 (class I)	1168.841709	0.378713887	0.101093013	3.746192504	1.80E-04	3.61E-02
ENSMUSG00000000184	Ccnd2	cyclin D2	7244.566123	0.321075122	0.085868752	3.739138085	1.85E-04	3.67E-02
ENSMUSG00000021557	Agtpbp1	ATP/GTP binding protein 1	3779.137522	-0.28831125	0.077403559	-3.724780271	1.95E-04	3.84E-02
ENSMUSG00000021557	A230056J06Rik	RIKEN cDNA A230056J06 gene	3779.137522	-0.28831125	0.077403559	-3.724780271	1.95E-04	3.84E-02
ENSMUSG00000043795	Prr33	proline rich 33	112.7136835	-0.776040696	0.208764671	-3.717298975	2.01E-04	3.91E-02
ENSMUSG00000026604	Ptpn14	protein tyrosine phosphatase, non-receptor type 14	3680.35312	0.257384789	0.069385735	3.70947703	2.08E-04	3.99E-02
ENSMUSG00000029359	Tesc	tescalcin	181.5637758	-0.947384507	0.256043393	-3.700093552	2.16E-04	4.05E-02
ENSMUSG00000031637	Lrp2bp	Lrp2 binding protein	140.0742862	-0.59046599	0.159527357	-3.701346278	2.14E-04	4.05E-02
ENSMUSG00000021957	Tkt	transketolase	1797.82233	0.732078661	0.198124968	3.695034851	2.20E-04	4.06E-02
ENSMUSG00000061780	Cfd	complement factor D (adipsin)	904.2314372	2.994362377	0.810510725	3.694414255	2.20E-04	4.06E-02
ENSMUSG00000037887	Dusp8	dual specificity phosphatase 8	774.5163123	-0.572804466	0.155345266	-3.68729915	2.27E-04	4.13E-02
ENSMUSG00000091345	Col6a5	collagen, type VI, alpha 5	326.9932512	2.283384011	0.622617345	3.667395437	2.45E-04	4.42E-02
ENSMUSG00000060639	Hist1h4i	histone cluster 1, H4i	144.8255591	-0.691858999	0.188964427	-3.661318753	2.51E-04	4.48E-02
ENSMUSG00000064371	mt-Tt	mitochondrially encoded tRNA threonine	67.46562005	-0.989792385	0.27087529	-3.654051962	2.58E-04	4.56E-02
ENSMUSG00000034485	Uaca	uveal autoantigen with coiled-coil domains and ankyrin repeats	1707.508249	-0.254012029	0.069644209	-3.647281416	2.65E-04	4.63E-02
ENSMUSG00000059742	Kcnh7	potassium voltage-gated channel, subfamily H (eag-related), member 7	286.2843348	0.587508859	0.161412283	3.639802667	2.73E-04	4.72E-02

Supp Table S5. List of Differentially Expressed genes at FDR 5% in *Gnb5-/-* mice - ventricle

Ensembl ID	Gene Symbol	Gene name	baseMean	log2FoldChange	lfcSE	stat	pvalue	padj
ENSMUSG00000032192	Gnb5	guanine nucleotide binding protein (G protein), beta 5	313.5699776	-4.21550606	0.154170849	-27.34308137	1.31E-164	2.19E-160
ENSMUSG00000033419	Snap91	synaptosomal-associated protein 91	130.8116563	-2.993668387	0.230324744	-12.9975978	1.26E-38	1.06E-34
ENSMUSG00000048758	Rpl29	ribosomal protein L29	148.0555782	-2.768271599	0.258451771	-10.71097942	9.04E-27	5.05E-23
ENSMUSG00000032224	Fam81a	family with sequence similarity 81, member A	535.3572241	1.400193385	0.158370442	8.841254493	9.46E-19	3.96E-15
ENSMUSG00000032579	Hemk1	HemK methyltransferase family member 1	699.5483486	1.255117802	0.16788463	7.476073332	7.66E-14	2.57E-10
ENSMUSG00000113637	Gm7049	predicted gene 7049	16.81236237	2.249097307	0.306354635	7.341482876	2.11E-13	5.90E-10
ENSMUSG00000111765	Gm10635	predicted gene 10635	108.9820339	-1.98648208	0.288335345	-6.889485152	5.60E-12	1.34E-08
ENSMUSG00000066456	Hmgn3	high mobility group nucleosomal binding domain 3	80.22459437	-1.098602846	0.160705843	-6.836110138	8.14E-12	1.70E-08
ENSMUSG00000023249	Parp3	poly (ADP-ribose) polymerase family, member 3	1606.053611	-0.717032187	0.109004266	-6.578019501	4.77E-11	8.88E-08
ENSMUSG00000010057	Nprl2	NPR2 like, GATOR1 complex subunit	491.5575602	0.487515133	0.082544171	5.906112138	3.50E-09	5.34E-06
ENSMUSG00000100782	Gm28231	predicted gene 28231	48.63773997	1.405293992	0.238858846	5.883365906	4.02E-09	5.61E-06
ENSMUSG00000020902	Ntn1	netrin 1	2523.450781	0.593131025	0.102551465	5.783740167	7.31E-09	9.42E-06
ENSMUSG00000019817	Plagl1	pleiomorphic adenoma gene-like 1	76.29245071	0.819492865	0.146652951	5.587973925	2.30E-08	2.57E-05
ENSMUSG00000042073	Abhd14b	abhydrolase domain containing 14b	312.5043269	-0.742734019	0.132845695	-5.590952862	2.26E-08	2.57E-05
ENSMUSG00000038801	Scgb1c1	secretoglobin, family 1C, member 1	193.8961	1.137948154	0.205183249	5.546009029	2.92E-08	2.88E-05
ENSMUSG00000097099	Gm9917	predicted gene 9917	111.2305592	-0.971774724	0.179085224	-5.426325529	5.75E-08	5.35E-05
ENSMUSG00000107653	Gm31520	sterol-C5-desaturase pseudogene	38.9097343	1.461818509	0.276075681	5.294991955	1.19E-07	1.05E-04
ENSMUSG00000032419	Tbx18	T-box18	104.4903783	-0.654370039	0.125020141	-5.234116949	1.66E-07	1.32E-04
ENSMUSG00000020253	Ppm1m	protein phosphatase 1M	224.4754793	0.629411893	0.120929771	5.204772037	1.94E-07	1.48E-04
ENSMUSG00000032563	Mrpl3	mitochondrial ribosomal protein L3	1783.956096	-0.436886258	0.085393676	-5.116143011	3.12E-07	2.27E-04

ENSMUSG00000032261	Sh3bgrl2	SH3 domain binding glutamic acid-rich protein like 2	105.6921769	-0.653725	0.128760505	-5.077061488	3.83E-07	2.68E-04
ENSMUSG00000023979	Guca1b	guanylate cyclase activator 1B	25.91071064	1.315395153	0.259962618	5.059939622	4.19E-07	2.81E-04
ENSMUSG00000091345	Col6a5	collagen, type VI, alpha 5	29.84898234	1.907639946	0.3775694	5.052422015	4.36E-07	2.81E-04
ENSMUSG00000032359	Ctsh	cathepsin H	710.7231171	-0.623022117	0.125532066	-4.963051582	6.94E-07	4.15E-04
ENSMUSG00000022219	Cideb	cell death-inducing DNA fragmentation factor, alpha subunit-like effector B	74.13239706	0.858582759	0.175534791	4.891239831	1.00E-06	5.79E-04
ENSMUSG00000010064	Slc38a3	solute carrier family 38, member 3	2341.478262	-0.809203125	0.167037335	-4.844444656	1.27E-06	7.09E-04
ENSMUSG00000032259	Drd2	dopamine receptor D2	85.88598789	0.839883098	0.174723741	4.8069203	1.53E-06	8.28E-04
ENSMUSG00000032537	Ephb1	Eph receptor B1	585.1805641	2.398021253	0.521600636	4.597427777	4.28E-06	2.24E-03
ENSMUSG00000104344	Gm38077	predicted gene 38077	168.5348807	-0.988910029	0.216709138	-4.563305623	5.04E-06	2.56E-03
ENSMUSG00000030990	Pgap2	post-GPI attachment to proteins 2	688.9552575	0.468413015	0.104101368	4.499585592	6.81E-06	3.36E-03
ENSMUSG00000062270	Morf4l1	mortality factor 4 like 1	1196.914037	-0.415696122	0.093949616	-4.424670816	9.66E-06	4.62E-03
ENSMUSG00000062270	Morf4l1b	mortality factor 4 like 1B	1196.914037	-0.415696122	0.093949616	-4.424670816	9.66E-06	4.62E-03
ENSMUSG00000032872	Cyb5r4	cytochrome b5 reductase 4	1012.878713	-0.551302186	0.125317439	-4.399245566	1.09E-05	4.92E-03
ENSMUSG00000079355	Ackr4	atypical chemokine receptor 4	94.67720248	-0.760983138	0.172765696	-4.404712012	1.06E-05	4.92E-03
ENSMUSG00000020431	Adcy1	adenylate cyclase 1	97.1204426	0.866213531	0.197445358	4.38710508	1.15E-05	4.93E-03
ENSMUSG00000023439	Gnb3	guanine nucleotide binding protein (G protein), beta 3	256.1773659	0.925120884	0.249262978	3.711425138	2.06E-04	5.21E-03
ENSMUSG00000043587	Pxylp1	2-phosphoxylose phosphatase 1	148.4011593	-0.941500636	0.215647645	-4.365921245	1.27E-05	5.21E-03
ENSMUSG00000100410	2310020H05Rik	RIKEN cDNA 2310020H05 gene	82.70867895	-0.95306705	0.218380045	-4.364258874	1.28E-05	5.21E-03
ENSMUSG00000023262	Acy1	aminoacylase 1	275.312161	0.52627658	0.12086618	4.354208773	1.34E-05	5.28E-03
ENSMUSG00000032420	Nt5e	5' nucleotidase, ecto	531.2462273	0.575371191	0.132243883	4.35083406	1.36E-05	5.28E-03
ENSMUSG00000032342	Mto1	mitochondrial tRNA translation optimization 1	628.7435289	-0.35212118	0.081316245	-4.330268576	1.49E-05	5.54E-03

ENSMUSG00000036006	Ripor2	RHO family interacting cell polarization regulator 2	280.1521625	0.766998221	0.178845813	4.288600385	1.80E-05	6.55E-03
ENSMUSG00000032567	Aste1	asteroid homolog 1	136.4175984	-0.546329802	0.129600006	-4.215507543	2.49E-05	8.88E-03
ENSMUSG00000007033	Hspa1l	heat shock protein 1-like	313.1401892	-0.868331662	0.207647674	-4.181754823	2.89E-05	1.01E-02
ENSMUSG00000062991	Nrg1	neuregulin 1	36.63309961	1.167251298	0.281216976	4.150714202	3.31E-05	1.13E-02
ENSMUSG00000057802	Gm10030	predicted gene 10030	23.54320155	1.146733244	0.277719383	4.129107706	3.64E-05	1.22E-02
ENSMUSG00000032500	Dclk3	doublecortin-like kinase 3	16.62670461	1.320424335	0.32048356	4.120100055	3.79E-05	1.24E-02
ENSMUSG00000030592	Ryr1	ryanodine receptor 1, skeletal muscle	18.10439933	-1.213191335	0.295663089	-4.103289788	4.07E-05	1.31E-02
ENSMUSG00000034533	Scn10a	sodium channel, voltage-gated, type X, alpha	113.9830881	0.858252285	0.213468675	4.020506927	5.81E-05	1.84E-02
ENSMUSG00000031066	Usp11	ubiquitin specific peptidase 11	212.3557436	0.528925104	0.131795322	4.013231248	5.99E-05	1.86E-02
ENSMUSG00000071708	Sms	spermine synthase	825.2887178	-0.362951165	0.090642781	-4.004192737	6.22E-05	1.86E-02
ENSMUSG00000113321	Gm8775	predicted gene 8775	29.48173879	1.076447965	0.268553676	4.008315883	6.12E-05	1.86E-02
ENSMUSG00000073485	H3f3aos	H3 histone, family 3A	31.32162143	-0.946720716	0.236742268	-3.998950945	6.36E-05	1.87E-02
ENSMUSG00000026414	Tnnt2	troponin T2, cardiac	217.8533484	0.619746386	0.156123287	3.969596064	7.20E-05	2.08E-02
ENSMUSG00000031637	Lrp2bp	Lrp2 binding protein	63.26457812	-1.031672567	0.264188857	-3.90505709	9.42E-05	2.68E-02
ENSMUSG00000045594	Glb1	galactosidase, beta 1	384.1377132	-0.474925266	0.123283793	-3.852292773	1.17E-04	3.27E-02
ENSMUSG00000010051	Hyal1	hyaluronoglucosaminidase 1	122.0241169	0.64246724	0.167341124	3.839266904	1.23E-04	3.39E-02
ENSMUSG00000020534	Shmt1	serine hydroxymethyltransferase 1 (soluble)	289.1479786	0.655279666	0.171422662	3.822596498	1.32E-04	3.57E-02
ENSMUSG00000060187	Lrrc10	leucine rich repeat containing 10	2494.259676	0.407948338	0.111196436	3.668717757	2.44E-04	3.91E-02
ENSMUSG00000027313	Chac1	ChaC, cation transport regulator 1	44.4569393	-1.241707583	0.328017443	-3.785492538	1.53E-04	4.08E-02
ENSMUSG00000024145	Pigf	phosphatidylinositol glycan anchor biosynthesis, class F	53.55766273	0.70037248	0.186231311	3.760766521	1.69E-04	4.37E-02
ENSMUSG00000052485	Tmem171	transmembrane protein 171	169.2165357	0.76792289	0.204074434	3.762954896	1.68E-04	4.37E-02
ENSMUSG00000031461	Myom2	myomesin 2	39422.33605	0.551200865	0.150744712	3.65651875	2.56E-04	4.71E-02

Supp. Table S6. List of Biological Processes (BP) at FDR 5% - atria

GO ID	Description	BgRatio	pvalue	p.adjust	qvalue	geneID	Count
GO:0046184	aldehyde biosynthetic process	18/21092	5.11E-05	7.82E-02	0.075133254	Bmp5/Adh1/Tkt	3
GO:0006081	cellular aldehyde metabolic process	68/21092	1.72E-04	1.32E-01	0.126431472	Bmp5/Aldh3a1/Adh1/Tkt	4
GO:0032892	positive regulation of organic acid transport	47/21092	9.32E-04	4.20E-01	0.403462799	Agt/P2ry2/Slc38a3	3
GO:0015802	basic amino acid transport	14/21092	1.45E-03	4.20E-01	0.403462799	Agt/Slc38a3	2
GO:1903793	positive regulation of anion transport	63/21092	2.18E-03	4.20E-01	0.403462799	Agt/P2ry2/Slc38a3	3
GO:0006883	cellular sodium ion homeostasis	19/21092	2.69E-03	4.20E-01	0.403462799	Agt/Tesc	2
GO:0008015	blood circulation	466/21092	2.94E-03	4.20E-01	0.403462799	Comp/Npr3/Agt/Myh7/P2ry2/Scn10a/Nts	7
GO:0003013	circulatory system process	475/21092	3.27E-03	4.20E-01	0.403462799	Comp/Npr3/Agt/Myh7/P2ry2/Scn10a/Nts	7
GO:0032890	regulation of organic acid transport	77/21092	3.85E-03	4.20E-01	0.403462799	Agt/P2ry2/Slc38a3	3
GO:0043687	post-translational protein modification	23/21092	3.93E-03	4.20E-01	0.403462799	Crtap/Agtpbp1	2
GO:0014829	vascular smooth muscle contraction	25/21092	4.64E-03	4.20E-01	0.403462799	Comp/Agt	2
GO:0050880	regulation of blood vessel size	168/21092	4.97E-03	4.20E-01	0.403462799	Comp/Agt/P2ry2/Nts	4
GO:0035150	regulation of tube size	169/21092	5.07E-03	4.20E-01	0.403462799	Comp/Agt/P2ry2/Nts	4
GO:0032526	response to retinoic acid	86/21092	5.25E-03	4.20E-01	0.403462799	Mest/Adh1/Tesc	3
GO:0051957	positive regulation of amino acid transport	28/21092	5.80E-03	4.20E-01	0.403462799	Agt/Slc38a3	2
GO:0006936	muscle contraction	281/21092	5.87E-03	4.20E-01	0.403462799	Comp/Lmod2/Agt/Myh7/Scn10a	5
GO:0002027	regulation of heart rate	91/21092	6.14E-03	4.20E-01	0.403462799	Agt/Myh7/Scn10a	3
GO:0045652	regulation of megakaryocyte differentiation	30/21092	6.64E-03	4.20E-01	0.403462799	Tesc/Hist1h4i	2
GO:1900274	regulation of phospholipase C activity	30/21092	6.64E-03	4.20E-01	0.403462799	Snap91/Agt	2

GO:0003018	vascular process in circulatory system	193/21092	8.05E-03	4.20E-01	0.403462799	Comp/Agt/P2ry2/Nts	4
GO:0006739	NADP metabolic process	36/21092	9.46E-03	4.20E-01	0.403462799	Cyb5r4/Tkt	2
GO:0042445	hormone metabolic process	207/21092	1.02E-02	4.20E-01	0.403462799	Bmp5/Agt/Hfe/Adh1	4
GO:0031279	regulation of cyclase activity	41/21092	1.22E-02	4.20E-01	0.403462799	Guca1b/Npr3	2
GO:0044070	regulation of anion transport	117/21092	1.22E-02	4.20E-01	0.403462799	Agt/P2ry2/Slc38a3	3
GO:0051339	regulation of lyase activity	43/21092	1.33E-02	4.20E-01	0.403462799	Guca1b/Npr3	2
GO:0032007	negative regulation of TOR signaling	45/21092	1.45E-02	4.20E-01	0.403462799	Nprl2/Minar1	2
GO:0010862	positive regulation of pathway-restricted SMAD protein phosphorylation	49/21092	1.71E-02	4.20E-01	0.403462799	Bmp5/Hfe	2
GO:0050905	neuromuscular process	133/21092	1.72E-02	4.20E-01	0.403462799	Comp/Tmem150c/Agtpbp1	3
GO:0032941	secretion by tissue	50/21092	1.77E-02	4.20E-01	0.403462799	Npr3/P2ry2	2
GO:0051955	regulation of amino acid transport	50/21092	1.77E-02	4.20E-01	0.403462799	Agt/Slc38a3	2
GO:0055078	sodium ion homeostasis	50/21092	1.77E-02	4.20E-01	0.403462799	Agt/Tesc	2
GO:0002064	epithelial cell development	245/21092	1.80E-02	4.20E-01	0.403462799	Bmp5/Bfsp2/Col23a1/Agt	4
GO:0030219	megakaryocyte differentiation	51/21092	1.84E-02	4.20E-01	0.403462799	Tesc/Hist1h4i	2
GO:0051954	positive regulation of amine transport	51/21092	1.84E-02	4.20E-01	0.403462799	Agt/Slc38a3	2
GO:0001505	regulation of neurotransmitter levels	376/21092	1.88E-02	4.20E-01	0.403462799	Snap91/Mctp1/Agt/P2ry2/Agtpbp1	5
GO:0030198	extracellular matrix organization	249/21092	1.89E-02	4.20E-01	0.403462799	Comp/Col23a1/Reck/Agt	4
GO:0034308	primary alcohol metabolic process	52/21092	1.91E-02	4.20E-01	0.403462799	Bmp5/Adh1	2
GO:0003012	muscle system process	383/21092	2.02E-02	4.20E-01	0.403462799	Comp/Lmod2/Agt/Myh7/Scn10a	5
GO:0010517	regulation of phospholipase activity	54/21092	2.05E-02	4.20E-01	0.403462799	Snap91/Agt	2
GO:0050879	multicellular organismal movement	54/21092	2.05E-02	4.20E-01	0.403462799	Comp/Myh7	2
GO:0050881	musculoskeletal movement	54/21092	2.05E-02	4.20E-01	0.403462799	Comp/Myh7	2
GO:1905952	regulation of lipid localization	143/21092	2.07E-02	4.20E-01	0.403462799	Agt/Mest/P2ry2	3

GO:0006865	amino acid transport	145/21092	2.15E-02	4.20E-01	0.403462799	Slc17a5/Agt/Slc38a3	3
GO:0061337	cardiac conduction	58/21092	2.34E-02	4.20E-01	0.403462799	Agt/Scn10a	2
GO:0015807	L-amino acid transport	59/21092	2.42E-02	4.20E-01	0.403462799	Agt/Slc38a3	2
GO:0014911	positive regulation of smooth muscle cell migration	60/21092	2.50E-02	4.20E-01	0.403462799	Agt/P2ry2	2
GO:0035296	regulation of tube diameter	156/21092	2.60E-02	4.20E-01	0.403462799	Comp/Agt/P2ry2	3
GO:0097746	regulation of blood vessel diameter	156/21092	2.60E-02	4.20E-01	0.403462799	Comp/Agt/P2ry2	3
GO:2001238	positive regulation of extrinsic apoptotic signaling pathway	62/21092	2.65E-02	4.20E-01	0.403462799	Ppp2r1b/Agt	2
GO:0006801	superoxide metabolic process	63/21092	2.73E-02	4.20E-01	0.403462799	Cyb5r4/Agt	2
GO:0060393	regulation of pathway-restricted SMAD protein phosphorylation	63/21092	2.73E-02	4.20E-01	0.403462799	Bmp5/Hfe	2
GO:1901264	carbohydrate derivative transport	64/21092	2.81E-02	4.20E-01	0.403462799	Slc17a5/P2ry2	2
GO:0060389	pathway-restricted SMAD protein phosphorylation	66/21092	2.98E-02	4.20E-01	0.403462799	Bmp5/Hfe	2
GO:0030509	BMP signaling pathway	165/21092	3.00E-02	4.20E-01	0.403462799	Bmp5/Comp/Hfe	3
GO:0001885	endothelial cell development	67/21092	3.06E-02	4.20E-01	0.403462799	Col23a1/Agt	2
GO:0051604	protein maturation	291/21092	3.12E-02	4.20E-01	0.403462799	Comp/Spon1/Aph1c/Tesc	4
GO:0043062	extracellular structure organization	293/21092	3.19E-02	4.20E-01	0.403462799	Comp/Col23a1/Reck/Agt	4
GO:0046942	carboxylic acid transport	297/21092	3.33E-02	4.20E-01	0.403462799	Slc17a5/Agt/P2ry2/Slc38a3	4
GO:0008016	regulation of heart contraction	172/21092	3.33E-02	4.20E-01	0.403462799	Agt/Myh7/Scn10a	3
GO:0015849	organic acid transport	298/21092	3.36E-02	4.20E-01	0.403462799	Slc17a5/Agt/P2ry2/Slc38a3	4
GO:0042446	hormone biosynthetic process	72/21092	3.49E-02	4.20E-01	0.403462799	Bmp5/Hfe	2
GO:0071772	response to BMP	176/21092	3.53E-02	4.20E-01	0.403462799	Bmp5/Comp/Hfe	3
GO:0071773	cellular response to BMP stimulus	176/21092	3.53E-02	4.20E-01	0.403462799	Bmp5/Comp/Hfe	3
GO:0051145	smooth muscle cell differentiation	73/21092	3.58E-02	4.20E-01	0.403462799	Comp/Agt	2
GO:0060191	regulation of lipase activity	73/21092	3.58E-02	4.20E-01	0.403462799	Snap91/Agt	2
GO:0003333	amino acid transmembrane transport	74/21092	3.67E-02	4.20E-01	0.403462799	Agt/Slc38a3	2

GO:0007219	Notch signaling pathway	181/21092	3.79E-02	4.20E-01	0.403462799	Reck/Aph1c/Cfd	3
GO:0055002	striated muscle cell development	181/21092	3.79E-02	4.20E-01	0.403462799	Comp/Lmod2/Agt	3
GO:0043270	positive regulation of ion transport	310/21092	3.80E-02	4.20E-01	0.403462799	Agt/P2ry2/Slc38a3/Tesc	4
GO:0002181	cytoplasmic translation	76/21092	3.86E-02	4.20E-01	0.403462799	Rpl29/Tma7	2
GO:0034765	regulation of ion transmembrane transport	459/21092	3.96E-02	4.20E-01	0.403462799	Gnb5/Agt/Scn10a/Tesc/Kcnh7	5
GO:0030534	adult behavior	185/21092	4.00E-02	4.20E-01	0.403462799	Adh1/Ccnd2/Agtpbp1	3
GO:0006995	cellular response to nitrogen starvation	10/21092	4.00E-02	4.20E-01	0.403462799	Nprl2	1
GO:0007220	Notch receptor processing	10/21092	4.00E-02	4.20E-01	0.403462799	Aph1c	1
GO:0014883	transition between fast and slow fiber	10/21092	4.00E-02	4.20E-01	0.403462799	Myh7	1
GO:0016264	gap junction assembly	10/21092	4.00E-02	4.20E-01	0.403462799	Agt	1
GO:0030049	muscle filament sliding	10/21092	4.00E-02	4.20E-01	0.403462799	Myh7	1
GO:0030854	positive regulation of granulocyte differentiation	10/21092	4.00E-02	4.20E-01	0.403462799	Tesc	1
GO:0031282	regulation of guanylate cyclase activity	10/21092	4.00E-02	4.20E-01	0.403462799	Guca1b	1
GO:0031943	regulation of glucocorticoid metabolic process	10/21092	4.00E-02	4.20E-01	0.403462799	Bmp5	1
GO:0032342	aldosterone biosynthetic process	10/21092	4.00E-02	4.20E-01	0.403462799	Bmp5	1
GO:0043562	cellular response to nitrogen levels	10/21092	4.00E-02	4.20E-01	0.403462799	Nprl2	1
GO:0048149	behavioral response to ethanol	10/21092	4.00E-02	4.20E-01	0.403462799	Adh1	1
GO:0060710	chorio-allantoic fusion	10/21092	4.00E-02	4.20E-01	0.403462799	Bmp5	1
GO:0070142	synaptic vesicle budding	10/21092	4.00E-02	4.20E-01	0.403462799	Snap91	1
GO:0070257	positive regulation of mucus secretion	10/21092	4.00E-02	4.20E-01	0.403462799	P2ry2	1
GO:0070278	extracellular matrix constituent secretion	10/21092	4.00E-02	4.20E-01	0.403462799	Agt	1
GO:1902023	L-arginine transport	10/21092	4.00E-02	4.20E-01	0.403462799	Agt	1
GO:2000807	regulation of synaptic vesicle clustering	10/21092	4.00E-02	4.20E-01	0.403462799	Snap91	1
GO:0006942	regulation of striated muscle contraction	79/21092	4.14E-02	4.20E-01	0.403462799	Myh7/Scn10a	2
GO:0007269	neurotransmitter secretion	190/21092	4.27E-02	4.20E-01	0.403462799	Snap91/Mctp1/P2ry2	3

GO:0099643	signal release from synapse	190/21092	4.27E-02	4.20E-01	0.403462799	Snap91/Mctp1/P2ry2	3
GO:1901888	regulation of cell junction assembly	81/21092	4.33E-02	4.20E-01	0.403462799	Limch1/Agt	2
GO:0006012	galactose metabolic process	11/21092	4.40E-02	4.20E-01	0.403462799	Glb1	1
GO:0006266	DNA ligation	11/21092	4.40E-02	4.20E-01	0.403462799	Parp3	1
GO:0006705	mineralocorticoid biosynthetic process	11/21092	4.40E-02	4.20E-01	0.403462799	Bmp5	1
GO:0006957	complement activation, alternative pathway	11/21092	4.40E-02	4.20E-01	0.403462799	Cfd	1
GO:0007128	meiotic prophase I	11/21092	4.40E-02	4.20E-01	0.403462799	Mlh1	1
GO:0010872	regulation of cholesterol esterification	11/21092	4.40E-02	4.20E-01	0.403462799	Agt	1
GO:0010958	regulation of amino acid import across plasma membrane	11/21092	4.40E-02	4.20E-01	0.403462799	Agt	1
GO:0014824	artery smooth muscle contraction	11/21092	4.40E-02	4.20E-01	0.403462799	Agt	1
GO:0019511	peptidyl-proline hydroxylation	11/21092	4.40E-02	4.20E-01	0.403462799	Crtap	1
GO:0032341	aldosterone metabolic process	11/21092	4.40E-02	4.20E-01	0.403462799	Bmp5	1
GO:0051324	prophase	11/21092	4.40E-02	4.20E-01	0.403462799	Mlh1	1
GO:0060405	regulation of penile erection	11/21092	4.40E-02	4.20E-01	0.403462799	P2ry2	1
GO:0060586	multicellular organismal iron ion homeostasis	11/21092	4.40E-02	4.20E-01	0.403462799	Hfe	1
GO:0086016	AV node cell action potential	11/21092	4.40E-02	4.20E-01	0.403462799	Scn10a	1
GO:0086027	AV node cell to bundle of His cell signaling	11/21092	4.40E-02	4.20E-01	0.403462799	Scn10a	1
GO:0097084	vascular smooth muscle cell development	11/21092	4.40E-02	4.20E-01	0.403462799	Comp	1
GO:1903867	extraembryonic membrane development	11/21092	4.40E-02	4.20E-01	0.403462799	Bmp5	1
GO:0055001	muscle cell development	195/21092	4.56E-02	4.20E-01	0.403462799	Comp/Lmod2/Agt	3
GO:0014910	regulation of smooth muscle cell migration	85/21092	4.72E-02	4.20E-01	0.403462799	Agt/P2ry2	2
GO:0000279	M phase	12/21092	4.79E-02	4.20E-01	0.403462799	Mlh1	1
GO:0000963	mitochondrial RNA processing	12/21092	4.79E-02	4.20E-01	0.403462799	Mto1	1
GO:0001542	ovulation from ovarian follicle	12/21092	4.79E-02	4.20E-01	0.403462799	Agt	1
GO:0002016	regulation of blood volume by renin-angiotensin	12/21092	4.79E-02	4.20E-01	0.403462799	Agt	1
GO:0006098	pentose-phosphate shunt	12/21092	4.79E-02	4.20E-01	0.403462799	Tkt	1

GO:0008212	mineralocorticoid metabolic process	12/21092	4.79E-02	4.20E-01	0.403462799	Bmp5	1
GO:0015809	arginine transport	12/21092	4.79E-02	4.20E-01	0.403462799	Agt	1
GO:0030432	peristalsis	12/21092	4.79E-02	4.20E-01	0.403462799	Agt	1
GO:0032308	positive regulation of prostaglandin secretion	12/21092	4.79E-02	4.20E-01	0.403462799	P2ry2	1
GO:0032353	negative regulation of hormone biosynthetic process	12/21092	4.79E-02	4.20E-01	0.403462799	Bmp5	1
GO:0033275	actin-myosin filament sliding	12/21092	4.79E-02	4.20E-01	0.403462799	Myh7	1
GO:0034309	primary alcohol biosynthetic process	12/21092	4.79E-02	4.20E-01	0.403462799	Bmp5	1
GO:0042271	susceptibility to natural killer cell mediated cytotoxicity	12/21092	4.79E-02	4.20E-01	0.403462799	Hfe	1
GO:0048143	astrocyte activation	12/21092	4.79E-02	4.20E-01	0.403462799	Agt	1
GO:0070471	uterine smooth muscle contraction	12/21092	4.79E-02	4.20E-01	0.403462799	Agt	1
GO:0071281	cellular response to iron ion	12/21092	4.79E-02	4.20E-01	0.403462799	Hfe	1
GO:0071468	cellular response to acidic pH	12/21092	4.79E-02	4.20E-01	0.403462799	Slc38a3	1
GO:0098762	meiotic cell cycle phase	12/21092	4.79E-02	4.20E-01	0.403462799	Mlh1	1
GO:0098764	meiosis I cell cycle phase	12/21092	4.79E-02	4.20E-01	0.403462799	Mlh1	1
GO:1900244	positive regulation of synaptic vesicle endocytosis	12/21092	4.79E-02	4.20E-01	0.403462799	Snap91	1
GO:1902430	negative regulation of amyloid-beta formation	12/21092	4.79E-02	4.20E-01	0.403462799	Spon1	1
GO:1903961	positive regulation of anion transmembrane transport	12/21092	4.79E-02	4.20E-01	0.403462799	Agt	1

Supp. Table S7. List of Biological Processes (BP) at FDR 5% - ventricle

GO ID	Description	BgRatio	pvalue	p.adjust	qvalue	geneID	Count
GO:0021826	substrate-independent telencephalic tangential migration	10/21092	2.55E-04	1.52E-01	0.120425014	Drd2/Nrg1	2
GO:0021843	substrate-independent telencephalic tangential interneuron migration	10/21092	2.55E-04	1.52E-01	0.120425014	Drd2/Nrg1	2
GO:1901386	negative regulation of voltage-gated calcium channel activity	15/21092	5.90E-04	1.52E-01	0.120425014	Gnb5/Drd2	2
GO:0014706	striated muscle tissue development	441/21092	6.56E-04	1.52E-01	0.120425014	Plagl1/Tbx18/Ephb1/Ripor2/Nrg1/Ryr1	6

GO:0003161	cardiac conduction system development	16/21092	6.73E-04	1.52E-01	0.120425014	Tbx18/Nrg1	2
GO:0007409	axonogenesis	451/21092	7.38E-04	1.52E-01	0.120425014	Snap91/Ntn1/Drd2/Ephb1/Adcy1/Nrg1	6
GO:0060537	muscle tissue development	462/21092	8.36E-04	1.52E-01	0.120425014	Plagl1/Tbx18/Ephb1/Ripor2/Nrg1/Ryr1	6
GO:0061564	axon development	486/21092	1.09E-03	1.55E-01	0.122484614	Snap91/Ntn1/Drd2/Ephb1/Adcy1/Nrg1	6
GO:0007519	skeletal muscle tissue development	197/21092	1.31E-03	1.55E-01	0.122484614	Plagl1/Ephb1/Ripor2/Ryr1	4
GO:0071467	cellular response to pH	23/21092	1.40E-03	1.55E-01	0.122484614	Slc38a3/Hyal1	2
GO:0060538	skeletal muscle organ development	206/21092	1.54E-03	1.55E-01	0.122484614	Plagl1/Ephb1/Ripor2/Ryr1	4
GO:0050772	positive regulation of axonogenesis	98/21092	1.72E-03	1.55E-01	0.122484614	Snap91/Ntn1/Nrg1	3
GO:0043171	peptide catabolic process	26/21092	1.80E-03	1.55E-01	0.122484614	Ctsh/Chac1	2
GO:1901020	negative regulation of calcium ion transmembrane transporter activity	26/21092	1.80E-03	1.55E-01	0.122484614	Gnb5/Drd2	2
GO:0072522	purine-containing compound biosynthetic process	227/21092	2.20E-03	1.55E-01	0.122484614	Guca1b/Adcy1/Nt5e/Shmt1	4
GO:0042692	muscle cell differentiation	383/21092	2.28E-03	1.55E-01	0.122484614	Tbx18/Ripor2/Nrg1/Ryr1/Tnnt2	5
GO:0006506	GPI anchor biosynthetic process	31/21092	2.55E-03	1.55E-01	0.122484614	Pgap2/Pigf	2
GO:0042472	inner ear morphogenesis	113/21092	2.59E-03	1.55E-01	0.122484614	Ntn1/Tbx18/Ripor2	3
GO:0001964	startle response	32/21092	2.71E-03	1.55E-01	0.122484614	Drd2/Nrg1	2
GO:0006505	GPI anchor metabolic process	32/21092	2.71E-03	1.55E-01	0.122484614	Pgap2/Pigf	2
GO:1903170	negative regulation of calcium ion transmembrane transport	35/21092	3.24E-03	1.55E-01	0.122484614	Gnb5/Drd2	2
GO:0007517	muscle organ development	420/21092	3.38E-03	1.55E-01	0.122484614	Plagl1/Ephb1/Ripor2/Nrg1/Ryr1	5
GO:0050807	regulation of synapse organization	259/21092	3.53E-03	1.55E-01	0.122484614	Snap91/Ntn1/Drd2/Ephb1	4
GO:1901385	regulation of voltage-gated calcium channel activity	37/21092	3.62E-03	1.55E-01	0.122484614	Gnb5/Drd2	2
GO:0050808	synapse organization	427/21092	3.63E-03	1.55E-01	0.122484614	Snap91/Ntn1/Drd2/Ephb1/Nrg1	5
GO:0007616	long-term memory	38/21092	3.81E-03	1.55E-01	0.122484614	Drd2/Adcy1	2

GO:0048741	skeletal muscle fiber development	38/21092	3.81E-03	1.55E-01	0.122484614	Ripor2/Ryr1	2
GO:0060236	regulation of mitotic spindle organization	38/21092	3.81E-03	1.55E-01	0.122484614	Parp3/Ripor2	2
GO:0050803	regulation of synapse structure or activity	266/21092	3.88E-03	1.55E-01	0.122484614	Snap91/Ntn1/Drd2/Ephb1	4
GO:0009268	response to pH	40/21092	4.22E-03	1.55E-01	0.122484614	Slc38a3/Hyal1	2
GO:0042471	ear morphogenesis	135/21092	4.27E-03	1.55E-01	0.122484614	Ntn1/Tbx18/Ripor2	3
GO:0031279	regulation of cyclase activity	41/21092	4.43E-03	1.55E-01	0.122484614	Guca1b/Drd2	2
GO:2001258	negative regulation of cation channel activity	41/21092	4.43E-03	1.55E-01	0.122484614	Gnb5/Drd2	2
GO:0071496	cellular response to external stimulus	277/21092	4.48E-03	1.55E-01	0.122484614	Nprl2/Slc38a3/Ripor2/Ryr1	4
GO:0006024	glycosaminoglycan biosynthetic process	42/21092	4.64E-03	1.55E-01	0.122484614	Pxylp1/Hyal1	2
GO:0014904	myotube cell development	42/21092	4.64E-03	1.55E-01	0.122484614	Ripor2/Ryr1	2
GO:0090224	regulation of spindle organization	42/21092	4.64E-03	1.55E-01	0.122484614	Parp3/Ripor2	2
GO:0006936	muscle contraction	281/21092	4.71E-03	1.55E-01	0.122484614	Drd2/Ryr1/Scn10a/Tnnt2	4
GO:0007212	dopamine receptor signaling pathway	43/21092	4.86E-03	1.55E-01	0.122484614	Gnb5/Drd2	2
GO:0051339	regulation of lyase activity	43/21092	4.86E-03	1.55E-01	0.122484614	Guca1b/Drd2	2
GO:0098693	regulation of synaptic vesicle cycle	147/21092	5.41E-03	1.66E-01	0.131229692	Snap91/Drd2/Adcy1	3
GO:0006937	regulation of muscle contraction	148/21092	5.51E-03	1.66E-01	0.131229692	Ryr1/Scn10a/Tnnt1	3
GO:0090407	organophosphate biosynthetic process	474/21092	5.64E-03	1.66E-01	0.131229692	Guca1b/Pgap2/Adcy1/Shmt1/Pigf	5
GO:0090596	sensory organ morphogenesis	297/21092	5.73E-03	1.66E-01	0.131229692	Ntn1/Tbx18/Ephb1/Ripor2	4
GO:0051146	striated muscle cell differentiation	302/21092	6.07E-03	1.72E-01	0.136016787	Ripor2/Nrg1/Ryr1/Tnnt2	4
GO:0045927	positive regulation of growth	308/21092	6.50E-03	1.80E-01	0.141909734	Ntn1/Drd2/Nrg1/Hyal1	4
GO:0051954	positive regulation of amine transport	51/21092	6.78E-03	1.80E-01	0.141909734	Slc38a3/Drd2	2
GO:0048562	embryonic organ morphogenesis	315/21092	7.03E-03	1.80E-01	0.141909734	Ntn1/Tbx18/Ripor2/Hyal1	4
GO:0006023	aminoglycan biosynthetic process	52/21092	7.04E-03	1.80E-01	0.141909734	Pxylp1/Hyal1	2
GO:0045773	positive regulation of axon extension	52/21092	7.04E-03	1.80E-01	0.141909734	Ntn1/Nrg1	2

GO:0007416	synapse assembly	169/21092	7.94E-03	1.94E-01	0.153083885	Ntn1/Ephb1/Nrg1	3
GO:0009247	glycolipid biosynthetic process	56/21092	8.12E-03	1.94E-01	0.153083885	Pgap2/Pigf	2
GO:0006661	phosphatidylinositol biosynthetic process	59/21092	8.98E-03	1.94E-01	0.153083885	Pgap2/Pigf	2
GO:0010770	positive regulation of cell morphogenesis involved in differentiation	177/21092	9.01E-03	1.94E-01	0.153083885	Snap91/Ntn1/Nrg1	3
GO:0001764	neuron migration	181/21092	9.57E-03	1.94E-01	0.153083885	Ntn1/Drd2/Nrg1	3
GO:0048489	synaptic vesicle transport	181/21092	9.57E-03	1.94E-01	0.153083885	Snap91/Drd2/Adcy1	3
GO:0055002	striated muscle cell development	181/21092	9.57E-03	1.94E-01	0.153083885	Ripor2/Ryr1/Tnnt1	3
GO:0097480	establishment of synaptic vesicle localization	181/21092	9.57E-03	1.94E-01	0.153083885	Snap91/Drd2/Adcy1	3
GO:0051926	negative regulation of calcium ion transport	61/21092	9.58E-03	1.94E-01	0.153083885	Gnb5/Drd2	2
GO:0009988	cell-cell recognition	62/21092	9.88E-03	1.94E-01	0.153083885	Ephb1/Hspa1l	2
GO:0031669	cellular response to nutrient levels	185/21092	1.02E-02	1.94E-01	0.153083885	Nprl2/Slc38a3/Ryr1	3
GO:0042440	pigment metabolic process	63/21092	1.02E-02	1.94E-01	0.153083885	Cyb5r4/Shmt1	2
GO:0031638	zymogen activation	64/21092	1.05E-02	1.94E-01	0.153083885	Ctsh/Cideb	2
GO:0007269	neurotransmitter secretion	190/21092	1.09E-02	1.94E-01	0.153083885	Snap91/Drd2/Adcy1	3
GO:0099643	signal release from synapse	190/21092	1.09E-02	1.94E-01	0.153083885	Snap91/Drd2/Adcy1	3
GO:0032413	negative regulation of ion transmembrane transporter activity	66/21092	1.11E-02	1.94E-01	0.153083885	Gnb5/Drd2	2
GO:0097479	synaptic vesicle localization	192/21092	1.12E-02	1.94E-01	0.153083885	Snap91/Drd2/Adcy1	3
GO:0030307	positive regulation of cell growth	195/21092	1.17E-02	1.94E-01	0.153083885	Ntn1/Nrg1/Hyal1	3
GO:0055001	muscle cell development	195/21092	1.17E-02	1.94E-01	0.153083885	Ripor2/Ryr1/Tnnt1	3
GO:0022029	telencephalon cell migration	70/21092	1.25E-02	1.94E-01	0.153083885	Drd2/Nrg1	2
GO:0001505	regulation of neurotransmitter levels	376/21092	1.29E-02	1.94E-01	0.153083885	Snap91/Drd2/Adcy1/Shmt1	4
GO:0050770	regulation of axonogenesis	202/21092	1.29E-02	1.94E-01	0.153083885	Snap91/Ntn1/Nrg1	3
GO:0010822	positive regulation of mitochondrion organization	72/21092	1.32E-02	1.94E-01	0.153083885	Cideb/Hspa1l	2
GO:0021885	forebrain cell migration	73/21092	1.35E-02	1.94E-01	0.153083885	Drd2/Nrg1	2

GO:0048839	inner ear development	206/21092	1.36E-02	1.94E-01	0.153083885	Ntn1/Tbx18/Ripor2	3
GO:0003012	muscle system process	383/21092	1.37E-02	1.94E-01	0.153083885	Drd2/Ryr1/Scn10a/Tnnt2	4
GO:0060047	heart contraction	207/21092	1.37E-02	1.94E-01	0.153083885	Drd2/Scn10a/Tnnt1	3
GO:0051155	positive regulation of striated muscle cell differentiation	74/21092	1.39E-02	1.94E-01	0.153083885	Ripor2/Nrg1	2
GO:0007163	establishment or maintenance of cell polarity	208/21092	1.39E-02	1.94E-01	0.153083885	Snap91/Ephb1/Ripor2	3
GO:0032410	negative regulation of transporter activity	75/21092	1.42E-02	1.94E-01	0.153083885	Gnb5/Drd2	2
GO:0048639	positive regulation of developmental growth	210/21092	1.43E-02	1.94E-01	0.153083885	Ntn1/Drd2/Nrg1	3
GO:0048747	muscle fiber development	76/21092	1.46E-02	1.94E-01	0.153083885	Ripor2/Ryr1	2
GO:0006814	sodium ion transport	212/21092	1.46E-02	1.94E-01	0.153083885	Slc38a3/Drd2/Scn10a	3
GO:0031668	cellular response to extracellular stimulus	212/21092	1.46E-02	1.94E-01	0.153083885	Nprl2/Slc38a3/Ryr1	3
GO:0003015	heart process	215/21092	1.52E-02	1.94E-01	0.153083885	Drd2/Scn10a/Tnnt1	3
GO:0006664	glycolipid metabolic process	79/21092	1.57E-02	1.94E-01	0.153083885	Pgap2/Pigf	2
GO:0043279	response to alkaloid	79/21092	1.57E-02	1.94E-01	0.153083885	Drd2/Ryr1	2
GO:1901019	regulation of calcium ion transmembrane transporter activity	79/21092	1.57E-02	1.94E-01	0.153083885	Gnb5/Drd2	2
GO:0030073	insulin secretion	218/21092	1.58E-02	1.94E-01	0.153083885	Hmgn3/Drd2/Cyb5r4	3
GO:1903509	liposaccharide metabolic process	80/21092	1.61E-02	1.94E-01	0.153083885	Pgap2/Pigf	2
GO:0030203	glycosaminoglycan metabolic process	81/21092	1.64E-02	1.94E-01	0.153083885	Pxylp1/Hyal1	2
GO:1904063	negative regulation of cation transmembrane transport	82/21092	1.68E-02	1.94E-01	0.153083885	Gnb5/Drd2	2
GO:0099504	synaptic vesicle cycle	228/21092	1.77E-02	1.94E-01	0.153083885	Snap91/Drd2/Adcy1	3
GO:0016485	protein processing	229/21092	1.79E-02	1.94E-01	0.153083885	Ctsh/Cideb/Chac1	3
GO:0090257	regulation of muscle system process	232/21092	1.86E-02	1.94E-01	0.153083885	Ryr1/Scn10a/Tnnt1	3
GO:0021954	central nervous system neuron development	87/21092	1.88E-02	1.94E-01	0.153083885	Drd2/Ephb1	2
GO:0043583	ear development	236/21092	1.94E-02	1.94E-01	0.153083885	Ntn1/Tbx18/Ripor2	3
GO:0006497	protein lipidation	89/21092	1.96E-02	1.94E-01	0.153083885	Pgap2/Pigf	2

GO:0035914	skeletal muscle cell differentiation	89/21092	1.96E-02	1.94E-01	0.153083885	Plagl1/Ephb1	2
GO:0051149	positive regulation of muscle cell differentiation	89/21092	1.96E-02	1.94E-01	0.153083885	Ripor2/Nrg1	2
GO:0019932	second-messenger-mediated signaling	430/21092	2.00E-02	1.94E-01	0.153083885	Drd2/Ackr4/Adcy1/Nrg1	4
GO:0002027	regulation of heart rate	91/21092	2.05E-02	1.94E-01	0.153083885	Drd2/Scn10a	2
GO:0090068	positive regulation of cell cycle process	246/21092	2.16E-02	1.94E-01	0.153083885	Drd2/Ripor2/Hyal1	3
GO:0042158	lipoprotein biosynthetic process	94/21092	2.17E-02	1.94E-01	0.153083885	Pgap2/Pigf	2
GO:0099003	vesicle-mediated transport in synapse	249/21092	2.23E-02	1.94E-01	0.153083885	Snap91/Drd2/Adcy1	3
GO:0034766	negative regulation of ion transmembrane transport	98/21092	2.35E-02	1.94E-01	0.153083885	Gnb5/Drd2	2
GO:0006995	cellular response to nitrogen starvation	10/21092	2.39E-02	1.94E-01	0.153083885	Nprl2	1
GO:0007270	neuron-neuron synaptic transmission	10/21092	2.39E-02	1.94E-01	0.153083885	Drd2	1
GO:0009129	pyrimidine nucleoside monophosphate metabolic process	10/21092	2.39E-02	1.94E-01	0.153083885	Shmt1	1
GO:0009221	pyrimidine deoxyribonucleotide biosynthetic process	10/21092	2.39E-02	1.94E-01	0.153083885	Shmt1	1
GO:0014883	transition between fast and slow fiber	10/21092	2.39E-02	1.94E-01	0.153083885	Tnnt2	1
GO:0021554	optic nerve development	10/21092	2.39E-02	1.94E-01	0.153083885	Ephb1	1
GO:0031282	regulation of guanylate cyclase activity	10/21092	2.39E-02	1.94E-01	0.153083885	Guca1b	1
GO:0043562	cellular response to nitrogen levels	10/21092	2.39E-02	1.94E-01	0.153083885	Nprl2	1
GO:0046085	adenosine metabolic process	10/21092	2.39E-02	1.94E-01	0.153083885	Nt5e	1
GO:0048149	behavioral response to ethanol	10/21092	2.39E-02	1.94E-01	0.153083885	Drd2	1
GO:0051823	regulation of synapse structural plasticity	10/21092	2.39E-02	1.94E-01	0.153083885	Drd2	1
GO:0070142	synaptic vesicle budding	10/21092	2.39E-02	1.94E-01	0.153083885	Snap91	1
GO:1903749	positive regulation of establishment of protein localization to mitochondrion	10/21092	2.39E-02	1.94E-01	0.153083885	Hspa1l	1
GO:1905097	regulation of guanyl-nucleotide exchange factor activity	10/21092	2.39E-02	1.94E-01	0.153083885	Ripor2	1
GO:2000402	negative regulation of lymphocyte migration	10/21092	2.39E-02	1.94E-01	0.153083885	Ripor2	1
GO:2000807	regulation of synaptic vesicle clustering	10/21092	2.39E-02	1.94E-01	0.153083885	Snap91	1
GO:0006022	aminoglycan metabolic process	99/21092	2.40E-02	1.94E-01	0.153083885	Pxylp1/Hyal1	2
GO:0031346	positive regulation of cell projection organization	462/21092	2.53E-02	1.94E-01	0.153083885	Snap91/Ntn1/Ripor2/Nrg1	4

GO:0030336	negative regulation of cell migration	263/21092	2.57E-02	1.94E-01	0.153083885	Drd2/Ripor2/Nrg1	3
GO:0051650	establishment of vesicle localization	265/21092	2.62E-02	1.94E-01	0.153083885	Snap91/Drd2/Adcy1	3
GO:0048259	regulation of receptor-mediated endocytosis	104/21092	2.62E-02	1.94E-01	0.153083885	Snap91/Drd2	2
GO:2000300	regulation of synaptic vesicle exocytosis	104/21092	2.62E-02	1.94E-01	0.153083885	Drd2/Adcy1	2
GO:0006012	galactose metabolic process	11/21092	2.63E-02	1.94E-01	0.153083885	Glb1	1
GO:0006171	cAMP biosynthetic process	11/21092	2.63E-02	1.94E-01	0.153083885	Adcy1	1
GO:0006266	DNA ligation	11/21092	2.63E-02	1.94E-01	0.153083885	Parp3	1
GO:0009125	nucleoside monophosphate catabolic process	11/21092	2.63E-02	1.94E-01	0.153083885	Nt5e	1
GO:0021892	cerebral cortex GABAergic interneuron differentiation	11/21092	2.63E-02	1.94E-01	0.153083885	Drd2	1
GO:0034776	response to histamine	11/21092	2.63E-02	1.94E-01	0.153083885	Drd2	1
GO:0035999	tetrahydrofolate interconversion	11/21092	2.63E-02	1.94E-01	0.153083885	Shmt1	1
GO:0051967	negative regulation of synaptic transmission, glutamatergic	11/21092	2.63E-02	1.94E-01	0.153083885	Drd2	1
GO:0060159	regulation of dopamine receptor signaling pathway	11/21092	2.63E-02	1.94E-01	0.153083885	Drd2	1
GO:0060600	dichotomous subdivision of an epithelial terminal unit	11/21092	2.63E-02	1.94E-01	0.153083885	Ctsh	1
GO:0086016	AV node cell action potential	11/21092	2.63E-02	1.94E-01	0.153083885	Scn10a	1
GO:0086027	AV node cell to bundle of His cell signaling	11/21092	2.63E-02	1.94E-01	0.153083885	Scn10a	1
GO:0072521	purine-containing compound metabolic process	469/21092	2.65E-02	1.94E-01	0.153083885	Guca1b/Adcy1/Nt5e/Shmt1	4
GO:0009165	nucleotide biosynthetic process	267/21092	2.67E-02	1.94E-01	0.153083885	Guca1b/Adcy1/Shmt1	3
GO:0006986	response to unfolded protein	106/21092	2.72E-02	1.94E-01	0.153083885	Hspa1l/Chac1	2
GO:0046474	glycerophospholipid biosynthetic process	106/21092	2.72E-02	1.94E-01	0.153083885	Pgap2/Pigf	2
GO:0042752	regulation of circadian rhythm	107/21092	2.77E-02	1.94E-01	0.153083885	Drd2/Adcy1	2
GO:0051209	release of sequestered calcium ion into cytosol	107/21092	2.77E-02	1.94E-01	0.153083885	Drd2/Ryr1	2
GO:0030072	peptide hormone secretion	271/21092	2.78E-02	1.94E-01	0.153083885	Hmgn3/Drd2/Cyb5r4	3
GO:0070588	calcium ion transmembrane transport	271/21092	2.78E-02	1.94E-01	0.153083885	Gnb5/Drd2/Ryr1	3
GO:0051656	establishment of organelle localization	476/21092	2.78E-02	1.94E-01	0.153083885	Snap91/Ntn1/Drd2/Adcy1	4

GO:1902803	regulation of synaptic vesicle transport	108/21092	2.81E-02	1.94E-01	0.153083885	Drd2/Adcy1	2
GO:1901293	nucleoside phosphate biosynthetic process	274/21092	2.86E-02	1.94E-01	0.153083885	Guca1b/Adcy1/Shmt1	3
GO:0007052	mitotic spindle organization	109/21092	2.86E-02	1.94E-01	0.153083885	Parp3/Ripor2	2
GO:0030516	regulation of axon extension	109/21092	2.86E-02	1.94E-01	0.153083885	Ntn1/Nrg1	2
GO:0051283	negative regulation of sequestering of calcium ion	109/21092	2.86E-02	1.94E-01	0.153083885	Drd2/Ryr1	2
GO:0000963	mitochondrial RNA processing	12/21092	2.86E-02	1.94E-01	0.153083885	Mto1	1
GO:0007195	adenylate cyclase-inhibiting dopamine receptor signaling pathway	12/21092	2.86E-02	1.94E-01	0.153083885	Drd2	1
GO:0009265	2'-deoxyribonucleotide biosynthetic process	12/21092	2.86E-02	1.94E-01	0.153083885	Shmt1	1
GO:0014842	regulation of skeletal muscle satellite cell proliferation	12/21092	2.86E-02	1.94E-01	0.153083885	Ephb1	1
GO:0030432	peristalsis	12/21092	2.86E-02	1.94E-01	0.153083885	Drd2	1
GO:0032488	Cdc42 protein signal transduction	12/21092	2.86E-02	1.94E-01	0.153083885	Ntn1	1
GO:0046385	deoxyribose phosphate biosynthetic process	12/21092	2.86E-02	1.94E-01	0.153083885	Shmt1	1
GO:0071318	cellular response to ATP	12/21092	2.86E-02	1.94E-01	0.153083885	Ryr1	1
GO:0071468	cellular response to acidic pH	12/21092	2.86E-02	1.94E-01	0.153083885	Slc38a3	1
GO:0090494	dopamine uptake	12/21092	2.86E-02	1.94E-01	0.153083885	Drd2	1
GO:1900244	positive regulation of synaptic vesicle endocytosis	12/21092	2.86E-02	1.94E-01	0.153083885	Snap91	1
GO:2000146	negative regulation of cell motility	277/21092	2.94E-02	1.94E-01	0.153083885	Drd2/Ripor2/Nrg1	3
GO:0051282	regulation of sequestering of calcium ion	111/21092	2.96E-02	1.94E-01	0.153083885	Drd2/Ryr1	2
GO:0051208	sequestering of calcium ion	112/21092	3.01E-02	1.94E-01	0.153083885	Drd2/Ryr1	2
GO:0048568	embryonic organ development	490/21092	3.05E-02	1.94E-01	0.153083885	Ntn1/Tbx18/Ripor2/Hyal1	4
GO:0046467	membrane lipid biosynthetic process	113/21092	3.06E-02	1.94E-01	0.153083885	Pgap2/Pigf	2
GO:0006596	polyamine biosynthetic process	13/21092	3.10E-02	1.94E-01	0.153083885	Sms	1
GO:0009071	serine family amino acid catabolic process	13/21092	3.10E-02	1.94E-01	0.153083885	Shmt1	1
GO:0009219	pyrimidine deoxyribonucleotide metabolic process	13/21092	3.10E-02	1.94E-01	0.153083885	Shmt1	1
GO:0014841	skeletal muscle satellite cell proliferation	13/21092	3.10E-02	1.94E-01	0.153083885	Ephb1	1
GO:0030213	hyaluronan biosynthetic process	13/21092	3.10E-02	1.94E-01	0.153083885	Hyal1	1

GO:0031000	response to caffeine	13/21092	3.10E-02	1.94E-01	0.153083885	Ryr1	1
GO:0035810	positive regulation of urine volume	13/21092	3.10E-02	1.94E-01	0.153083885	Drd2	1
GO:0036270	response to diuretic	13/21092	3.10E-02	1.94E-01	0.153083885	Ryr1	1
GO:0060124	positive regulation of growth hormone secretion	13/21092	3.10E-02	1.94E-01	0.153083885	Drd2	1
GO:0060272	embryonic skeletal joint morphogenesis	13/21092	3.10E-02	1.94E-01	0.153083885	Hyal1	1
GO:0086067	AV node cell to bundle of His cell communication	13/21092	3.10E-02	1.94E-01	0.153083885	Scn10a	1
GO:0090493	catecholamine uptake	13/21092	3.10E-02	1.94E-01	0.153083885	Drd2	1
GO:0097202	activation of cysteine-type endopeptidase activity	13/21092	3.10E-02	1.94E-01	0.153083885	Cideb	1
GO:2001223	negative regulation of neuron migration	13/21092	3.10E-02	1.94E-01	0.153083885	Nrg1	1
GO:0051648	vesicle localization	283/21092	3.10E-02	1.94E-01	0.153083885	Snap91/Drd2/Adcy1	3
GO:1903828	negative regulation of cellular protein localization	114/21092	3.11E-02	1.94E-01	0.153083885	Ripor2/Dclk3	2
GO:1901136	carbohydrate derivative catabolic process	115/21092	3.16E-02	1.94E-01	0.153083885	Nt5e/Hyal1	2
GO:0051963	regulation of synapse assembly	117/21092	3.26E-02	1.94E-01	0.153083885	Ntn1/Ephb1	2
GO:0009416	response to light stimulus	290/21092	3.30E-02	1.94E-01	0.153083885	Guca1b/Drd2/Hyal1	3
GO:0071214	cellular response to abiotic stimulus	290/21092	3.30E-02	1.94E-01	0.153083885	Slc38a3/Ripor2/Hyal1	3
GO:0104004	cellular response to environmental stimulus	290/21092	3.30E-02	1.94E-01	0.153083885	Slc38a3/Ripor2/Hyal1	3
GO:0051604	protein maturation	291/21092	3.33E-02	1.94E-01	0.153083885	Ctsh/Cideb/Chac1	3
GO:0001771	immunological synapse formation	14/21092	3.33E-02	1.94E-01	0.153083885	Ephb1	1
GO:0006544	glycine metabolic process	14/21092	3.33E-02	1.94E-01	0.153083885	Shmt1	1
GO:0007194	negative regulation of adenylate cyclase activity	14/21092	3.33E-02	1.94E-01	0.153083885	Drd2	1
GO:0015802	basic amino acid transport	14/21092	3.33E-02	1.94E-01	0.153083885	Slc38a3	1
GO:0021781	glial cell fate commitment	14/21092	3.33E-02	1.94E-01	0.153083885	Nrg1	1
GO:0021984	adenohypophysis development	14/21092	3.33E-02	1.94E-01	0.153083885	Drd2	1
GO:0031223	auditory behavior	14/21092	3.33E-02	1.94E-01	0.153083885	Drd2	1
GO:0031987	locomotion involved in locomotory behavior	14/21092	3.33E-02	1.94E-01	0.153083885	Drd2	1
GO:0035815	positive regulation of renal sodium excretion	14/21092	3.33E-02	1.94E-01	0.153083885	Drd2	1

GO:0046033	AMP metabolic process	14/21092	3.33E-02	1.94E-01	0.153083885	Nt5e	1
GO:0046058	cAMP metabolic process	14/21092	3.33E-02	1.94E-01	0.153083885	Adcy1	1
GO:0048755	branching morphogenesis of a nerve	14/21092	3.33E-02	1.94E-01	0.153083885	Drd2	1
GO:0086069	bundle of His cell to Purkinje myocyte communication	14/21092	3.33E-02	1.94E-01	0.153083885	Scn10a	1
GO:0097154	GABAergic neuron differentiation	14/21092	3.33E-02	1.94E-01	0.153083885	Drd2	1
GO:0051952	regulation of amine transport	119/21092	3.36E-02	1.94E-01	0.153083885	Slc38a3/Drd2	2
GO:0014902	myotube differentiation	120/21092	3.41E-02	1.94E-01	0.153083885	Ripor2/Ryr1	2
GO:0060996	dendritic spine development	120/21092	3.41E-02	1.94E-01	0.153083885	Ephb1/Nrg1	2
GO:0042157	lipoprotein metabolic process	121/21092	3.47E-02	1.94E-01	0.153083885	Pgap2/Pigf	2
GO:0051153	regulation of striated muscle cell differentiation	121/21092	3.47E-02	1.94E-01	0.153083885	Ripor2/Nrg1	2
GO:0060048	cardiac muscle contraction	121/21092	3.47E-02	1.94E-01	0.153083885	Scn10a/Tnnt2	2
GO:0060326	cell chemotaxis	298/21092	3.53E-02	1.94E-01	0.153083885	Ephb1/Ackr4/Ripor2	3
GO:0009263	deoxyribonucleotide biosynthetic process	15/21092	3.57E-02	1.94E-01	0.153083885	Shmt1	1
GO:0014733	regulation of skeletal muscle adaptation	15/21092	3.57E-02	1.94E-01	0.153083885	Tnnt2	1
GO:0014857	regulation of skeletal muscle cell proliferation	15/21092	3.57E-02	1.94E-01	0.153083885	Ephb1	1
GO:0031280	negative regulation of cyclase activity	15/21092	3.57E-02	1.94E-01	0.153083885	Drd2	1
GO:0043129	surfactant homeostasis	15/21092	3.57E-02	1.94E-01	0.153083885	Ctsh	1
GO:0051974	negative regulation of telomerase activity	15/21092	3.57E-02	1.94E-01	0.153083885	Parp3	1
GO:0060134	prepulse inhibition	15/21092	3.57E-02	1.94E-01	0.153083885	Drd2	1
GO:0060192	negative regulation of lipase activity	15/21092	3.57E-02	1.94E-01	0.153083885	Snap91	1
GO:0072189	ureter development	15/21092	3.57E-02	1.94E-01	0.153083885	Tbx18	1
GO:0072498	embryonic skeletal joint development	15/21092	3.57E-02	1.94E-01	0.153083885	Hyal1	1
GO:0098926	postsynaptic signal transduction	15/21092	3.57E-02	1.94E-01	0.153083885	Nrg1	1
GO:0099527	postsynapse to nucleus signaling pathway	15/21092	3.57E-02	1.94E-01	0.153083885	Nrg1	1
GO:1903423	positive regulation of synaptic vesicle recycling	15/21092	3.57E-02	1.94E-01	0.153083885	Snap91	1
GO:2000369	regulation of clathrin-dependent endocytosis	15/21092	3.57E-02	1.94E-01	0.153083885	Snap91	1
GO:0015837	amine transport	123/21092	3.57E-02	1.94E-01	0.153083885	Slc38a3/Drd2	2

GO:0061387	regulation of extent of cell growth	124/21092	3.62E-02	1.94E-01	0.153083885	Ntn1/Nrg1	2
GO:0009582	detection of abiotic stimulus	125/21092	3.68E-02	1.94E-01	0.153083885	Guca1b/Ephb1	2
GO:0034763	negative regulation of transmembrane transport	125/21092	3.68E-02	1.94E-01	0.153083885	Gnb5/Drd2	2
GO:0046677	response to antibiotic	303/21092	3.69E-02	1.94E-01	0.153083885	Drd2/Cyb5r4/Hyal1	3
GO:0009581	detection of external stimulus	126/21092	3.73E-02	1.94E-01	0.153083885	Guca1b/Ephb1	2
GO:0006836	neurotransmitter transport	306/21092	3.78E-02	1.94E-01	0.153083885	Snap91/Drd2/Adcy1	3
GO:0035966	response to topologically incorrect protein	127/21092	3.79E-02	1.94E-01	0.153083885	Hspa1l/Chac1	2
GO:0097553	calcium ion transmembrane import into cytosol	127/21092	3.79E-02	1.94E-01	0.153083885	Drd2/Ryr1	2
GO:0002031	G protein-coupled receptor internalization	16/21092	3.80E-02	1.94E-01	0.153083885	Drd2	1
GO:0002098	tRNA wobble uridine modification	16/21092	3.80E-02	1.94E-01	0.153083885	Mto1	1
GO:0014856	skeletal muscle cell proliferation	16/21092	3.80E-02	1.94E-01	0.153083885	Ephb1	1
GO:0043968	histone H2A acetylation	16/21092	3.80E-02	1.94E-01	0.153083885	Morf4l1	1
GO:0046112	nucleobase biosynthetic process	16/21092	3.80E-02	1.94E-01	0.153083885	Shmt1	1
GO:0072672	neutrophil extravasation	16/21092	3.80E-02	1.94E-01	0.153083885	Ripor2	1
GO:2001015	negative regulation of skeletal muscle cell differentiation	16/21092	3.80E-02	1.94E-01	0.153083885	Ephb1	1
GO:0044282	small molecule catabolic process	308/21092	3.84E-02	1.94E-01	0.153083885	Glb1/Hyal1/Shmt1	3
GO:0051271	negative regulation of cellular component movement	311/21092	3.93E-02	1.94E-01	0.153083885	Drd2/Ripor2/Nrg1	3
GO:0007189	adenylate cyclase-activating G protein-coupled receptor signaling pathway	130/21092	3.95E-02	1.94E-01	0.153083885	Drd2/Adcy1	2
GO:0008037	cell recognition	131/21092	4.00E-02	1.94E-01	0.153083885	Ephb1/Hspa1l	2
GO:1902850	microtubule cytoskeleton organization involved in mitosis	131/21092	4.00E-02	1.94E-01	0.153083885	Parp3/Ripor2	2
GO:0007638	mechanosensory behavior	17/21092	4.03E-02	1.94E-01	0.153083885	Drd2	1
GO:0009070	serine family amino acid biosynthetic process	17/21092	4.03E-02	1.94E-01	0.153083885	Shmt1	1
GO:0010224	response to UV-B	17/21092	4.03E-02	1.94E-01	0.153083885	Hyal1	1
GO:0032225	regulation of synaptic transmission, dopaminergic	17/21092	4.03E-02	1.94E-01	0.153083885	Drd2	1
GO:0042754	negative regulation of circadian rhythm	17/21092	4.03E-02	1.94E-01	0.153083885	Drd2	1
GO:0042756	drinking behavior	17/21092	4.03E-02	1.94E-01	0.153083885	Drd2	1

GO:0048875	chemical homeostasis within a tissue	17/21092	4.03E-02	1.94E-01	0.153083885	Ctsh	1
GO:0051350	negative regulation of lyase activity	17/21092	4.03E-02	1.94E-01	0.153083885	Drd2	1
GO:0097091	synaptic vesicle clustering	17/21092	4.03E-02	1.94E-01	0.153083885	Snap91	1
GO:0010821	regulation of mitochondrion organization	132/21092	4.06E-02	1.94E-01	0.153083885	Cideb/Hspa1l	2
GO:0017158	regulation of calcium ion-dependent exocytosis	132/21092	4.06E-02	1.94E-01	0.153083885	Drd2/Adcy1	2
GO:0046488	phosphatidylinositol metabolic process	133/21092	4.12E-02	1.94E-01	0.153083885	Pgap2/Pigf	2
GO:0050905	neuromuscular process	133/21092	4.12E-02	1.94E-01	0.153083885	Drd2/Nrg1	2
GO:0007204	positive regulation of cytosolic calcium ion concentration	317/21092	4.12E-02	1.94E-01	0.153083885	Drd2/Ackr4/Ryr1	3
GO:0030010	establishment of cell polarity	134/21092	4.17E-02	1.94E-01	0.153083885	Ephb1/Ripor2	2
GO:0048675	axon extension	134/21092	4.17E-02	1.94E-01	0.153083885	Ntn1/Nrg1	2
GO:0006595	polyamine metabolic process	18/21092	4.27E-02	1.94E-01	0.153083885	Sms	1
GO:0009309	amine biosynthetic process	18/21092	4.27E-02	1.94E-01	0.153083885	Sms	1
GO:0031643	positive regulation of myelination	18/21092	4.27E-02	1.94E-01	0.153083885	Nrg1	1
GO:0033604	negative regulation of catecholamine secretion	18/21092	4.27E-02	1.94E-01	0.153083885	Drd2	1
GO:0036120	cellular response to platelet-derived growth factor stimulus	18/21092	4.27E-02	1.94E-01	0.153083885	Hyal1	1
GO:0042219	cellular modified amino acid catabolic process	18/21092	4.27E-02	1.94E-01	0.153083885	Chac1	1
GO:0042401	cellular biogenic amine biosynthetic process	18/21092	4.27E-02	1.94E-01	0.153083885	Sms	1
GO:0043278	response to morphine	18/21092	4.27E-02	1.94E-01	0.153083885	Drd2	1
GO:0048268	clathrin coat assembly	18/21092	4.27E-02	1.94E-01	0.153083885	Snap91	1
GO:0050650	chondroitin sulfate proteoglycan biosynthetic process	18/21092	4.27E-02	1.94E-01	0.153083885	Pxylp1	1
GO:0050961	detection of temperature stimulus involved in sensory perception	18/21092	4.27E-02	1.94E-01	0.153083885	Ephb1	1
GO:0050965	detection of temperature stimulus involved in sensory perception of pain	18/21092	4.27E-02	1.94E-01	0.153083885	Ephb1	1
GO:0051481	negative regulation of cytosolic calcium ion concentration	18/21092	4.27E-02	1.94E-01	0.153083885	Drd2	1
GO:0060088	auditory receptor cell stereocilium organization	18/21092	4.27E-02	1.94E-01	0.153083885	Ripor2	1
GO:0070886	positive regulation of calcineurin-NFAT signaling cascade	18/21092	4.27E-02	1.94E-01	0.153083885	Nrg1	1
GO:0106058	positive regulation of calcineurin-mediated signaling	18/21092	4.27E-02	1.94E-01	0.153083885	Nrg1	1

GO:1904424	regulation of GTP binding	18/21092	4.27E-02	1.94E-01	0.153083885	Ripor2	1
GO:0046928	regulation of neurotransmitter secretion	137/21092	4.34E-02	1.95E-01	0.153818291	Drd2/Adcy1	2
GO:0009267	cellular response to starvation	139/21092	4.46E-02	1.95E-01	0.153818291	Nprl2/Slc38a3	2
GO:0040013	negative regulation of locomotion	328/21092	4.49E-02	1.95E-01	0.153818291	Drd2/Ripor2/Nrg1	3
GO:0001975	response to amphetamine	19/21092	4.50E-02	1.95E-01	0.153818291	Drd2	1
GO:0002097	tRNA wobble base modification	19/21092	4.50E-02	1.95E-01	0.153818291	Mto1	1
GO:0006027	glycosaminoglycan catabolic process	19/21092	4.50E-02	1.95E-01	0.153818291	Hyal1	1
GO:0010447	response to acidic pH	19/21092	4.50E-02	1.95E-01	0.153818291	Slc38a3	1
GO:0010996	response to auditory stimulus	19/21092	4.50E-02	1.95E-01	0.153818291	Drd2	1
GO:0014072	response to isoquinoline alkaloid	19/21092	4.50E-02	1.95E-01	0.153818291	Drd2	1
GO:0014808	release of sequestered calcium ion into cytosol by sarcoplasmic reticulum	19/21092	4.50E-02	1.95E-01	0.153818291	Ryr1	1
GO:0036119	response to platelet-derived growth factor	19/21092	4.50E-02	1.95E-01	0.153818291	Hyal1	1
GO:0043931	ossification involved in bone maturation	19/21092	4.50E-02	1.95E-01	0.153818291	Ryr1	1
GO:1903214	regulation of protein targeting to mitochondrion	19/21092	4.50E-02	1.95E-01	0.153818291	Hspa1l	1
GO:0007613	memory	141/21092	4.57E-02	1.95E-01	0.153818291	Drd2/Adcy1	2
GO:0045017	glycerolipid biosynthetic process	141/21092	4.57E-02	1.95E-01	0.153818291	Pgap2/Pigf	2
GO:0010634	positive regulation of epithelial cell migration	142/21092	4.63E-02	1.95E-01	0.153818291	Ctsh/Hyal1	2
GO:0019233	sensory perception of pain	142/21092	4.63E-02	1.95E-01	0.153818291	Ephb1/Scn10a	2
GO:0016079	synaptic vesicle exocytosis	143/21092	4.69E-02	1.95E-01	0.153818291	Drd2/Adcy1	2
GO:0002693	positive regulation of cellular extravasation	20/21092	4.73E-02	1.95E-01	0.153818291	Ripor2	1
GO:0010560	positive regulation of glycoprotein biosynthetic process	20/21092	4.73E-02	1.95E-01	0.153818291	Pxylp1	1
GO:0014075	response to amine	20/21092	4.73E-02	1.95E-01	0.153818291	Drd2	1
GO:0042451	purine nucleoside biosynthetic process	20/21092	4.73E-02	1.95E-01	0.153818291	Nt5e	1
GO:0046129	purine ribonucleoside biosynthetic process	20/21092	4.73E-02	1.95E-01	0.153818291	Nt5e	1
GO:0046653	tetrahydrofolate metabolic process	20/21092	4.73E-02	1.95E-01	0.153818291	Shmt1	1
GO:0060123	regulation of growth hormone secretion	20/21092	4.73E-02	1.95E-01	0.153818291	Drd2	1

GO:0070977	bone maturation	20/21092	4.73E-02	1.95E-01	0.153818291	Ryr1	1
GO:1903514	release of sequestered calcium ion into cytosol by endoplasmic reticulum	20/21092	4.73E-02	1.95E-01	0.153818291	Ryr1	1
GO:2000010	positive regulation of protein localization to cell surface	20/21092	4.73E-02	1.95E-01	0.153818291	Nrg1	1
GO:2000114	regulation of establishment of cell polarity	20/21092	4.73E-02	1.95E-01	0.153818291	Ripor2	1
GO:0010769	regulation of cell morphogenesis involved in differentiation	336/21092	4.76E-02	1.96E-01	0.154394136	Snap91/Ntn1/Nrg1	3
GO:0001976	neurological system process involved in regulation of systemic arterial blood pressure	21/21092	4.96E-02	1.99E-01	0.157260746	Drd2	1
GO:0007190	activation of adenylate cyclase activity	21/21092	4.96E-02	1.99E-01	0.157260746	Adcy1	1
GO:0007625	grooming behavior	21/21092	4.96E-02	1.99E-01	0.157260746	Drd2	1
GO:0015012	heparan sulfate proteoglycan biosynthetic process	21/21092	4.96E-02	1.99E-01	0.157260746	Pxylp1	1
GO:0031290	retinal ganglion cell axon guidance	21/21092	4.96E-02	1.99E-01	0.157260746	Ephb1	1
GO:0043501	skeletal muscle adaptation	21/21092	4.96E-02	1.99E-01	0.157260746	Tnnt2	1
GO:1901673	regulation of mitotic spindle assembly	21/21092	4.96E-02	1.99E-01	0.157260746	Ripor2	1
GO:0060402	calcium ion transport into cytosol	148/21092	4.99E-02	2.00E-01	0.157614938	Drd2/Ryr1	2

Supp Table S8. List of Differentially Expressed genes at FDR 5% in *Gnb5+/-* mice - atria

Ensembl ID	Gene Symbol	Gene name	baseMean	log2FoldChange	lfcSE	stat	pvalue	padj
ENSMUSG00000032224	Fam81a	family with sequence similarity 81, member A	292.8574685	2.335882638	0.253579957	9.211621721	3.21234E-20	6.22167E-16
ENSMUSG00000032192	Gnb5	guanine nucleotide binding protein (G protein), beta 5	588.3942165	-0.927754237	0.10834755	-8.562761538	1.10196E-17	1.06714E-13
ENSMUSG00000033419	Snap91	synaptosomal-associated protein 91	593.4008865	-0.644595104	0.121995504	-5.283761128	1.26558E-07	0.00081706
ENSMUSG00000051378	Kif18b	kinesin family member 18B	20.36546888	2.054560276	0.444055668	4.626807909	3.71345E-06	0.014384406
ENSMUSG00000078137	Ankrd63	ankyrin repeat domain 63	231.3397505	0.696118485	0.148990179	4.672244111	2.97927E-06	0.014384406
ENSMUSG00000053093	Myh7	myosin, heavy polypeptide 7, cardiac muscle, beta	251.3134444	-0.96720691	0.212994578	-4.540993103	5.59899E-06	0.018073528
ENSMUSG00000023979	Guca1b	guanylate cyclase activator 1B	58.67765502	0.974404672	0.217497591	4.480071111	7.46182E-06	0.020645783

Supp Table S9. List of Differentially Expressed genes at FDR 5% in *Gnb5+/-* mice - ventricle

Ensembl ID	Gene Symbol	Gene name	baseMean	log2FoldChange	lfcSE	stat	pvalue	padj
ENSMUSG00000032192	Gnb5	guanine nucleotide binding protein (G protein), beta 5	313.5699776	-0.960435275	0.123650627	-7.767330392	8.01576E-15	1.39723E-10
ENSMUSG00000032224	Fam81a	family with sequence similarity 81, member A	535.3572241	1.099542929	0.159014957	6.914713879	4.68809E-12	4.0859E-08

Supp Table S10. List of Differentially Expressed genes at FDR 5% in *Gnb5-/-* mice - cerebellum

Ensembl ID	Gene Symbol	Gene name	baseMean	log2FoldChange	lfcSE	stat	pvalue	padj
ENSMUSG00000032192	Gnb5	guanine nucleotide binding protein (G protein), beta 5	3776.684327	-2.827640286	0.055298441	-51.13417754	0.00E+00	< 2.00E-308
ENSMUSG00000023979	Guca1b	guanylate cyclase activator 1B	437.4550876	3.711107472	0.099175263	37.41968881	1.8697E-306	1.5621E-302
ENSMUSG00000032232	Cgnl1	cingulin-like 1	5161.732469	1.318444731	0.078033577	16.89586428	4.8259E-64	2.69E-60
ENSMUSG00000048758	Rpl29	ribosomal protein L29	167.003666	-2.662526944	0.179928843	-14.79766612	1.51654E-49	6.34E-46
ENSMUSG00000032556	Bfsp2	beaded filament structural protein 2, phakinin	65.92606322	-3.710156999	0.343429245	-10.8032646	3.32179E-27	1.11E-23
ENSMUSG00000043587	Pxylp1	2-phosphoxylose phosphatase 1	2090.786713	-0.93940513	0.089637192	-10.48008205	1.06651E-25	2.97E-22
ENSMUSG00000023277	Twf2	twinfilin actin binding protein 2	931.2249327	-0.527947875	0.052457731	-10.06425304	7.94884E-24	1.90E-20
ENSMUSG00000032498	Mlh1	mutL homolog 1	668.627558	-0.704540452	0.072809837	-9.676445968	3.79686E-22	7.93E-19
ENSMUSG00000020253	Ppm1m	protein phosphatase 1M	736.854351	-0.583377557	0.062857481	-9.2809567	1.67964E-20	3.12E-17
ENSMUSG00000062270	Morf4l1	mortality factor 4 like 1	4272.079558	-0.455630844	0.050708541	-8.985288001	2.58058E-19	4.31E-16
ENSMUSG00000062270	Morf4l1b	mortality factor 4 like 1B	4272.079558	-0.455630844	0.050708541	-8.985288001	2.58058E-19	4.31E-16
ENSMUSG00000032500	Dclk3	doublecortin-like kinase 3	227.5683369	1.633993963	0.194019129	8.421818885	3.70684E-17	5.63E-14
ENSMUSG00000032563	Mrpl3	mitochondrial ribosomal protein L3	2535.527483	-0.354146675	0.043154236	-8.206533358	2.27666E-16	3.17E-13
ENSMUSG00000104282	Gm37460	predicted gene 37460	24.0513232	-3.989910368	0.490489586	-8.134546545	4.13483E-16	5.31E-13
ENSMUSG00000090626	Tex9	testis expressed gene 9	591.5495903	-0.682683678	0.084985708	-8.032923324	9.51772E-16	1.14E-12
ENSMUSG00000032567	Aste1	asteroid homolog 1	261.2643017	-0.726216234	0.103776418	-6.997892664	2.59841E-12	2.89E-09
ENSMUSG00000036545	Adamts2	a disintegrin-like and metallopeptidase (reprolysin type) with thrombospondin type 1 motif, 2	865.4646909	-0.444637534	0.063619194	-6.989046931	2.7676E-12	2.89E-09
ENSMUSG00000043719	Col6a6	collagen, type VI, alpha 6	86.23142466	1.431557776	0.205901274	6.952641647	3.58509E-12	3.52E-09

ENSMUSG00000103164	Gm38150	predicted gene 38150	75.50295187	1.856335622	0.270406058	6.864992725	6.64946E-12	6.17E-09
ENSMUSG00000104286	Gm37457	predicted gene 37457	83.69435181	-1.396799984	0.20663308	-6.759808188	1.38175E-11	1.22E-08
ENSMUSG00000032494	Tdgf1	teratocarcinoma-derived growth factor 1	88.70981041	-1.228803344	0.187927365	-6.53871426	6.20499E-11	5.18E-08
ENSMUSG00000020096	Tbata	thymus, brain and testes associated	1971.627299	0.271356344	0.041688236	6.509182673	7.55608E-11	6.01E-08
ENSMUSG00000032418	Me1	malic enzyme 1, NADP(+)-dependent, cytosolic	2486.718734	0.398420712	0.061534107	6.474794711	9.49409E-11	7.21E-08
ENSMUSG00000002020	Ltbp2	latent transforming growth factor beta binding protein 2	4421.817622	-0.490281841	0.076725482	-6.390078353	1.65801E-10	1.20E-07
ENSMUSG00000032554	Trf	transferrin	516.363827	-0.885336834	0.14025914	-6.312150725	2.75184E-10	1.92E-07
ENSMUSG00000032495	Lrrc2	leucine rich repeat containing 2	337.4958733	-0.80567915	0.129576646	-6.217780565	5.04236E-10	3.37E-07
ENSMUSG00000102225	Gm37983	predicted gene 37983	24.91169952	1.872814001	0.301640962	6.208752252	5.34069E-10	3.43E-07
ENSMUSG00000023495	Pcbp4	poly(rC) binding protein 4	9608.983245	0.438156046	0.070693682	6.197951952	5.72026E-10	3.54E-07
ENSMUSG00000111348	Gm19531	predicted gene 19531	50.79214136	-1.15586438	0.18808935	-6.145294146	7.98155E-10	4.76E-07
ENSMUSG00000032383	Ppib	peptidylprolyl isomerase B	5797.045453	0.340285278	0.056151123	6.060168681	1.35979E-09	7.84E-07
ENSMUSG00000032221	Mns1	meiosis-specific nuclear structural protein 1	56.59125716	-1.175230186	0.195568968	-6.009287647	1.8634E-09	1.00E-06
ENSMUSG00000059003	Grin2a	glutamate receptor, ionotropic, NMDA2A (epsilon 1)	4488.448054	-0.246141364	0.040933749	-6.013164469	1.81936E-09	1.00E-06
ENSMUSG00000032411	Tfdp2	transcription factor Dp 2	2502.966265	0.41235966	0.0699472	5.895299004	3.74004E-09	1.95E-06
ENSMUSG00000023345	Poc1a	POC1 centriolar protein A	91.35994109	-0.946728825	0.160973191	-5.881282596	4.07099E-09	2.06E-06
ENSMUSG00000034898	Filip1	filamin A interacting protein 1	217.3345229	1.070995942	0.185443542	5.775320789	7.68066E-09	3.77E-06
ENSMUSG00000010057	Nprl2	NPR2 like, GATOR1 complex subunit	1400.200434	0.277235707	0.048216138	5.749853009	8.93211E-09	4.15E-06
ENSMUSG00000051590	Map3k19	mitogen-activated protein kinase kinase kinase 19	151.7662179	-0.72256102	0.125662208	-5.75002643	8.92295E-09	4.15E-06
ENSMUSG00000091735	Gpr62	G protein-coupled receptor 62	313.5180019	0.686846592	0.119742794	5.736016061	9.69295E-09	4.38E-06
ENSMUSG00000040536	Necab1	N-terminal EF-hand calcium binding protein 1	1182.606983	-0.675042081	0.119629524	-5.642771587	1.67334E-08	7.36E-06
ENSMUSG00000087466	A330041J22Rik	RIKEN cDNA A330041J22 gene	74.58228965	1.434589369	0.256512536	5.592667679	2.23607E-08	9.58E-06
ENSMUSG00000044037	Als2cl	ALS2 C-terminal like	1028.642711	-0.418008066	0.075093528	-5.566499248	2.59908E-08	1.09E-05
ENSMUSG00000070469	Adamtsl3	ADAMTS-like 3	113.9892968	-0.662897133	0.122069091	-5.43050764	5.6194E-08	2.29E-05
ENSMUSG00000023868	Pde10a	phosphodiesterase 10A	6880.473669	-0.676534037	0.125581847	-5.387196095	7.15653E-08	2.85E-05

ENSMUSG00000057802	Gm10030	predicted gene 10030	21.42515066	-1.977535225	0.375457621	-5.266999825	1.38671E-07	5.39E-05
ENSMUSG00000032360	Hcrtr2	hypocretin (orexin) receptor 2	28.96765092	-1.487211303	0.285983133	-5.200346213	1.98918E-07	7.55E-05
ENSMUSG00000024403	Atp6v1g2	ATPase, H+ transporting, lysosomal V1 subunit G2	10912.75865	0.157684878	0.030688324	5.138269387	2.7728E-07	1.03E-04
ENSMUSG00000102568	Gm37040	predicted gene 37040	36.43245889	-1.278054894	0.249989747	-5.112429236	3.18042E-07	1.16E-04
ENSMUSG00000033590	Myo5c	myosin VC	265.6388649	-0.549651459	0.109058116	-5.039986752	4.65564E-07	1.66E-04
ENSMUSG00000032872	Cyb5r4	cytochrome b5 reductase 4	2148.279031	-0.43931008	0.087835518	-5.001508373	5.68835E-07	1.98E-04
ENSMUSG00000047021	Cfap65	cilia and flagella associated protein 65	28.70220643	-1.970744625	0.396179632	-4.974371384	6.54597E-07	2.23E-04
ENSMUSG00000042761	Mrap2	melanocortin 2 receptor accessory protein 2	223.3199552	1.820064844	0.366864164	4.961140998	7.00803E-07	2.34E-04
ENSMUSG00000090150	Acad11	acyl-Coenzyme A dehydrogenase family, member 11	1333.016891	-0.242690676	0.049029394	-4.949901548	7.4251E-07	2.43E-04
ENSMUSG00000056596	Trnp1	TMF1-regulated nuclear protein 1	2707.369654	0.257405668	0.052080717	4.942437138	7.7152E-07	2.48E-04
ENSMUSG00000074415	3110039I08Rik	RIKEN cDNA 3110039I08 gene	858.8892546	-0.347692944	0.071049825	-4.893649586	9.8983E-07	3.12E-04
ENSMUSG00000045414	1190002N15Rik	RIKEN cDNA 1190002N15 gene	9479.974493	0.203857188	0.041965151	4.857773243	1.18713E-06	3.67E-04
ENSMUSG00000019929	Dcn	decorin	945.0632883	-0.39965724	0.083620332	-4.77942659	1.75796E-06	5.34E-04
ENSMUSG00000018659	Pnpo	pyridoxine 5'-phosphate oxidase	1853.661258	0.24366376	0.051217163	4.757463036	1.96041E-06	5.64E-04
ENSMUSG00000021265	Slc25a29	solute carrier family 25 (mitochondrial carrier, palmitoylcarnitine transporter), member 29	1086.377437	0.293381215	0.061607831	4.762076706	1.91611E-06	5.64E-04
ENSMUSG00000041506	Rrp9	RRP9, small subunit (SSU) processome component, homolog (yeast)	633.4693325	-0.348737041	0.073351895	-4.754301764	1.99133E-06	5.64E-04
ENSMUSG00000032436	Cmtm7	CKLF-like MARVEL transmembrane domain containing 7	225.5308249	-0.784340082	0.165355327	-4.743361447	2.10201E-06	5.85E-04
ENSMUSG00000070720	Tmem200b	transmembrane protein 200B	879.3241942	0.237178838	0.050148875	4.729494708	2.25079E-06	6.17E-04
ENSMUSG00000029822	Osbpl3	oxysterol binding protein-like 3	2125.961608	-0.339350961	0.073793709	-4.598643542	4.25251E-06	1.15E-03
ENSMUSG00000010048	Ifrd2	interferon-related developmental regulator 2	562.5877885	0.365850749	0.080007846	4.572685919	4.81512E-06	1.28E-03
ENSMUSG00000045967	Gpr158	G protein-coupled receptor 158	12290.27869	-0.241962539	0.053293038	-4.540227925	5.61934E-06	1.47E-03
ENSMUSG00000032356	Rasgrf1	RAS protein-specific guanine nucleotide-releasing factor 1	15290.93068	0.499536916	0.110479983	4.521515126	6.13986E-06	1.58E-03

ENSMUSG00000036760	Kcnk9	potassium channel, subfamily K, member 9	13243.63708	-0.292027828	0.06499848	-4.492840872	7.02793E-06	1.76E-03
ENSMUSG00000049624	Slc17a5	solute carrier family 17 (anion/sugar transporter), member 5	892.3986237	-0.346728247	0.077198643	-4.491377501	7.0764E-06	1.76E-03
ENSMUSG00000032497	Lrrfip2	leucine rich repeat (in FLII) interacting protein 2	1954.49032	-0.165756146	0.037035569	-4.47559331	7.61994E-06	1.87E-03
ENSMUSG00000024830	Rps6kb2	ribosomal protein S6 kinase, polypeptide 2	2329.153627	0.177815047	0.039942537	4.451771506	8.51648E-06	2.06E-03
ENSMUSG00000042210	Abhd14a	abhydrolase domain containing 14A	685.2533565	-0.289047191	0.065636456	-4.403759862	1.06391E-05	2.50E-03
ENSMUSG00000032570	Atp2c1	ATPase, Ca++-sequestering	7634.149512	0.190293715	0.043395191	4.38513372	1.15915E-05	2.69E-03
ENSMUSG00000085564	Gm12198	predicted gene 12198	144.1003058	-0.655784624	0.1506718	-4.35240452	1.34653E-05	3.08E-03
ENSMUSG00000025189	Cnnm1	cyclin M1	9936.963016	0.171522867	0.03971572	4.318765082	1.56905E-05	3.54E-03
ENSMUSG00000026156	B3gat2	beta-1,3-glucuronyltransferase 2 (glucuronosyltransferase S)	1542.762622	0.40134508	0.093300587	4.301635111	1.69542E-05	3.78E-03
ENSMUSG00000018697	Aatf	apoptosis antagonizing transcription factor	909.2901189	0.207262888	0.048456521	4.277296095	1.89177E-05	4.11E-03
ENSMUSG00000029695	Aass	aminoadipate-semialdehyde synthase	502.5914013	-0.540371692	0.126482917	-4.272289917	1.93476E-05	4.14E-03
ENSMUSG00000032936	Camkv	CaM kinase-like vesicle-associated	155.6519028	0.645556244	0.151277644	4.267360514	1.97799E-05	4.18E-03
ENSMUSG00000031274	Col4a5	collagen, type IV, alpha 5	423.3176062	-0.339340123	0.079636924	-4.261090277	2.03432E-05	4.25E-03
ENSMUSG00000025422	Agap2	ArfGAP with GTPase domain, ankyrin repeat and PH domain 2	9075.763147	0.168987623	0.039938014	4.231247511	2.32399E-05	4.79E-03
ENSMUSG00000040929	Rfx3	regulatory factor X, 3 (influences HLA class II expression)	1948.279304	-0.227743173	0.053898976	-4.225371062	2.38547E-05	4.86E-03
ENSMUSG00000046378	Asphd1	aspartate beta-hydroxylase domain containing 1	600.4949404	0.321122754	0.076280176	4.209779938	2.5562E-05	5.15E-03
ENSMUSG00000010660	Plcd1	phospholipase C, delta 1	287.7365686	-0.438423508	0.104711926	-4.186949119	2.82729E-05	5.56E-03
ENSMUSG00000026676	Ccdc3	coiled-coil domain containing 3	194.3461266	-0.787253495	0.187973755	-4.188103256	2.81296E-05	5.56E-03
ENSMUSG00000049303	Syt12	synaptotagmin XII	4738.805298	0.171600731	0.041042568	4.181042741	2.90175E-05	5.64E-03
ENSMUSG00000007594	Hapln4	hyaluronan and proteoglycan link protein 4	7762.924373	0.20525169	0.049244469	4.168015088	3.07264E-05	5.90E-03
ENSMUSG00000053702	Nebl	nebulette	4122.080911	-0.200274445	0.048467616	-4.132129069	3.59418E-05	6.82E-03
ENSMUSG00000070866	Zfp804a	zinc finger protein 804A	529.6035357	-0.273660923	0.066470521	-4.117026906	3.83791E-05	7.14E-03
ENSMUSG00000103891	Gm37941	predicted gene 37941	90.25718563	-0.590766007	0.143506106	-4.116661118	3.84401E-05	7.14E-03

ENSMUSG00000033577	Myo6	myosin VI	7952.800259	-0.177308645	0.043222703	-4.102210917	4.09221E-05	7.51E-03
ENSMUSG00000032562	Gnai2	guanine nucleotide binding protein (G protein), alpha inhibiting 2	5695.835318	0.209104738	0.051108773	4.09136684	4.28838E-05	7.79E-03
ENSMUSG00000022548	Apod	apolipoprotein D	7327.803279	-0.302224918	0.074021475	-4.082935635	4.44703E-05	7.96E-03
ENSMUSG00000025889	Snca	synuclein, alpha	753.7428095	0.310662096	0.076126465	4.080868538	4.48677E-05	7.96E-03
ENSMUSG00000040003	Magi2	membrane associated guanylate kinase, WW and PDZ domain containing 2	3517.01469	-0.198280333	0.048611072	-4.078912966	4.52468E-05	7.96E-03
ENSMUSG00000032224	Fam81a	family with sequence similarity 81, member A	752.0267376	0.40135418	0.098857026	4.059945935	4.90841E-05	8.46E-03
ENSMUSG00000035941	Ibtk	inhibitor of Bruton agammaglobulinemia tyrosine kinase	2259.614768	-0.234174871	0.057652254	-4.061851089	4.86851E-05	8.46E-03
ENSMUSG00000053399	Adamts18	a disintegrin-like and metallopeptidase (reprolysin type) with thrombospondin type 1 motif, 18	1245.172925	-0.535900624	0.132150485	-4.055230107	5.0085E-05	8.54E-03
ENSMUSG00000031209	Heph	hephaestin	212.499617	-0.524851774	0.129709845	-4.046352637	5.20218E-05	8.78E-03
ENSMUSG00000045594	Glb1	galactosidase, beta 1	603.8171745	-0.251329503	0.062199	-4.040732209	5.32846E-05	8.90E-03
ENSMUSG00000039728	Slc6a5	solute carrier family 6 (neurotransmitter transporter, glycine), member 5	2131.525392	0.184557898	0.0457464	4.034369904	5.4749E-05	9.06E-03
ENSMUSG00000022816	Fstl1	follistatin-like 1	656.1358971	-0.313458386	0.077999396	-4.018728391	5.85131E-05	9.49E-03
ENSMUSG00000060961	Slc4a4	solute carrier family 4 (anion exchanger), member 4	26482.84028	-0.210742417	0.052420959	-4.020193892	5.81503E-05	9.49E-03
ENSMUSG00000032786	Alas1	aminolevulinic acid synthase 1	2264.965012	0.16544191	0.041256635	4.010067955	6.07013E-05	9.60E-03
ENSMUSG00000034780	B3galt1	UDP-Gal:betaGlcNAc beta 1,3-galactosyltransferase, polypeptide 1	1512.878245	-0.228990845	0.057113105	-4.009427354	6.08662E-05	9.60E-03
ENSMUSG00000042757	Tmem108	transmembrane protein 108	1743.966905	0.191530914	0.047761983	4.010112257	6.06899E-05	9.60E-03
ENSMUSG00000038540	Tmc3	transmembrane channel-like gene family 3	128.5246132	-0.635080101	0.158674431	-4.002409822	6.27006E-05	9.79E-03
ENSMUSG00000019899	Lama2	laminin, alpha 2	1767.500596	-0.307800775	0.077002817	-3.997266444	6.40782E-05	9.91E-03
ENSMUSG00000020331	Hcn2	hyperpolarization-activated, cyclic nucleotide-gated K+ 2	3684.614211	0.211210959	0.053011952	3.984213928	6.77039E-05	1.04E-02
ENSMUSG00000079470	Utp14b	UTP14B small subunit processome component	593.3329387	-0.43940743	0.110425779	-3.979210594	6.91445E-05	1.05E-02

ENSMUSG00000043873	Chil5	chitinase-like 5	569.1443709	-0.529904212	0.134141397	-3.950340648	7.804E-05	1.17E-02
ENSMUSG00000034891	Sncb	synuclein, beta	6033.619166	0.257865278	0.065369039	3.944761662	7.98794E-05	1.18E-02
ENSMUSG00000111874	Gm36539	predicted gene 36539	28.68067065	-1.151072889	0.2918086	-3.944616051	7.99279E-05	1.18E-02
ENSMUSG00000040813	Tex264	testis expressed gene 264	1627.318836	0.24003156	0.061051179	3.931644982	8.43666E-05	1.24E-02
ENSMUSG00000024145	Pigf	phosphatidylinositol glycan anchor biosynthesis, class F	527.8534165	0.269835764	0.068864084	3.918381649	8.91455E-05	1.30E-02
ENSMUSG00000056019	Zfp709	zinc finger protein 709	716.7501148	-0.221064446	0.05646221	-3.915263783	9.03054E-05	1.30E-02
ENSMUSG00000047793	Sned1	sushi, nidogen and EGF-like domains 1	5436.766785	-0.214764333	0.054903128	-3.911695799	9.16503E-05	1.31E-02
ENSMUSG00000054196	Cthrc1	collagen triple helix repeat containing 1	456.6616622	0.273577589	0.070468467	3.882269615	0.000103486	1.44E-02
ENSMUSG00000062296	Trank1	tetratricopeptide repeat and ankyrin repeat containing 1	8866.298066	-0.401596014	0.103457199	-3.881759962	0.000103703	1.44E-02
ENSMUSG00000032359	Ctsh	cathepsin H	293.9199692	-0.380690598	0.098160275	-3.878255222	0.000105208	1.45E-02
ENSMUSG00000044244	Il20rb	interleukin 20 receptor beta	4371.39349	-0.294150387	0.075956801	-3.872601062	0.00010768	1.47E-02
ENSMUSG00000022540	Rogdi	rogdi homolog	3549.588552	0.19537945	0.05061794	3.859885424	0.00011344	1.54E-02
ENSMUSG00000036875	Dna2	DNA replication helicase/nuclease 2	172.3546123	-0.491356217	0.128239542	-3.831550007	0.000127338	1.72E-02
ENSMUSG00000022131	Gpr180	G protein-coupled receptor 180	1001.298783	0.180560403	0.047197908	3.825601801	0.000130453	1.74E-02
ENSMUSG00000029304	Spp1	secreted phosphoprotein 1	314.3225449	-1.037931743	0.271691281	-3.820261514	0.00013331	1.77E-02
ENSMUSG00000032571	Pik3r4	phosphoinositide-3-kinase regulatory subunit 4	2100.770068	-0.151120322	0.039789316	-3.798012582	0.000145861	1.92E-02
ENSMUSG00000038665	Dgki	diacylglycerol kinase, iota	1078.595202	-0.375885813	0.099194535	-3.78938028	0.000151024	1.97E-02
ENSMUSG00000054976	Nyap2	neuronal tyrosine-phophorylated phosphoinositide 3-kinase adaptor 2	1169.835804	-0.298167703	0.078758509	-3.785847468	0.000153186	1.98E-02
ENSMUSG00000031865	Dctn1	dynactin 1	14438.60666	0.157031021	0.041575282	3.77702842	0.000158711	2.04E-02
ENSMUSG00000032344	Cgas	cyclic GMP-AMP synthase	20.25321073	-1.137586452	0.301649272	-3.771222276	0.00016245	2.07E-02
ENSMUSG00000085084	4930570G19Rik	RIKEN cDNA 4930570G19 gene	1161.071062	-0.278073956	0.073863903	-3.76467995	0.000166763	2.11E-02
ENSMUSG00000040138	Ndp	Norrie disease (pseudoglioma) (human)	707.6201088	0.219662722	0.058391047	3.761924736	0.000168611	2.12E-02
ENSMUSG00000055254	Ntrk2	neurotrophic tyrosine kinase, receptor, type 2	19030.20769	-0.126891755	0.033783148	-3.75606662	0.000172605	2.15E-02
ENSMUSG00000027425	Kat14	lysine acetyltransferase 14	1374.54001	0.198671813	0.053026405	3.746658187	0.000179206	2.22E-02

ENSMUSG00000002274	Metrn	meteorin, glial cell differentiation regulator	5167.733381	0.251300779	0.067293283	3.734411033	0.000188155	2.31E-02
ENSMUSG00000054555	Adam12	a disintegrin and metallopeptidase domain 12 (meltrin alpha)	250.2725205	-0.367994148	0.098586085	-3.73271897	0.000189424	2.31E-02
ENSMUSG00000020185	E2f7	E2F transcription factor 7	64.89049163	-0.647104213	0.173634674	-3.726814448	0.000193915	2.31E-02
ENSMUSG00000031558	Slit2	slit guidance ligand 2	128.0552607	-0.619583532	0.166242811	-3.726979397	0.000193788	2.31E-02
ENSMUSG00000032803	Cdv3	carnitine deficiency-associated gene expressed in ventricle 3	2963.464124	-0.225267786	0.060576528	-3.718730583	0.000200226	2.37E-02
ENSMUSG00000039033	Tasp1	taspase, threonine aspartase 1	555.9554491	0.22670657	0.061038063	3.714183568	0.000203861	2.40E-02
ENSMUSG00000032323	Cyp11a1	cytochrome P450, family 11, subfamily a, polypeptide 1	160.2036109	-0.54410811	0.146689928	-3.709239733	0.000207883	2.43E-02
ENSMUSG00000027737	Slc7a11	solute carrier family 7 (cationic amino acid transporter, y+ system), member 11	1540.871957	-0.358277877	0.0967587	-3.702797536	0.000213235	2.44E-02
ENSMUSG00000033152	Podxl2	podocalyxin-like 2	6112.985547	0.265277682	0.071596768	3.705162828	0.000211255	2.44E-02
ENSMUSG00000105230	Gm42433	predicted gene 42433	287.5596305	0.32790406	0.088599373	3.700974955	0.000214773	2.44E-02
ENSMUSG00000025780	Itih5	inter-alpha (globulin) inhibitor H5	3052.757631	-0.257421968	0.069613061	-3.697897575	0.000217393	2.45E-02
ENSMUSG00000062257	Opcml	opioid binding protein/cell adhesion molecule-like	5120.415454	0.229556926	0.062123271	3.695184118	0.000219728	2.46E-02
ENSMUSG00000002228	Ppm1j	protein phosphatase 1J	73.97859987	0.543936081	0.147487001	3.688027274	0.000225999	2.48E-02
ENSMUSG00000020467	Efemp1	epidermal growth factor-containing fibulin-like extracellular matrix protein 1	661.4773856	-0.246464103	0.066769489	-3.691268372	0.000223139	2.48E-02
ENSMUSG00000071793	2610005L07Rik	cadherin 11 pseudogene	499.0576741	-0.260764185	0.070694432	-3.688609945	0.000225483	2.48E-02
ENSMUSG00000037624	Kcnk2	potassium channel, subfamily K, member 2	546.4969263	-0.275003038	0.074638882	-3.684447469	0.000229199	2.50E-02
ENSMUSG00000032184	Lysmd2	LysM, putative peptidoglycan-binding, domain containing 2	1722.663597	0.179859137	0.048875377	3.679953949	0.000233276	2.52E-02
ENSMUSG00000053716	Dusp7	dual specificity phosphatase 7	851.3299296	-0.261817054	0.071153446	-3.67961174	0.000233589	2.52E-02
ENSMUSG00000001773	Folh1	folate hydrolase 1	417.0674349	-0.340484695	0.092777155	-3.669919545	0.000242627	2.60E-02
ENSMUSG00000058966	Fam57b	family with sequence similarity 57, member B	4089.571587	0.189611427	0.051728708	3.665497087	0.000246859	2.63E-02
ENSMUSG00000063382	Bcl9l	B cell CLL/lymphoma 9-like	2478.992746	0.160810433	0.043905694	3.662632777	0.000249636	2.64E-02
ENSMUSG00000039163	Cmc1	COX assembly mitochondrial protein 1	342.5573765	0.344219264	0.094239415	3.652603992	0.000259594	2.73E-02

ENSMUSG00000006611	Hfe	hemochromatosis	233.2512245	0.407642583	0.11167162	3.65036868	0.000261864	2.73E-02
ENSMUSG00000025666	Tmem47	transmembrane protein 47	6719.132151	-0.216149918	0.059318852	-3.643865528	0.000268574	2.79E-02
ENSMUSG00000043631	Ecm2	extracellular matrix protein 2, female organ and adipocyte specific	522.2468524	-0.450393032	0.124061234	-3.630409094	0.000282972	2.92E-02
ENSMUSG00000000168	Dlat	dihydrolipoamide S-acetyltransferase (E2 component of pyruvate dehydrogenase complex)	4583.776794	0.176427012	0.048640056	3.627195923	0.000286516	2.92E-02
ENSMUSG00000052353	Cemip	cell migration inducing protein, hyaluronan binding	1840.025575	-0.4649413	0.128287759	-3.624206261	0.00028985	2.94E-02
ENSMUSG00000010045	Tmem115	transmembrane protein 115	1344.974255	0.214213274	0.059281784	3.613475478	0.00030212	3.02E-02
ENSMUSG00000058571	Gpc6	glypican 6	1138.981461	-0.221737078	0.061346601	-3.614496551	0.000300932	3.02E-02
ENSMUSG00000021998	Lcp1	lymphocyte cytosolic protein 1	575.426901	-0.33184577	0.091996191	-3.607168583	0.000309557	3.06E-02
ENSMUSG00000099512	Gm28703	predicted gene 28703	42.47158017	-0.723172188	0.200554314	-3.605867029	0.000311112	3.06E-02
ENSMUSG00000026344	Lypd1	Ly6/Plaur domain containing 1	707.8577798	0.357304132	0.099205924	3.601641094	0.000316215	3.09E-02
ENSMUSG00000026875	Traf1	TNF receptor-associated factor 1	452.7246797	0.445913269	0.123949496	3.597539993	0.000321241	3.12E-02
ENSMUSG00000037685	Atp8a1	ATPase, aminophospholipid transporter (APLT), class I, type 8A, member 1	11419.74021	-0.152030474	0.042369287	-3.588223594	0.000332939	3.22E-02
ENSMUSG00000022551	Cyc1	cytochrome c-1	5969.488864	0.176594951	0.049237771	3.586574833	0.00033505	3.22E-02
ENSMUSG00000000881	Dlg3	discs large MAGUK scaffold protein 3	5293.782134	0.118328055	0.033009058	3.584714728	0.000337447	3.22E-02
ENSMUSG00000004187	Kifc2	kinesin family member C2	8872.227272	0.164258912	0.045857095	3.581973822	0.000341008	3.23E-02
ENSMUSG00000039252	Lgi2	leucine-rich repeat LGI family, member 2	6372.971886	0.318458404	0.088965961	3.579553376	0.000344182	3.23E-02
ENSMUSG00000046574	Prr12	proline rich 12	3312.873574	0.150871364	0.042148131	3.579550486	0.000344186	3.23E-02
ENSMUSG00000010054	Tusc2	tumor suppressor 2, mitochondrial calcium regulator	2502.522813	0.154214131	0.043214835	3.568546102	0.000358968	3.33E-02
ENSMUSG00000024777	Ppp2r5b	protein phosphatase 2, regulatory subunit B', beta	6579.099733	0.135258768	0.037954628	3.563696347	0.000365669	3.38E-02
ENSMUSG00000063428	Ddo	D-aspartate oxidase	1482.79067	-0.154868869	0.043574294	-3.554133786	0.000379226	3.48E-02
ENSMUSG00000042155	Klhl23	kelch-like 23	3422.227809	-0.108699883	0.03072909	-3.537360911	0.000404147	3.69E-02
ENSMUSG00000001227	Sema6b	sema domain, transmembrane domain (TM), and cytoplasmic domain, (semaphorin) 6B	1758.165062	0.156707228	0.044407639	3.528834961	0.000417393	3.79E-02

ENSMUSG00000037579	Kcnh3	potassium voltage-gated channel, subfamily H (eag-related), member 3	141.4872672	0.699261434	0.198678837	3.519556709	0.000432269	3.90E-02
ENSMUSG00000039838	Slc45a1	solute carrier family 45, member 1	2158.860514	0.248713208	0.070876463	3.509108621	0.000449611	4.04E-02
ENSMUSG00000029059	Fam213b	family with sequence similarity 213, member B	1756.94047	0.240099039	0.06861156	3.499396293	0.000466313	4.17E-02
ENSMUSG00000041695	Kcnj2	potassium inwardly-rectifying channel, subfamily J, member 2	142.9180509	-0.457228698	0.130737517	-3.497302913	0.000469988	4.18E-02
ENSMUSG00000018076	Med13l	mediator complex subunit 13-like	5781.01772	-0.173663194	0.049763156	-3.489794661	0.000483392	4.26E-02
ENSMUSG00000044134	Pheta1	PH domain containing endocytic trafficking adaptor 1	704.7746099	0.237777278	0.068167136	3.48815119	0.000486373	4.26E-02
ENSMUSG00000050511	Oprd1	opioid receptor, delta 1	20.04430459	-1.209406991	0.346726523	-3.488071752	0.000486517	4.26E-02
ENSMUSG00000030092	Cntn6	contactin 6	5331.154587	-0.182682189	0.052415205	-3.485289974	0.000491604	4.28E-02
ENSMUSG00000032295	Man2c1	mannosidase, alpha, class 2C, member 1	4727.558809	0.127253112	0.036673966	3.46984868	0.000520752	4.51E-02
ENSMUSG00000023764	Sfi1	Sfi1 homolog, spindle assembly associated (yeast)	1212.243325	0.224302065	0.064771647	3.462966855	0.000534254	4.59E-02
ENSMUSG00000024076	Vit	vitrin	66.44094499	-0.547881353	0.158244454	-3.462246786	0.000535686	4.59E-02
ENSMUSG00000028150	Rorc	RAR-related orphan receptor gamma	2262.687714	0.230559859	0.066619632	3.460839586	0.000538494	4.59E-02
ENSMUSG00000040651	Fam208a	family with sequence similarity 208, member A	4400.287323	-0.205270022	0.059351971	-3.458520737	0.00054315	4.61E-02
ENSMUSG00000032328	Tmem30a	transmembrane protein 30A	15615.31096	-0.114504148	0.033143918	-3.454755915	0.000550791	4.65E-02
ENSMUSG00000109724	Gm18194	predicted gene 18194	133.610084	-0.426838601	0.123598131	-3.45343896	0.000553488	4.65E-02
ENSMUSG00000033862	Cdk10	cyclin-dependent kinase 10	1241.950755	0.202461754	0.05868709	3.449851619	0.000560895	4.65E-02
ENSMUSG00000061046	Haghl	hydroxyacylglutathione hydrolase-like	3351.199806	0.194626781	0.056425654	3.449260539	0.000562124	4.65E-02
ENSMUSG00000078480	Mrpl48-ps	Mitochondrial ribosomal protein L48	27.58601781	-3.250561366	0.94190633	-3.451045249	0.00055842	4.65E-02
ENSMUSG00000032324	Tspan3	tetraspanin 3	9627.269076	0.132814628	0.03855294	3.44499351	0.000571073	4.70E-02
ENSMUSG00000023017	Asic1	acid-sensing (proton-gated) ion channel 1	5549.805749	0.176764615	0.051358748	3.441762535	0.000577937	4.73E-02
ENSMUSG00000099616	4932413F04Rik	RIKEN cDNA 4932413F04 gene	21.90064226	2.125104477	0.619335029	3.43126802	0.000600767	4.89E-02
ENSMUSG00000110945	Gm9856	predicted gene 9856	328.9491498	-0.365949833	0.106689059	-3.4300596	0.000603449	4.89E-02

Supp Table S11. List of Differentially Expressed genes at FDR 5% in *Gnb5-/-* mice - hippocampus

Ensembl ID	Gene Symbol	Gene name	baseMean	log2FoldChange	lfcSE	stat	pvalue	padj
ENSMUSG00000032192	Gnb5	guanine nucleotide binding protein (G protein), beta 5	2382.397101	-2.949092443	0.050530966	-58.36208266	0.00E+00	< 2.00E-308
ENSMUSG00000023979	Guca1b	guanylate cyclase activator 1B	267.9709549	3.578006541	0.118457105	30.20508174	2.03E-200	1.96E-196
ENSMUSG00000048758	Rpl29	ribosomal protein L29	186.1160919	-2.54505664	0.14267899	-17.83764128	3.61E-71	2.32E-67
ENSMUSG00000020253	Ppm1m	protein phosphatase 1M	677.7813768	-0.764173504	0.069114538	-11.05662471	2.04E-28	9.82E-25
ENSMUSG00000032556	Bfsp2	beaded filament structural protein 2, phakinin	72.5712779	-3.439485494	0.36690378	-9.374352839	6.96E-21	2.68E-17
ENSMUSG00000023345	Poc1a	POC1 centriolar protein A	290.9177971	-0.980344264	0.104992395	-9.337288312	9.88E-21	3.18E-17
ENSMUSG00000032221	Mns1	meiosis-specific nuclear structural protein 1	180.727792	1.133030106	0.149838879	7.56165631	3.98E-14	1.10E-10
ENSMUSG00000049624	Slc17a5	solute carrier family 17 (anion/sugar transporter), member 5	1358.227186	0.395134078	0.0552683	7.149379957	8.72E-13	2.10E-09
ENSMUSG00000032418	Me1	malic enzyme 1, NADP(+)-dependent, cytosolic	3220.61655	0.35094107	0.049212845	7.131086888	9.96E-13	2.13E-09
ENSMUSG00000032344	Cgas	cyclic GMP-AMP synthase	64.05394375	-1.297224737	0.183150018	-7.082853442	1.41E-12	2.48E-09
ENSMUSG00000087466	A330041J22Rik	RIKEN cDNA A330041J22 gene	106.9804268	1.041048682	0.146908797	7.086360423	1.38E-12	2.48E-09
ENSMUSG00000032383	Ppib	peptidylprolyl isomerase B	7246.634387	0.323390812	0.046451328	6.961928182	3.36E-12	5.39E-09
ENSMUSG00000034910	Pygo1	pygopus 1	4535.707067	0.296843044	0.042829899	6.930743563	4.19E-12	6.21E-09
ENSMUSG00000057802	Gm10030	predicted gene 10030	24.02046494	-2.238670246	0.325437036	-6.878965819	6.03E-12	8.31E-09
ENSMUSG00000103164	Gm38150	predicted gene 38150	26.51357105	1.792662303	0.27409376	6.540325124	6.14E-11	7.89E-08
ENSMUSG00000047257	Prss45	protease, serine 45	29.29370674	2.22072601	0.345290573	6.431470148	1.26E-10	1.52E-07
ENSMUSG00000042210	Abhd14a	abhydrolase domain containing 14A	1011.239637	-0.395645636	0.062995633	-6.280524786	3.37E-10	3.83E-07
ENSMUSG00000102225	Gm37983	predicted gene 37983	19.23587601	2.053502553	0.328382635	6.253383503	4.02E-10	4.30E-07
ENSMUSG00000113637	Gm7049	predicted gene 7049	22.35956108	2.599937147	0.417875517	6.221798223	4.91E-10	4.99E-07
ENSMUSG00000032498	Mlh1	mutL homolog 1	473.749797	-0.608376157	0.098140351	-6.199041977	5.68E-10	5.48E-07
ENSMUSG00000032349	Elovl5	ELOVL family member 5, elongation of long chain fatty acids (yeast)	3448.449111	0.266712933	0.043182619	6.176395514	6.56E-10	6.02E-07
ENSMUSG00000032872	Cyb5r4	cytochrome b5 reductase 4	1427.463459	-0.552583779	0.097467466	-5.669417721	1.43E-08	1.26E-05

ENSMUSG00000062270	Morf4l1	mortality factor 4 like 1	4261.150376	-0.370709156	0.066751219	-5.553593847	2.80E-08	2.35E-05
ENSMUSG00000062270	Morf4l1b	mortality factor 4 like 1B	4261.150376	-0.370709156	0.066751219	-5.553593847	2.80E-08	2.35E-05
ENSMUSG00000032232	Cgnl1	cingulin-like 1	716.6625714	-0.674264189	0.126477175	-5.331113623	9.76E-08	7.55E-05
ENSMUSG00000032563	Mrpl3	mitochondrial ribosomal protein L3	2653.667229	-0.241906517	0.045381018	-5.330566088	9.79E-08	7.55E-05
ENSMUSG00000029471	Camkk2	calcium/calmodulin-dependent protein kinase kinase 2, beta	7986.908004	0.28842287	0.055844882	5.164714513	2.41E-07	1.79E-04
ENSMUSG00000111348	Gm19531	predicted gene 19531	60.80809486	-0.967537	0.190133025	-5.088737203	3.60E-07	2.57E-04
ENSMUSG00000032363	Adamts7	a disintegrin-like and metallopeptidase (reprolysin type) with thrombospondin type 1 motif, 7	60.02830454	1.253658417	0.250364087	5.007341231	5.52E-07	3.79E-04
ENSMUSG00000034898	Filip1	filamin A interacting protein 1	818.7049445	1.116876389	0.2236231	4.994458936	5.90E-07	3.79E-04
ENSMUSG00000046186	Cd109	CD109 antigen	440.3239799	0.744000787	0.14896346	4.994518707	5.90E-07	3.79E-04
ENSMUSG00000032184	Lysmd2	LysM, putative peptidoglycan-binding, domain containing 2	1535.062665	0.192736213	0.039007063	4.941059295	7.77E-07	4.83E-04
ENSMUSG00000032567	Aste1	asteroid homolog 1	258.6818556	-0.411147502	0.08364275	-4.915518725	8.85E-07	5.34E-04
ENSMUSG00000004366	Sst	somatostatin	4564.046349	-0.619153137	0.128391349	-4.822389825	1.42E-06	8.05E-04
ENSMUSG00000086296	D030055H07 Rik	RIKEN cDNA 030055H07 gene	30.05950376	1.958950704	0.408226916	4.798680896	1.60E-06	8.80E-04
ENSMUSG00000029819	Npy	neuropeptide Y	939.2650548	-0.512512486	0.107467639	-4.768993613	1.85E-06	9.92E-04
ENSMUSG00000042208	0610010F05R ik	RIKEN cDNA 0610010F05 gene	2105.523782	0.161358008	0.034019647	4.743082913	2.10E-06	1.10E-03
ENSMUSG00000042073	Abhd14b	abhydrolase domain containing 14b	680.5734956	-0.292955368	0.061980538	-4.726570277	2.28E-06	1.16E-03
ENSMUSG00000028681	Ptch2	patched 2	163.9075961	0.837619832	0.177967416	4.706590954	2.52E-06	1.21E-03
ENSMUSG00000064023	Klk8	kallikrein related-peptidase 8	472.905183	0.558842709	0.118652722	4.709902128	2.48E-06	1.21E-03
ENSMUSG00000039474	Wfs1	wolframin ER transmembrane glycoprotein	6619.822236	0.444546447	0.094628776	4.6977935	2.63E-06	1.24E-03
ENSMUSG00000021680	Crhbp	corticotropin releasing hormone binding protein	811.9965294	-0.700244123	0.150359765	-4.657124341	3.21E-06	1.47E-03
ENSMUSG00000049303	Syt12	synaptotagmin XII	1361.722132	0.338352088	0.072743355	4.651312622	3.30E-06	1.48E-03
ENSMUSG00000104586	4921539H07R ik	RIKEN cDNA 4921539H07	315.8272611	0.702883487	0.151686402	4.633793656	3.59E-06	1.57E-03
ENSMUSG00000041506	Rrp9	RRP9, small subunit (SSU) processome component, homolog (yeast)	673.6407763	-0.305170804	0.066043718	-4.620739307	3.82E-06	1.64E-03

ENSMUSG00000032420	Nt5e	5' nucleotidase, ecto	175.8655902	0.583004051	0.126570941	4.606144538	4.10E-06	1.72E-03
ENSMUSG00000023982	Guca1a	guanylate cyclase activator 1a (retina)	10.42051793	2.196823203	0.478445803	4.591582138	4.40E-06	1.81E-03
ENSMUSG00000060935	Tmem263	transmembrane protein 263	1125.137161	0.21083974	0.045978252	4.585640582	4.53E-06	1.82E-03
ENSMUSG00000010054	Tusc2	tumor suppressor 2, mitochondrial calcium regulator	2669.280548	0.150507457	0.033050594	4.553850268	5.27E-06	2.03E-03
ENSMUSG00000016942	Tmprss6	transmembrane serine protease 6	39.72576369	1.593037625	0.349843922	4.553566674	5.27E-06	2.03E-03
ENSMUSG00000030966	Trim21	tripartite motif-containing 21	113.3212122	-0.693400583	0.152836079	-4.536890679	5.71E-06	2.16E-03
ENSMUSG00000030688	Stard10	START domain containing 10	541.7897091	-0.295630626	0.065426937	-4.518484851	6.23E-06	2.31E-03
ENSMUSG00000029361	Nos1	nitric oxide synthase 1, neuronal	8256.593264	-0.261130979	0.059373749	-4.398088118	1.09E-05	3.97E-03
ENSMUSG00000063297	Luzp2	leucine zipper protein 2	6275.556935	0.268145569	0.061330761	4.372122009	1.23E-05	4.39E-03
ENSMUSG00000032570	Atp2c1	ATPase, Ca++-sequestering	10045.80105	0.188841067	0.043261344	4.365122496	1.27E-05	4.46E-03
ENSMUSG00000043719	Col6a6	collagen, type VI, alpha 6	118.7663673	1.69637505	0.390171056	4.347772659	1.38E-05	4.74E-03
ENSMUSG00000032332	Col12a1	collagen, type XII, alpha 1	629.8618523	-0.433906638	0.100352904	-4.32380748	1.53E-05	5.02E-03
ENSMUSG00000053040	Aph1c	aph1 homolog C, gamma secretase subunit	494.1347982	-0.311844717	0.072119583	-4.323995034	1.53E-05	5.02E-03
ENSMUSG00000058587	Tmod3	tropomodulin 3	1118.986474	-0.281319427	0.065066984	-4.323535703	1.54E-05	5.02E-03
ENSMUSG00000043587	Pxylp1	2-phosphoxylose phosphatase 1	545.5374077	-0.597492552	0.139927712	-4.270008748	1.95E-05	6.28E-03
ENSMUSG00000037089	Slc35b2	solute carrier family 35, member B2	868.1670288	0.238115051	0.05583839	4.264360995	2.00E-05	6.34E-03
ENSMUSG00000054204	Alkal2	ALK and LTK ligand 2	136.1426888	-0.81219183	0.191945922	-4.231357575	2.32E-05	7.23E-03
ENSMUSG00000032572	Col6a4	collagen, type VI, alpha 4	126.9727054	-2.511414656	0.595871353	-4.214692722	2.50E-05	7.66E-03
ENSMUSG00000085007	Gm11549	predicted gene 11549	231.5328462	0.4463464	0.106199532	4.202903639	2.64E-05	7.94E-03
ENSMUSG00000000805	Car4	carbonic anhydrase 4	2357.367529	0.308419263	0.073606666	4.19009963	2.79E-05	8.27E-03
ENSMUSG00000032330	Cox7a2	cytochrome c oxidase subunit 7A2	3013.00797	0.125559116	0.030057148	4.177346281	2.95E-05	8.49E-03
ENSMUSG00000110814	Gm38661	predicted gene 38661	14.96083878	-1.561989653	0.373750415	-4.179231894	2.92E-05	8.49E-03
ENSMUSG00000091345	Col6a5	collagen, type VI, alpha 5	174.2352851	1.016652505	0.244067905	4.165449379	3.11E-05	8.81E-03
ENSMUSG00000068428	Gmnc	geminin coiled-coil domain containing	264.2827313	-0.336898445	0.080973307	-4.160611156	3.17E-05	8.87E-03
ENSMUSG00000024883	Rin1	Ras and Rab interactor 1	4208.394122	0.206174191	0.049688679	4.149319198	3.33E-05	9.19E-03

ENSMUSG00000105230	Gm42433	predicted gene 42433	436.7842918	0.246770538	0.059606745	4.139976749	3.47E-05	9.44E-03
ENSMUSG00000071252	2210408I21Rik	RIKEN cDNA 2210408I21 gene	389.615445	0.264361422	0.064174253	4.119431228	3.80E-05	1.02E-02
ENSMUSG00000041923	Nol4	nucleolar protein 4	3286.050462	0.140254784	0.034100943	4.112929765	3.91E-05	1.03E-02
ENSMUSG00000019947	Arid5b	AT rich interactive domain 5B (MRF1-like)	1885.967968	-0.260157537	0.06335511	-4.106338656	4.02E-05	1.03E-02
ENSMUSG00000037104	Socs5	suppressor of cytokine signaling 5	5886.68313	0.107574506	0.026251141	4.097898351	4.17E-05	1.06E-02
ENSMUSG00000028971	Cort	cortistatin	67.60807817	-0.724694391	0.178109095	-4.068823039	4.73E-05	1.18E-02
ENSMUSG00000010057	Nprl2	NPR2 like, GATOR1 complex subunit	1429.63198	0.202997062	0.050049486	4.055927008	4.99E-05	1.23E-02
ENSMUSG00000032497	Lrrfip2	leucine rich repeat (in FLII) interacting protein 2	1294.793986	-0.201517371	0.049816198	-4.045217774	5.23E-05	1.26E-02
ENSMUSG00000040875	Osbpl10	oxysterol binding protein-like 10	644.5691274	-0.352970362	0.087207161	-4.047492849	5.18E-05	1.26E-02
ENSMUSG00000031209	Heph	hephaestin	349.4145748	-0.441492572	0.109271656	-4.040321062	5.34E-05	1.27E-02
ENSMUSG00000112441	Gm48898	predicted gene 48898	20.14550056	1.319734025	0.326804115	4.03830296	5.38E-05	1.27E-02
ENSMUSG00000014601	Strip1	striatin interacting protein 1	4618.315049	0.121491804	0.030263161	4.014511357	5.96E-05	1.37E-02
ENSMUSG00000040936	Ulk4	unc-51-like kinase 4	201.3074905	-0.524730265	0.132061386	-3.973381479	7.09E-05	1.61E-02
ENSMUSG00000038859	Baiap2l1	BAI1-associated protein 2-like 1	49.0839138	-1.306916288	0.331538163	-3.941978429	8.08E-05	1.81E-02
ENSMUSG00000016763	Scube1	signal peptide, CUB domain, EGF-like 1	1726.279322	0.426222434	0.108205935	3.93899311	8.18E-05	1.81E-02
ENSMUSG00000103133	Gm37303	predicted gene 37303	77.94310654	0.522106934	0.133550723	3.909427989	9.25E-05	2.03E-02
ENSMUSG00000004951	Hspb1	heat shock protein 1	151.4367051	-0.835319314	0.213951004	-3.904255167	9.45E-05	2.05E-02
ENSMUSG00000090877	Hspa1b	heat shock protein 1B	392.1558983	-0.763255749	0.19636514	-3.88692082	1.02E-04	2.18E-02
ENSMUSG00000029462	Vps29	VPS29 retromer complex component	3845.833264	0.14493881	0.037518194	3.863160675	1.12E-04	2.32E-02
ENSMUSG00000042444	Mindy2	MINDY lysine 48 deubiquitinase 2	3612.316289	-0.152283466	0.039398177	-3.865241432	1.11E-04	2.32E-02
ENSMUSG00000108325	NA	NA	93.73890629	0.5200387	0.134475044	3.867176279	1.10E-04	2.32E-02
ENSMUSG00000035941	Ibtk	inhibitor of Bruton agammaglobulinemia tyrosine kinase	1211.17404	-0.205862348	0.053432389	-3.852763275	1.17E-04	2.40E-02
ENSMUSG00000045327	6330549D23Rik	RIKEN cDNA 6330549D23 gene	49.6841459	-0.674934781	0.1754258	-3.847408893	1.19E-04	2.42E-02
ENSMUSG00000032012	Nectin1	nectin cell adhesion molecule 1	2017.659804	0.237274266	0.061873515	3.834827627	1.26E-04	2.52E-02

ENSMUSG00000051910	Sox6	SRY (sex determining region Y)-box 6	1109.249799	-0.210449817	0.054915382	-3.832256303	1.27E-04	2.52E-02
ENSMUSG00000033644	Piwil2	piwi-like RNA-mediated gene silencing 2	81.66252996	-0.533317618	0.139890545	-3.812392175	1.38E-04	2.71E-02
ENSMUSG00000006522	Itih3	inter-alpha trypsin inhibitor, heavy chain 3	1084.136711	-0.295092903	0.078005203	-3.782990024	1.55E-04	2.99E-02
ENSMUSG00000107092	Gm7993	predicted gene 7993	27.55659689	3.745229986	0.998444348	3.751065337	1.76E-04	3.36E-02
ENSMUSG00000038370	Pcp4l1	Purkinje cell protein 4-like 1	1329.318487	-0.232791453	0.063060501	-3.69155731	2.23E-04	4.17E-02
ENSMUSG00000048001	Hes5	hes family bHLH transcription factor 5	210.1360801	0.574406535	0.155524959	3.693339899	2.21E-04	4.17E-02
ENSMUSG00000051590	Map3k19	mitogen-activated protein kinase kinase kinase 19	332.5326268	-0.512141061	0.138814957	-3.689379518	2.25E-04	4.17E-02
ENSMUSG00000032265	Tent5a	terminal nucleotidyltransferase 5A	327.0098402	-0.714753787	0.195490594	-3.656205511	2.56E-04	4.70E-02

Supp Table S12. List of Differentially Expressed genes at FDR 5% in *Gnb5-/-* mice - cerebral cortex

Ensembl ID	Gene Symbol	Gene name	baseMean	log2FoldChange	lfcSE	stat	pvalue	padj
ENSMUSG00000032192	Gnb5	guanine nucleotide binding protein (G protein), beta 5	3593.342841	-3.062918872	0.090085387	-34.0001745	2.21E-253	4.84E-249
ENSMUSG00000023979	Guca1b	guanylate cyclase activator 1B	263.8918466	3.835511755	0.13316811	28.80202898	2.0231E-182	2.209E-178
ENSMUSG00000048758	Rpl29	ribosomal protein L29	264.164767	-2.731697404	0.15217746	-17.95073601	4.73586E-72	3.44739E-68
ENSMUSG00000020253	Ppm1m	protein phosphatase 1M	894.8693801	-1.212158437	0.086719969	-13.97784672	2.1283E-44	1.16195E-40
ENSMUSG00000032221	Mns1	meiosis-specific nuclear structural protein 1	116.3525616	1.634839206	0.147435847	11.08847841	1.42694E-28	6.23231E-25
ENSMUSG00000023345	Poc1a	POC1 centriolar protein A	386.6670779	-1.089635159	0.121899515	-8.938798156	3.93426E-19	1.43194E-15
ENSMUSG00000032332	Col12a1	collagen, type XII, alpha 1	887.9004528	-0.885695463	0.103581894	-8.550678424	1.22367E-17	3.8175E-14
ENSMUSG00000111765	Gm10635	predicted gene 10635	51.20226751	-3.314939998	0.393896818	-8.415757245	3.90365E-17	1.0656E-13
ENSMUSG00000111348	Gm19531	predicted gene 19531	101.5467041	-1.241020024	0.148741013	-8.343495822	7.2126E-17	1.7501E-13
ENSMUSG00000047257	Prss45	protease, serine 45	34.35647461	2.296734155	0.277403272	8.279405412	1.23792E-16	2.70337E-13
ENSMUSG00000042210	Abhd14a	abhydrolase domain containing 14A	1032.445648	-0.613596969	0.083391587	-7.358020052	1.86658E-13	3.70567E-10
ENSMUSG00000102225	Gm37983	predicted gene 37983	19.57885383	2.58186601	0.362084881	7.130554587	9.99653E-13	1.8192E-09
ENSMUSG00000043719	Col6a6	collagen, type VI, alpha 6	105.0713081	1.589103099	0.247685384	6.41581298	1.40073E-10	2.35302E-07

ENSMUSG00000032349	Elovl5	ELOVL family member 5, elongation of long chain fatty acids (yeast)	3648.481725	0.321846446	0.051309014	6.272707688	3.54823E-10	5.53473E-07
ENSMUSG00000032418	Me1	malic enzyme 1, NADP(+)-dependent, cytosolic	2016.009287	0.340643445	0.054688731	6.228768528	4.70116E-10	6.84426E-07
ENSMUSG00000010044	Zmynd10	zinc finger, MYND domain containing 10	228.8723475	0.730365544	0.120958409	6.038154333	1.55887E-09	1.79172E-06
ENSMUSG00000032498	Mlh1	mutL homolog 1	521.8321994	-0.490752727	0.081153472	-6.047217911	1.47368E-09	1.79172E-06
ENSMUSG00000032872	Cyb5r4	cytochrome b5 reductase 4	1413.346395	-0.564165726	0.093231634	-6.051226418	1.43747E-09	1.79172E-06
ENSMUSG00000087466	A330041J22 Rik	RIKEN cDNA A330041J22 gene	81.90474134	1.204927988	0.202332849	5.955177308	2.5979E-09	2.83665E-06
ENSMUSG00000049555	Tmie	transmembrane inner ear	241.8759334	0.63716907	0.114389213	5.570184912	2.54469E-08	2.64624E-05
ENSMUSG00000032556	Bfsp2	beaded filament structural protein 2, phakinin	85.4861346	-2.94717596	0.53990551	-5.458688424	4.79665E-08	4.63913E-05
ENSMUSG00000057802	Gm10030	predicted gene 10030	22.31636754	-1.9157509	0.351165297	-5.45541065	4.88598E-08	4.63913E-05
ENSMUSG00000062270	Morf4l1	mortality factor 4 like 1	3862.642941	-0.355532571	0.066892551	-5.314979978	1.06669E-07	9.706E-05
ENSMUSG00000062270	Morf4l1b	mortality factor 4 like 1B	3862.642941	-0.355532571	0.066892551	-5.314979978	1.06669E-07	9.706E-05
ENSMUSG00000103164	Gm38150	predicted gene 38150	23.78691025	1.84771035	0.366887132	5.036181944	4.74909E-07	0.000414843
ENSMUSG00000032383	Ppib	peptidylprolyl isomerase B	6545.594919	0.317038174	0.064051057	4.949772724	7.43002E-07	0.000624064
ENSMUSG00000059146	Ntrk3	neurotrophic tyrosine kinase, receptor, type 3	10681.1642	-0.195870941	0.040335881	-4.855997647	1.19782E-06	0.000968816
ENSMUSG00000059146	E430016F16 Rik	RIKEN cDNA E430016F16 gene	10681.1642	-0.195870941	0.040335881	-4.855997647	1.19782E-06	0.000968816
ENSMUSG00000032497	Lrrfip2	leucine rich repeat (in FLII) interacting protein 2	1541.136996	-0.220140185	0.04568305	-4.81885913	1.44381E-06	0.001126072
ENSMUSG00000109975	Gm19196	predicted gene 19196	76.97292567	-0.83775478	0.179372068	-4.670486274	3.00488E-06	0.002262775
ENSMUSG00000097099	Gm9917	predicted gene 9917	87.59711317	-0.72766991	0.158976579	-4.577214553	4.71208E-06	0.003334375
ENSMUSG00000110996	Gm36251	predicted gene, 36251	83.08922865	-2.301491121	0.502918057	-4.576274579	4.73329E-06	0.003334375
ENSMUSG00000024143	Rhoq	ras homolog family member Q	1994.563327	0.27220004	0.059757079	4.555109551	5.23583E-06	0.003573126
ENSMUSG00000055125	M5C1000I18 Rik	RIKEN cDNA M5C1000I18	169.1051477	-0.512026751	0.116302137	-4.4025567	1.06983E-05	0.007079656
ENSMUSG00000043587	Pxylp1	2-phosphoxylose phosphatase 1	541.0845473	-0.522547059	0.12057503	-4.333791642	1.46563E-05	0.009413649
ENSMUSG00000032370	Lactb	lactamase, beta	508.7191939	-0.370527801	0.086670063	-4.275153232	1.91006E-05	0.011586629
ENSMUSG00000063953	Amd2	S-adenosylmethionine decarboxylase 2	13.33707831	2.326043682	0.551895854	4.214642425	2.50174E-05	0.014765689

ENSMUSG00000041506	Rrp9	RRP9, small subunit (SSU) processome component, homolog (yeast)	741.5546544	-0.371086466	0.089204231	-4.159964852	3.18297E-05	0.018292002
ENSMUSG00000034910	Pygo1	pygopus 1	2350.941427	0.238237403	0.057597268	4.13626219	3.53009E-05	0.019766684
ENSMUSG00000033208	S100b	S100 protein, beta polypeptide, neural	4168.970089	0.202698353	0.049322006	4.109693985	3.96184E-05	0.021252459
ENSMUSG00000042073	Abhd14b	abhydrolase domain containing 14b	685.2510238	-0.305729814	0.074452684	-4.106363902	4.01936E-05	0.021252459
ENSMUSG00000091735	Gpr62	G protein-coupled receptor 62	243.7985731	0.456204569	0.111202042	4.102483748	4.08739E-05	0.021252459
ENSMUSG00000033730	Egr3	early growth response 3	6887.681122	-0.476370365	0.117456133	-4.055730036	4.9978E-05	0.025381829
ENSMUSG00000041460	Cacna2d4	calcium channel, voltage-dependent, alpha 2/delta subunit 4	115.497014	0.560303026	0.139981496	4.002693509	6.26254E-05	0.031082129
ENSMUSG00000039943	Plcb4	phospholipase C, beta 4	2966.652641	-0.243003116	0.061111924	-3.976361751	6.99776E-05	0.033959376
ENSMUSG00000032184	Lysmd2	LysM, putative peptidoglycan-binding, domain containing 2	1379.081352	0.192652009	0.049069682	3.926090417	8.63377E-05	0.04098789
ENSMUSG00000107092	Gm7993	predicted gene 7993	24.59807194	3.811634628	0.974363079	3.911924324	9.15636E-05	0.042543958
ENSMUSG00000032563	Mrpl3	mitochondrial ribosomal protein L3	2663.113172	-0.178171707	0.045609773	-3.906437045	9.3667E-05	0.042614597

Supp. Table S13. List of Biological Processes (BP) at FDR 5% - cerebellum

GO ID	Description	BgRatio	pvalue	p.adjust	qvalue	geneID	Count
GO:0042391	regulation of membrane potential	430/21092	7.30339E-05	0.164189734	0.156177167	Grin2a/Dcn/Snca/Tmem108/Hcn2/Dgki/Ntrk2/Kcnk2/Tusc2/Kcnh3/Kcnj2/Oprd1/Asic1	13
GO:0043949	regulation of cAMP-mediated signaling	64/21092	0.000200911	0.164189734	0.156177167	Gpr62/Pde10a/Mrap2/Gnai2/Cgas	5
GO:0006865	amino acid transport	145/21092	0.000227254	0.164189734	0.156177167	Slc25a29/Slc17a5/Myo6/Snca/Slc6a5/Ntrk2/Slc7a11	7
GO:0050808	synapse organization	427/21092	0.000271612	0.164189734	0.156177167	Filip1/Camkv/Col4a5/Zfp804a/Myo6/Snca/Magi2/Tmem108/Sncb/Dctn1/Ntrk2/Slc7a11	12
GO:0010039	response to iron ion	20/21092	0.000596278	0.288360066	0.274287904	Trf/Snca/Hfe	3
GO:0046928	regulation of neurotransmitter secretion	137/21092	0.001064507	0.3155174	0.300119942	Syt12/Myo6/Snca/Dgki/Ntrk2/Asic1	6
GO:0006022	aminoglycan metabolic process	99/21092	0.00148897	0.3155174	0.300119942	Pxylp1/Dcn/Chil5/Itih5/Cemip	5
GO:0043951	negative regulation of cAMP-mediated signaling	28/21092	0.001631142	0.3155174	0.300119942	Pde10a/Mrap2/Gnai2	3

GO:0050654	chondroitin sulfate proteoglycan metabolic process	29/21092	0.0018082	0.3155174	0.300119942	Pxylp1/Dcn/B3gat2	3
GO:0015800	acidic amino acid transport	63/21092	0.001941813	0.3155174	0.300119942	Myo6/Snca/Ntrk2/Slc7a11	4
GO:0015711	organic anion transport	404/21092	0.002251239	0.3155174	0.300119942	Slc25a29/Slc17a5/Myo6/Snca/Slc6a5/Slc4a4/Ntrk2/Slc7a11/Atp8a1/Tmem30a	10
GO:0006855	drug transmembrane transport	66/21092	0.002304207	0.3155174	0.300119942	Slc25a29/Slc6a5/Slc7a11/Tmem30a	4
GO:0051648	vesicle localization	283/21092	0.00275487	0.3155174	0.300119942	Myo5c/1190002N15Rik/Syt12/Myo6/Snca/Magi2/Dgki/Dctn1	8
GO:0098739	import across plasma membrane	114/21092	0.002763843	0.3155174	0.300119942	Slc6a5/Hcn2/Slc7a11/Hfe/Kcnj2	5
GO:0014047	glutamate secretion	34/21092	0.002871685	0.3155174	0.300119942	Myo6/Snca/Ntrk2	3
GO:0007612	learning	168/21092	0.002988362	0.3155174	0.300119942	Grin2a/Dgki/Ntrk2/Slc7a11/Atp8a1/Asic1	6
GO:0006826	iron ion transport	35/21092	0.003121703	0.3155174	0.300119942	Trf/Heph/Hfe	3
GO:1990089	response to nerve growth factor	35/21092	0.003121703	0.3155174	0.300119942	Magi2/Tmem108/Ntrk2	3
GO:1990090	cellular response to nerve growth factor stimulus	35/21092	0.003121703	0.3155174	0.300119942	Magi2/Tmem108/Ntrk2	3
GO:0043950	positive regulation of cAMP-mediated signaling	36/21092	0.003384682	0.3155174	0.300119942	Gpr62/Mrap2/Cgas	3
GO:1902475	L-alpha-amino acid transmembrane transport	37/21092	0.003660829	0.3155174	0.300119942	Slc25a29/Slc6a5/Slc7a11	3
GO:0042983	amyloid precursor protein biosynthetic process	11/21092	0.003664453	0.3155174	0.300119942	Necab1/Aatf	2
GO:0042984	regulation of amyloid precursor protein biosynthetic process	11/21092	0.003664453	0.3155174	0.300119942	Necab1/Aatf	2
GO:0106072	negative regulation of adenylate cyclase-activating G protein-coupled receptor signaling pathway	11/21092	0.003664453	0.3155174	0.300119942	Mrap2/Gnai2	2
GO:0014065	phosphatidylinositol 3-kinase signaling	122/21092	0.003699283	0.3155174	0.300119942	1190002N15Rik/Dcn/Agap2/Nyap2/Ntrk2	5
GO:1905475	regulation of protein localization to membrane	180/21092	0.004190431	0.3155174	0.300119942	Mrap2/Magi2/Slc7a11/Cemip/Gpc6/Lypd1	6
GO:0042762	regulation of sulfur metabolic process	12/21092	0.004373164	0.3155174	0.300119942	Snca/Slc7a11	2
GO:0071281	cellular response to iron ion	12/21092	0.004373164	0.3155174	0.300119942	Trf/Hfe	2

GO:0006836	neurotransmitter transport	306/21092	0.004413778	0.3155174	0.300119942	Syt12/Myo6/Snca/Slc6a5/Dgki/Ntrk2/Slc7a11/Asic1	8
GO:0006835	dicarboxylic acid transport	79/21092	0.004416017	0.3155174	0.300119942	Myo6/Snca/Ntrk2/Slc7a11	4
GO:0050805	negative regulation of synaptic transmission	79/21092	0.004416017	0.3155174	0.300119942	Gnai2/Snca/Dgki/Asic1	4
GO:0010559	regulation of glycoprotein biosynthetic process	40/21092	0.004570232	0.3155174	0.300119942	Pxylp1/Necab1/Aatf	3
GO:0001505	regulation of neurotransmitter levels	376/21092	0.004597278	0.3155174	0.300119942	Grin2a/Syt12/Myo6/Snca/Slc6a5/Dgki/Ntrk2/Slc7a11/Asic1	9
GO:0051588	regulation of neurotransmitter transport	184/21092	0.004661041	0.3155174	0.300119942	Syt12/Myo6/Snca/Dgki/Ntrk2/Asic1	6
GO:0030203	glycosaminoglycan metabolic process	81/21092	0.004827159	0.3155174	0.300119942	Pxylp1/Dcn/Itih5/Cemip	4
GO:0038179	neurotrophin signaling pathway	41/21092	0.004900965	0.3155174	0.300119942	Magi2/Tmem108/Ppp2r5b	3
GO:0009068	aspartate family amino acid catabolic process	13/21092	0.005139884	0.3155174	0.300119942	Aass/Ddo	2
GO:0042417	dopamine metabolic process	42/21092	0.005245786	0.3155174	0.300119942	Grin2a/Snca/Sncb	3
GO:0070838	divalent metal ion transport	457/21092	0.005365327	0.3155174	0.300119942	Gnb5/Grin2a/Atp2c1/Gnai2/Snca/Ibtk/Cemip/Kcnj2/Oprd1/Asic1	10
GO:0007269	neurotransmitter secretion	190/21092	0.005438454	0.3155174	0.300119942	Syt12/Myo6/Snca/Dgki/Ntrk2/Asic1	6
GO:0099643	signal release from synapse	190/21092	0.005438454	0.3155174	0.300119942	Syt12/Myo6/Snca/Dgki/Ntrk2/Asic1	6
GO:0060079	excitatory postsynaptic potential	84/21092	0.005490883	0.3155174	0.300119942	Grin2a/Snca/Tmem108/Dgki	4
GO:0072511	divalent inorganic cation transport	460/21092	0.005610938	0.3155174	0.300119942	Gnb5/Grin2a/Atp2c1/Gnai2/Snca/Ibtk/Cemip/Kcnj2/Oprd1/Asic1	10
GO:0060732	positive regulation of inositol phosphate biosynthetic process	14/21092	0.005963602	0.320444194	0.304806305	Plcd1/Snca	2
GO:0106070	regulation of adenylate cyclase-activating G protein-coupled receptor signaling pathway	14/21092	0.005963602	0.320444194	0.304806305	Mrap2/Gnai2	2
GO:1903018	regulation of glycoprotein metabolic process	45/21092	0.006366345	0.327535356	0.311551413	Pxylp1/Necab1/Aatf	3
GO:0050807	regulation of synapse organization	259/21092	0.006366485	0.327535356	0.311551413	Camkv/Zfp804a/Snca/Magi2/Dctn1/Ntrk2/Slc7a11	7
GO:0000041	transition metal ion transport	89/21092	0.006727861	0.328072847	0.312062675	Trf/Atp2c1/Heph/Hfe	4

GO:0010642	negative regulation of platelet-derived growth factor receptor signaling pathway	15/21092	0.006843317	0.328072847	0.312062675	Apod/Snca	2
GO:0097286	iron ion import	15/21092	0.006843317	0.328072847	0.312062675	Trf/Hfe	2
GO:0051650	establishment of vesicle localization	265/21092	0.00718333	0.328072847	0.312062675	Myo5c/1190002N15Rik/Syt12/Myo6/Snca/Dgki/Dctn1	7
GO:0001504	neurotransmitter uptake	47/21092	0.007186592	0.328072847	0.312062675	Snca/Slc6a5/Slc7a11	3
GO:0014066	regulation of phosphatidylinositol 3-kinase signaling	91/21092	0.007270244	0.328072847	0.312062675	1190002N15Rik/Dcn/Agap2/Ntrk2	4
GO:0050803	regulation of synapse structure or activity	266/21092	0.007326689	0.328072847	0.312062675	Camkv/Zfp804a/Snca/Magi2/Dctn1/Ntrk2/Slc7a11	7
GO:0098696	regulation of neurotransmitter receptor localization to postsynaptic specialization membrane	16/21092	0.007778044	0.331220927	0.315057127	Magi2/Gpc6	2
GO:0006816	calcium ion transport	409/21092	0.007827052	0.331220927	0.315057127	Gnb5/Grin2a/Atp2c1/Gnai2/Snca/Ibtk/Cemip/Oprd1/Asic1	9
GO:0048015	phosphatidylinositol-mediated signaling	146/21092	0.007844937	0.331220927	0.315057127	1190002N15Rik/Dcn/Agap2/Nyap2/Ntrk2	5
GO:0070588	calcium ion transmembrane transport	271/21092	0.008075514	0.331220927	0.315057127	Gnb5/Grin2a/Atp2c1/Snca/Ibtk/Cemip/Asic1	7
GO:0099565	chemical synaptic transmission, postsynaptic	94/21092	0.008136677	0.331220927	0.315057127	Grin2a/Snca/Tmem108/Dgki	4
GO:0046916	cellular transition metal ion homeostasis	95/21092	0.008439869	0.331220927	0.315057127	Trf/Atp2c1/Heph/Hfe	4
GO:0048017	inositol lipid-mediated signaling	149/21092	0.008525244	0.331220927	0.315057127	1190002N15Rik/Dcn/Agap2/Nyap2/Ntrk2	5
GO:0015893	drug transport	210/21092	0.008719811	0.331220927	0.315057127	Slc25a29/Syt12/Snca/Slc6a5/Slc7a11/Tmem30a	6
GO:0010919	regulation of inositol phosphate biosynthetic process	17/21092	0.008766807	0.331220927	0.315057127	Plcd1/Snca	2
GO:0051386	regulation of neurotrophin TRK receptor signaling pathway	17/21092	0.008766807	0.331220927	0.315057127	Tmem108/Ppp2r5b	2
GO:0071248	cellular response to metal ion	151/21092	0.009000841	0.334046503	0.317744812	Trf/Grin2a/Syt12/Snca/Hfe	5
GO:0007528	neuromuscular junction development	52/21092	0.009500864	0.334046503	0.317744812	Col4a5/Dctn1/Ntrk2	3

GO:0006898	receptor-mediated endocytosis	214/21092	0.009516311	0.334046503	0.317744812	Trf/Myo6/Snca/Magi2/Tmem108/Hfe	6
GO:0099072	regulation of postsynaptic membrane neurotransmitter receptor levels	99/21092	0.009726387	0.334046503	0.317744812	Myo6/Magi2/Gpc6/Dlg3	4
GO:0002710	negative regulation of T cell mediated immunity	18/21092	0.009808644	0.334046503	0.317744812	Il20rb/Hfe	2
GO:0010752	regulation of cGMP-mediated signaling	18/21092	0.009808644	0.334046503	0.317744812	Pde10a/Cgas	2
GO:0050650	chondroitin sulfate proteoglycan biosynthetic process	18/21092	0.009808644	0.334046503	0.317744812	Pxylp1/B3gat2	2
GO:0006879	cellular iron ion homeostasis	54/21092	0.010534313	0.353777329	0.336512761	Trf/Heph/Hfe	3
GO:0001975	response to amphetamine	19/21092	0.010902606	0.355799583	0.338436328	Grin2a/Asic1	2
GO:0048260	positive regulation of receptor-mediated endocytosis	55/21092	0.011074497	0.355799583	0.338436328	Trf/Magi2/Hfe	3
GO:0014075	response to amine	20/21092	0.012047753	0.355799583	0.338436328	Grin2a/Asic1	2
GO:0006584	catecholamine metabolic process	57/21092	0.012202184	0.355799583	0.338436328	Grin2a/Snca/Sncb	3
GO:0009712	catechol-containing compound metabolic process	57/21092	0.012202184	0.355799583	0.338436328	Grin2a/Snca/Sncb	3
GO:0045744	negative regulation of G protein-coupled receptor signaling pathway	57/21092	0.012202184	0.355799583	0.338436328	Mrap2/Gnai2/Snca	3
GO:1903510	mucopolysaccharide metabolic process	57/21092	0.012202184	0.355799583	0.338436328	Dcn/Itih5/Cemip	3
GO:1903539	protein localization to postsynaptic membrane	57/21092	0.012202184	0.355799583	0.338436328	Grin2a/Magi2/Gpc6	3
GO:0018958	phenol-containing compound metabolic process	106/21092	0.012271653	0.355799583	0.338436328	Grin2a/Snca/Sncb/Slc7a11	4
GO:0016311	dephosphorylation	367/21092	0.012532008	0.355799583	0.338436328	Ppm1m/Gnai2/Magi2/Ppm1j/Dusp7/Dlg3/Ppp2r5b/Sfi1	8
GO:0099504	synaptic vesicle cycle	228/21092	0.01271371	0.355799583	0.338436328	Syt12/Myo6/Snca/Magi2/Sncb/Dgki	6
GO:0046942	carboxylic acid transport	297/21092	0.012907908	0.355799583	0.338436328	Slc25a29/Slc17a5/Myo6/Snca/Slc6a5/Ntrk2/Slc7a11	7
GO:0015849	organic acid transport	298/21092	0.013127784	0.355799583	0.338436328	Slc25a29/Slc17a5/Myo6/Snca/Slc6a5/Ntrk2/Slc7a11	7

GO:0003433	chondrocyte development involved in endochondral bone morphogenesis	21/21092	0.013243161	0.355799583	0.338436328	Poc1a/Vit	2
GO:0095500	acetylcholine receptor signaling pathway	21/21092	0.013243161	0.355799583	0.338436328	Gnai2/Lypd1	2
GO:1903831	signal transduction involved in cellular response to ammonium ion	21/21092	0.013243161	0.355799583	0.338436328	Gnai2/Lypd1	2
GO:1905144	response to acetylcholine	21/21092	0.013243161	0.355799583	0.338436328	Gnai2/Lypd1	2
GO:1905145	cellular response to acetylcholine	21/21092	0.013243161	0.355799583	0.338436328	Gnai2/Lypd1	2
GO:0015807	L-amino acid transport	59/21092	0.013393424	0.355882404	0.338515107	Slc25a29/Slc6a5/Slc7a11	3
GO:0060078	regulation of postsynaptic membrane potential	111/21092	0.014328207	0.368755538	0.350760024	Grin2a/Snca/Tmem108/Dgki	4
GO:0003418	growth plate cartilage chondrocyte differentiation	22/21092	0.014487914	0.368755538	0.350760024	Poc1a/Vit	2
GO:0015874	norepinephrine transport	22/21092	0.014487914	0.368755538	0.350760024	Plcd1/Snca	2
GO:1902683	regulation of receptor localization to synapse	22/21092	0.014487914	0.368755538	0.350760024	Magi2/Gpc6	2
GO:0015672	monovalent inorganic cation transport	458/21092	0.015501344	0.378545307	0.360072046	Atp6v1g2/Kcnk9/Slc4a4/Hcn2/Kcnk2/Cyc1/Kcnh3/Kcnj2/Asic1	9
GO:1903828	negative regulation of cellular protein localization	114/21092	0.015660773	0.378545307	0.360072046	Dclk3/Mrap2/Apod/Lypd1	4
GO:1903077	negative regulation of protein localization to plasma membrane	23/21092	0.015781111	0.378545307	0.360072046	Mrap2/Lypd1	2
GO:0006801	superoxide metabolic process	63/21092	0.015968412	0.378545307	0.360072046	Cyb5r4/Aatf/Gnai2	3
GO:0030330	DNA damage response, signal transduction by p53 class mediator	63/21092	0.015968412	0.378545307	0.360072046	Pcbp4/Myo6/E2f7	3
GO:0042440	pigment metabolic process	63/21092	0.015968412	0.378545307	0.360072046	Cyb5r4/Alas1/Slc7a11	3
GO:0043113	receptor clustering	63/21092	0.015968412	0.378545307	0.360072046	Magi2/Slc7a11/Dlg3	3
GO:0031346	positive regulation of cell projection organization	462/21092	0.016310554	0.379822529	0.361286939	Twf2/Mns1/Zfp804a/Magi2/Ntrk2/Metrn/Efemp1/Ppp2r5b/Tmem30a	9
GO:0006470	protein dephosphorylation	242/21092	0.016599944	0.379822529	0.361286939	Ppm1m/Gnai2/Magi2/Ppm1j/Dusp7/Ppp2r5b	6

GO:0010640	regulation of platelet-derived growth factor receptor signaling pathway	24/21092	0.017121859	0.379822529	0.361286939	Apod/Snca	2
GO:0032928	regulation of superoxide anion generation	24/21092	0.017121859	0.379822529	0.361286939	Aatf/Gnai2	2
GO:0035384	thioester biosynthetic process	24/21092	0.017121859	0.379822529	0.361286939	Snca/Dlat	2
GO:0071616	acyl-CoA biosynthetic process	24/21092	0.017121859	0.379822529	0.361286939	Snca/Dlat	2
GO:0098810	neurotransmitter reuptake	24/21092	0.017121859	0.379822529	0.361286939	Snca/Slc6a5	2
GO:0051193	regulation of cofactor metabolic process	65/21092	0.017352887	0.381448013	0.362833097	Me1/Snca/Slc7a11	3
GO:0019933	cAMP-mediated signaling	179/21092	0.017689275	0.385339341	0.366534526	Gpr62/Pde10a/Mrap2/Gnai2/Cgas	5
GO:0099010	modification of postsynaptic structure	25/21092	0.01850928	0.38858238	0.369619302	Filip1/Camkv	2
GO:0120033	negative regulation of plasma membrane bounded cell projection assembly	25/21092	0.01850928	0.38858238	0.369619302	Slit2/Cdk10	2
GO:1902473	regulation of protein localization to synapse	25/21092	0.01850928	0.38858238	0.369619302	Magi2/Gpc6	2
GO:1904376	negative regulation of protein localization to cell periphery	25/21092	0.01850928	0.38858238	0.369619302	Mrap2/Lypd1	2
GO:0006029	proteoglycan metabolic process	67/21092	0.018802373	0.38858238	0.369619302	Pxylp1/Dcn/B3gat2	3
GO:0014068	positive regulation of phosphatidylinositol 3-kinase signaling	67/21092	0.018802373	0.38858238	0.369619302	Dcn/Agap2/Ntrk2	3
GO:0051668	localization within membrane	183/21092	0.019265243	0.394676759	0.375416272	Myo6/Magi2/Slc7a11/Gpc6/Dlg3	5
GO:0048168	regulation of neuronal synaptic plasticity	68/21092	0.019551554	0.394676759	0.375416272	Grin2a/Rasgrf1/Snca	3
GO:0010921	regulation of phosphatase activity	122/21092	0.019586936	0.394676759	0.375416272	Gnai2/Magi2/Dlg3/Sfi1	4
GO:0030212	hyaluronan metabolic process	26/21092	0.019942505	0.395253915	0.375965263	Itih5/Cemip	2
GO:0045332	phospholipid translocation	26/21092	0.019942505	0.395253915	0.375965263	Atp8a1/Tmem30a	2
GO:0031589	cell-substrate adhesion	325/21092	0.020133669	0.395798462	0.376483235	Rpl29/Tdgf1/Apod/Sned1/Spp1/Ecm2/Vit	7
GO:0010811	positive regulation of cell-substrate adhesion	125/21092	0.021202155	0.402000363	0.382382479	Tdgf1/Spp1/Ecm2/Vit	4

GO:0006026	aminoglycan catabolic process	27/21092	0.021420677	0.402000363	0.382382479	Chil5/Cemip	2
GO:0034204	lipid translocation	27/21092	0.021420677	0.402000363	0.382382479	Atp8a1/Tmem30a	2
GO:1902932	positive regulation of alcohol biosynthetic process	27/21092	0.021420677	0.402000363	0.382382479	Plcd1/Snca	2
GO:0007631	feeding behavior	126/21092	0.021758176	0.402000363	0.382382479	Hcrtr2/Mrap2/Ntrk2/Oprd1	4
GO:0050433	regulation of catecholamine secretion	71/21092	0.021897022	0.402000363	0.382382479	Plcd1/Syt12/Snca	3
GO:0097120	receptor localization to synapse	71/21092	0.021897022	0.402000363	0.382382479	Magi2/Gpc6/Dlg3	3
GO:0042177	negative regulation of protein catabolic process	127/21092	0.022323059	0.402000363	0.382382479	Grin2a/Agap2/Snca/Hfe	4
GO:0097553	calcium ion transmembrane import into cytosol	127/21092	0.022323059	0.402000363	0.382382479	Grin2a/Snca/Ibtk/Cemip	4
GO:0010959	regulation of metal ion transport	410/21092	0.022737662	0.402000363	0.382382479	Gnb5/Trf/Gnai2/Snca/Hfe/Cemip/Kcnj2/Oprd1	8
GO:0003413	chondrocyte differentiation involved in endochondral bone morphogenesis	28/21092	0.022942949	0.402000363	0.382382479	Poc1a/Vit	2
GO:0045780	positive regulation of bone resorption	28/21092	0.022942949	0.402000363	0.382382479	Trf/Spp1	2
GO:0046852	positive regulation of bone remodeling	28/21092	0.022942949	0.402000363	0.382382479	Trf/Spp1	2
GO:0089718	amino acid import across plasma membrane	28/21092	0.022942949	0.402000363	0.382382479	Slc6a5/Slc7a11	2
GO:1902668	negative regulation of axon guidance	28/21092	0.022942949	0.402000363	0.382382479	Slit2/Sema6b	2
GO:0097479	synaptic vesicle localization	192/21092	0.023141781	0.402567096	0.382921555	Syt12/Myo6/Snca/Magi2/Dgki	5
GO:0071804	cellular potassium ion transport	193/21092	0.023601378	0.40313958	0.383466102	Kcnk9/Hcn2/Kcnk2/Kcnh3/Kcnj2	5
GO:0071805	potassium ion transmembrane transport	193/21092	0.023601378	0.40313958	0.383466102	Kcnk9/Hcn2/Kcnk2/Kcnh3/Kcnj2	5
GO:0055076	transition metal ion homeostasis	130/21092	0.024071178	0.40313958	0.383466102	Trf/Atp2c1/Heph/Hfe	4
GO:0003333	amino acid transmembrane transport	74/21092	0.024389498	0.40313958	0.383466102	Slc25a29/Slc6a5/Slc7a11	3
GO:0032272	negative regulation of protein polymerization	74/21092	0.024389498	0.40313958	0.383466102	Twf2/Snca/Slit2	3
GO:0050432	catecholamine secretion	74/21092	0.024389498	0.40313958	0.383466102	Plcd1/Syt12/Snca	3

GO:0042168	heme metabolic process	29/21092	0.024508486	0.40313958	0.383466102	Cyb5r4/Alas1	2
GO:0051589	negative regulation of neurotransmitter transport	29/21092	0.024508486	0.40313958	0.383466102	Snca/Asic1	2
GO:0051881	regulation of mitochondrial membrane potential	75/21092	0.025252981	0.412579103	0.392444969	Dcn/Tusc2/Oprd1	3
GO:0043112	receptor metabolic process	198/21092	0.025987788	0.417990687	0.397592465	Myo6/Snca/Magi2/Pik3r4/Pheta1	5
GO:0010038	response to metal ion	269/21092	0.026268205	0.417990687	0.397592465	Trf/Grin2a/Plcd1/Syt12/Snca/Hfe	6
GO:0090287	regulation of cellular response to growth factor stimulus	269/21092	0.026268205	0.417990687	0.397592465	Tdgf1/Dcn/Tmem108/Slit2/Bcl9l/Ppp2r5b	6
GO:1904375	regulation of protein localization to cell periphery	134/21092	0.0265278	0.417990687	0.397592465	Mrap2/Magi2/Gpc6/Lypd1	4
GO:0044272	sulfur compound biosynthetic process	77/21092	0.027028868	0.417990687	0.397592465	Snca/Slc7a11/Dlat	3
GO:0032958	inositol phosphate biosynthetic process	31/21092	0.027766067	0.417990687	0.397592465	Plcd1/Snca	2
GO:0045920	negative regulation of exocytosis	31/21092	0.027766067	0.417990687	0.397592465	Gnai2/Snca	2
GO:0099563	modification of synaptic structure	31/21092	0.027766067	0.417990687	0.397592465	Filip1/Camkv	2
GO:0099633	protein localization to postsynaptic specialization membrane	31/21092	0.027766067	0.417990687	0.397592465	Magi2/Gpc6	2
GO:0099645	neurotransmitter receptor localization to postsynaptic specialization membrane	31/21092	0.027766067	0.417990687	0.397592465	Magi2/Gpc6	2
GO:1905476	negative regulation of protein localization to membrane	31/21092	0.027766067	0.417990687	0.397592465	Mrap2/Lypd1	2
GO:0048167	regulation of synaptic plasticity	348/21092	0.027881899	0.417990687	0.397592465	Grin2a/Rasgrf1/Syt12/Myo6/Snca/Dgki/Ntrk2	7
GO:0010923	negative regulation of phosphatase activity	78/21092	0.027941239	0.417990687	0.397592465	Gnai2/Dlg3/Sfi1	3
GO:0050770	regulation of axonogenesis	202/21092	0.028004339	0.417990687	0.397592465	Twf2/Ntrk2/Metrn/Slit2/Sema6b	5
GO:0043090	amino acid import	32/21092	0.029456493	0.434303664	0.413109358	Slc6a5/Slc7a11	2
GO:0048011	neurotrophin TRK receptor signaling pathway	32/21092	0.029456493	0.434303664	0.413109358	Tmem108/Ppp2r5b	2

GO:0098659	inorganic cation import across plasma membrane	81/21092	0.030775784	0.438771595	0.417359251	Hcn2/Hfe/Kcnj2	3
GO:0099587	inorganic ion import across plasma membrane	81/21092	0.030775784	0.438771595	0.417359251	Hcn2/Hfe/Kcnj2	3
GO:0031333	negative regulation of protein complex assembly	141/21092	0.031176982	0.438771595	0.417359251	Twf2/Snca/Slit2/Oprd1	4
GO:0090288	negative regulation of cellular response to growth factor stimulus	141/21092	0.031176982	0.438771595	0.417359251	Tdgf1/Dcn/Slit2/Bcl9l	4
GO:0032733	positive regulation of interleukin-10 production	33/21092	0.03118695	0.438771595	0.417359251	Il20rb/Tusc2	2
GO:0042554	superoxide anion generation	33/21092	0.03118695	0.438771595	0.417359251	Aatf/Gnai2	2
GO:1900181	negative regulation of protein localization to nucleus	33/21092	0.03118695	0.438771595	0.417359251	Dclk3/Apod	2
GO:0010810	regulation of cell-substrate adhesion	208/21092	0.031211214	0.438771595	0.417359251	Tdgf1/Apod/Spp1/Ecm2/Vit	5
GO:0010976	positive regulation of neuron projection development	357/21092	0.031400012	0.438874154	0.417456805	Twf2/Zfp804a/Magi2/Ntrk2/Metrn/Ppp2r5b/Tmem30a	7
GO:0007611	learning or memory	283/21092	0.032509909	0.445191004	0.423465388	Grin2a/Dgki/Ntrk2/Slc7a11/Atp8a1/Asic1	6
GO:0019935	cyclic-nucleotide-mediated signaling	211/21092	0.032897621	0.445191004	0.423465388	Gpr62/Pde10a/Mrap2/Gnai2/Cgas	5
GO:0007271	synaptic transmission, cholinergic	34/21092	0.032956654	0.445191004	0.423465388	Lama2/Lypd1	2
GO:0010737	protein kinase A signaling	34/21092	0.032956654	0.445191004	0.423465388	Pde10a/Lcp1	2
GO:0090322	regulation of superoxide metabolic process	34/21092	0.032956654	0.445191004	0.423465388	Aatf/Gnai2	2
GO:0097366	response to bronchodilator	34/21092	0.032956654	0.445191004	0.423465388	Grin2a/Asic1	2
GO:0010591	regulation of lamellipodium assembly	35/21092	0.034764833	0.459351728	0.43693506	Twf2/Slit2	2
GO:0033866	nucleoside bisphosphate biosynthetic process	35/21092	0.034764833	0.459351728	0.43693506	Snca/Dlat	2
GO:0034030	ribonucleoside bisphosphate biosynthetic process	35/21092	0.034764833	0.459351728	0.43693506	Snca/Dlat	2
GO:0034033	purine nucleoside bisphosphate biosynthetic process	35/21092	0.034764833	0.459351728	0.43693506	Snca/Dlat	2
GO:0042490	mechanoreceptor differentiation	86/21092	0.035822894	0.460779532	0.438293186	Myo6/Cthrc1/Ntrk2	3

GO:0060402	calcium ion transport into cytosol	148/21092	0.036276764	0.460779532	0.438293186	Grin2a/Snca/Ibtk/Cemip	4
GO:0071241	cellular response to inorganic substance	217/21092	0.036438489	0.460779532	0.438293186	Trf/Grin2a/Syt12/Snca/Hfe	5
GO:0003417	growth plate cartilage development	36/21092	0.036610724	0.460779532	0.438293186	Poc1a/Vit	2
GO:0006739	NADP metabolic process	36/21092	0.036610724	0.460779532	0.438293186	Me1/Cyb5r4	2
GO:0033238	regulation of cellular amine metabolic process	36/21092	0.036610724	0.460779532	0.438293186	Snca/Slc7a11	2
GO:0050919	negative chemotaxis	36/21092	0.036610724	0.460779532	0.438293186	Slit2/Sema6b	2
GO:0051181	cofactor transport	36/21092	0.036610724	0.460779532	0.438293186	Slc25a29/Slc7a11	2
GO:0072524	pyridine-containing compound metabolic process	149/21092	0.037042323	0.460779532	0.438293186	Me1/Cyb5r4/Pnpo/Dlat	4
GO:1901615	organic hydroxy compound metabolic process	453/21092	0.037758467	0.460779532	0.438293186	Grin2a/Pnpo/Plcd1/Snca/Sncb/Cyp11a1/Slc7a11/Haghl	8
GO:0042737	drug catabolic process	150/21092	0.037817159	0.460779532	0.438293186	Aass/Snca/Chil5/Ctsh	4
GO:0035418	protein localization to synapse	88/21092	0.037953804	0.460779532	0.438293186	Grin2a/Magi2/Gpc6	3
GO:0055072	iron ion homeostasis	88/21092	0.037953804	0.460779532	0.438293186	Trf/Heph/Hfe	3
GO:0006778	porphyrin-containing compound metabolic process	37/21092	0.038493575	0.460779532	0.438293186	Cyb5r4/Alas1	2
GO:0009311	oligosaccharide metabolic process	37/21092	0.038493575	0.460779532	0.438293186	B3galt1/Man2c1	2
GO:0032620	interleukin-17 production	37/21092	0.038493575	0.460779532	0.438293186	Tusc2/Rorc	2
GO:0048169	regulation of long-term neuronal synaptic plasticity	37/21092	0.038493575	0.460779532	0.438293186	Grin2a/Snca	2
GO:0048846	axon extension involved in axon guidance	37/21092	0.038493575	0.460779532	0.438293186	Slit2/Sema6b	2
GO:1902284	neuron projection extension involved in neuron projection guidance	37/21092	0.038493575	0.460779532	0.438293186	Slit2/Sema6b	2
GO:0019318	hexose metabolic process	221/21092	0.03892471	0.462775342	0.440191599	Apod/Glb1/Dlat/Man2c1/Rorc	5
GO:0051937	catecholamine transport	89/21092	0.039043081	0.462775342	0.440191599	Plcd1/Syt12/Snca	3

GO:0042770	signal transduction in response to DNA damage	90/21092	0.040148167	0.47355253	0.450442853	Pcbp4/Myo6/E2f7	3
GO:0002686	negative regulation of leukocyte migration	38/21092	0.040412644	0.474358121	0.45120913	Apod/Slit2	2
GO:0035305	negative regulation of dephosphorylation	91/21092	0.041269006	0.482069834	0.458544507	Gnai2/Dlg3/Sfi1	3
GO:0001881	receptor recycling	39/21092	0.042367197	0.484971177	0.461304263	Snca/Pheta1	2
GO:0002823	negative regulation of adaptive immune response based on somatic recombination of immune receptors built from immunoglobulin superfamily domains	39/21092	0.042367197	0.484971177	0.461304263	Il20rb/Hfe	2
GO:0019934	cGMP-mediated signaling	39/21092	0.042367197	0.484971177	0.461304263	Pde10a/Cgas	2
GO:0045807	positive regulation of endocytosis	157/21092	0.043501024	0.484971177	0.461304263	Trf/Snca/Magi2/Hfe	4
GO:0051188	cofactor biosynthetic process	228/21092	0.043519857	0.484971177	0.461304263	Pnpo/Snca/Alas1/Slc7a11/Dlat	5
GO:0033674	positive regulation of kinase activity	467/21092	0.043822185	0.484971177	0.461304263	Tdgf1/Trf/Map3k19/Agap2/Snca/Pik3r4/Cemip/Dlg3	8
GO:1990573	potassium ion import across plasma membrane	40/21092	0.044356511	0.484971177	0.461304263	Hcn2/Kcnj2	2
GO:0009100	glycoprotein metabolic process	308/21092	0.045902873	0.484971177	0.461304263	Pxylp1/Necab1/Dcn/B3gat2/Aatf/B3galt1	6
GO:0044282	small molecule catabolic process	308/21092	0.045902873	0.484971177	0.461304263	Acad11/Aass/Glb1/Cemip/Ddo/Haghl	6
GO:0007200	phospholipase C-activating G protein-coupled receptor signaling pathway	95/21092	0.045908647	0.484971177	0.461304263	Hcrtr2/Plcd1/Oprd1	3
GO:0060359	response to ammonium ion	95/21092	0.045908647	0.484971177	0.461304263	Gnai2/Snca/Lypd1	3
GO:0060401	cytosolic calcium ion transport	160/21092	0.04607626	0.484971177	0.461304263	Grin2a/Snca/Ibtk/Cemip	4
GO:0034105	positive regulation of tissue remodeling	41/21092	0.046379872	0.484971177	0.461304263	Trf/Spp1	2
GO:0051281	positive regulation of release of sequestered calcium ion into cytosol	41/21092	0.046379872	0.484971177	0.461304263	Snca/Cemip	2
GO:0043648	dicarboxylic acid metabolic process	96/21092	0.047107301	0.484971177	0.461304263	Me1/Slc7a11/Ddo	3
GO:0006813	potassium ion transport	234/21092	0.047707806	0.484971177	0.461304263	Kcnk9/Hcn2/Kcnk2/Kcnh3/Kcnj2	5

GO:1901215	negative regulation of neuron death	234/21092	0.047707806	0.484971177	0.461304263	Agap2/Snca/Sncb/Ntrk2/Slc7a11	5
GO:0006732	coenzyme metabolic process	311/21092	0.047709981	0.484971177	0.461304263	Me1/Cyb5r4/Pnpo/Snca/Folh1/Dlat	6
GO:0051656	establishment of organelle localization	476/21092	0.048042656	0.484971177	0.461304263	Mlh1/Myo5c/1190002N15Rik/Syt12/Myo6/Snca/Dgki/Dctn1	8
GO:0006140	regulation of nucleotide metabolic process	97/21092	0.048321314	0.484971177	0.461304263	Guca1b/Me1/Snca	3
GO:0002820	negative regulation of adaptive immune response	42/21092	0.048436576	0.484971177	0.461304263	Il20rb/Hfe	2
GO:0009066	aspartate family amino acid metabolic process	42/21092	0.048436576	0.484971177	0.461304263	Aass/Ddo	2
GO:0031644	regulation of neurological system process	163/21092	0.048734935	0.484971177	0.461304263	Trf/Grin2a/Tmem108/Oprd1	4
GO:0035303	regulation of dephosphorylation	163/21092	0.048734935	0.484971177	0.461304263	Gnai2/Magi2/Dlg3/Sfi1	4
GO:0051186	cofactor metabolic process	478/21092	0.049015382	0.484971177	0.461304263	Me1/Cyb5r4/Pnpo/Snca/Alas1/Slc7a11/Folh1/Dlat	8
GO:0050772	positive regulation of axonogenesis	98/21092	0.049550611	0.484971177	0.461304263	Twf2/Ntrk2/Metrn	3

Supp. Table S14. List of Biological Processes (BP) at FDR 5% - hippocampus

GO ID	Description	BgRatio	pvalue	p.adjust	qvalue	geneID	Count
GO:0031282	regulation of guanylate cyclase activity	10/21092	7.96591E-06	0.012386993	0.011663772	Guca1b/Guca1a/Nos1	3
GO:0006140	regulation of nucleotide metabolic process	97/21092	5.08445E-05	0.03953156	0.03722349	Guca1b/Me1/Guca1a/Nos1/Cox7a2	5
GO:1900542	regulation of purine nucleotide metabolic process	93/21092	0.000596112	0.140031904	0.13185607	Guca1b/Guca1a/Nos1/Cox7a2	4
GO:0031279	regulation of cyclase activity	41/21092	0.000645175	0.140031904	0.13185607	Guca1b/Guca1a/Nos1	3
GO:0010753	positive regulation of cGMP-mediated signaling	10/21092	0.00074076	0.140031904	0.13185607	Cgas/Guca1a	2
GO:0051339	regulation of lyase activity	43/21092	0.000742487	0.140031904	0.13185607	Guca1b/Guca1a/Nos1	3
GO:0007128	meiotic prophase I	11/21092	0.000902946	0.140031904	0.13185607	Mlh1/Piwil2	2
GO:0051324	prophase	11/21092	0.000902946	0.140031904	0.13185607	Mlh1/Piwil2	2
GO:0061448	connective tissue development	274/21092	0.000953328	0.140031904	0.13185607	Poc1a/Adamts7/Col12a1/Arid5b/Sox6/Hes5	6

GO:0000279	M phase	12/21092	0.001080632	0.140031904	0.13185607	Mlh1/Piwil2	2
GO:0098762	meiotic cell cycle phase	12/21092	0.001080632	0.140031904	0.13185607	Mlh1/Piwil2	2
GO:0098764	meiosis I cell cycle phase	12/21092	0.001080632	0.140031904	0.13185607	Mlh1/Piwil2	2
GO:0045604	regulation of epidermal cell differentiation	52/21092	0.00129456	0.15112002	0.1422968	Cd109/Ptch2/Hes5	3
GO:0002062	chondrocyte differentiation	116/21092	0.001360566	0.15112002	0.1422968	Poc1a/Adamts7/Col12a1/Sox6	4
GO:0021781	glial cell fate commitment	14/21092	0.001481992	0.153633144	0.144663194	Sox6/Hes5	2
GO:0051216	cartilage development	205/21092	0.001600836	0.155581213	0.146497524	Poc1a/Adamts7/Col12a1/Sox6/Hes5	5
GO:0010752	regulation of cGMP-mediated signaling	18/21092	0.002465137	0.198716323	0.187114169	Cgas/Guca1a	2
GO:0030808	regulation of nucleotide biosynthetic process	66/21092	0.002571542	0.198716323	0.187114169	Guca1b/Guca1a/Nos1	3
GO:1900371	regulation of purine nucleotide biosynthetic process	66/21092	0.002571542	0.198716323	0.187114169	Guca1b/Guca1a/Nos1	3
GO:0062012	regulation of small molecule metabolic process	340/21092	0.002836471	0.198716323	0.187114169	Guca1b/Me1/Elovl5/Guca1a/Nos1/Cox7a2	6
GO:0022403	cell cycle phase	20/21092	0.003044935	0.198716323	0.187114169	Mlh1/Piwil2	2
GO:0031281	positive regulation of cyclase activity	20/21092	0.003044935	0.198716323	0.187114169	Guca1a/Nos1	2
GO:0051349	positive regulation of lyase activity	20/21092	0.003044935	0.198716323	0.187114169	Guca1a/Nos1	2
GO:0007601	visual perception	145/21092	0.003067004	0.198716323	0.187114169	Guca1b/Bfsp2/Wfs1/Guca1a	4
GO:0003433	chondrocyte development involved in endochondral bone morphogenesis	21/21092	0.003356461	0.202224923	0.190417917	Poc1a/Col12a1	2
GO:0050953	sensory perception of light stimulus	149/21092	0.003381253	0.202224923	0.190417917	Guca1b/Bfsp2/Wfs1/Guca1a	4
GO:0003418	growth plate cartilage chondrocyte differentiation	22/21092	0.003682244	0.212069962	0.199688148	Poc1a/Col12a1	2
GO:0045682	regulation of epidermis development	78/21092	0.004126127	0.229147391	0.215768503	Cd109/Ptch2/Hes5	3
GO:1903035	negative regulation of response to wounding	83/21092	0.004910303	0.263293819	0.247921275	Cd109/Klk8/Tmprss6	3
GO:0001822	kidney development	274/21092	0.005544714	0.287401017	0.270620961	Pygo1/Wfs1/Aph1c/Arid5b/Hes5	5

GO:0003413	chondrocyte differentiation involved in endochondral bone morphogenesis	28/21092	0.005929656	0.28974529	0.272828363	Poc1a/Col12a1	2
GO:0000041	transition metal ion transport	89/21092	0.005962604	0.28974529	0.272828363	Atp2c1/Heph/Nectin1	3
GO:0035066	positive regulation of histone acetylation	29/21092	0.006351912	0.299309808	0.281834451	Nos1/Piwil2	2
GO:2000272	negative regulation of signaling receptor activity	30/21092	0.006787485	0.310427604	0.292303129	Crhbp/Socs5	2
GO:0046916	cellular transition metal ion homeostasis	95/21092	0.00714002	0.31256615	0.294316814	Tmprss6/Atp2c1/Heph	3
GO:0007602	phototransduction	31/21092	0.007236258	0.31256615	0.294316814	Guca1b/Guca1a	2
GO:0072001	renal system development	296/21092	0.007626354	0.317723617	0.299173161	Pygo1/Wfs1/Aph1c/Arid5b/Hes5	5
GO:0030810	positive regulation of nucleotide biosynthetic process	33/21092	0.008172955	0.317723617	0.299173161	Guca1a/Nos1	2
GO:1900373	positive regulation of purine nucleotide biosynthetic process	33/21092	0.008172955	0.317723617	0.299173161	Guca1a/Nos1	2
GO:2000758	positive regulation of peptidyl-lysine acetylation	33/21092	0.008172955	0.317723617	0.299173161	Nos1/Piwil2	2
GO:0006826	iron ion transport	35/21092	0.009161101	0.334296956	0.314778857	Heph/Nectin1	2
GO:0006986	response to unfolded protein	106/21092	0.009634673	0.334296956	0.314778857	Wfs1/Hspb1/Hspa1b	3
GO:0003417	growth plate cartilage development	36/21092	0.009674188	0.334296956	0.314778857	Poc1a/Col12a1	2
GO:0006739	NADP metabolic process	36/21092	0.009674188	0.334296956	0.314778857	Me1/Cyb5r4	2
GO:0043950	positive regulation of cAMP-mediated signaling	36/21092	0.009674188	0.334296956	0.314778857	Cgas/Nos1	2
GO:0048821	erythrocyte development	37/21092	0.010199805	0.344797769	0.324666574	Tmod3/Sox6	2
GO:0032102	negative regulation of response to external stimulus	323/21092	0.01085927	0.359280116	0.338303362	Cd109/Klk8/Nt5e/Tmprss6/Socs5	5
GO:0019934	cGMP-mediated signaling	39/21092	0.011288189	0.364902962	0.343597915	Cgas/Guca1a	2
GO:0042326	negative regulation of phosphorylation	461/21092	0.012038113	0.364902962	0.343597915	Cd109/Nos1/Socs5/Nprl2/Hspb1/Ibtk	6
GO:0051701	interaction with host	116/21092	0.012291784	0.364902962	0.343597915	Tusc2/Trim21/Nectin1	3
GO:0042220	response to cocaine	42/21092	0.013012015	0.364902962	0.343597915	Crhbp/Nos1	2

GO:0045981	positive regulation of nucleotide metabolic process	42/21092	0.013012015	0.364902962	0.343597915	Guca1a/Nos1	2
GO:1900544	positive regulation of purine nucleotide metabolic process	42/21092	0.013012015	0.364902962	0.343597915	Guca1a/Nos1	2
GO:1901985	positive regulation of protein acetylation	42/21092	0.013012015	0.364902962	0.343597915	Nos1/Piwil2	2
GO:0001655	urogenital system development	339/21092	0.013163049	0.364902962	0.343597915	Pygo1/Wfs1/Aph1c/Arid5b/Hes5	5
GO:0001709	cell fate determination	43/21092	0.013610528	0.364902962	0.343597915	Ptch2/Hes5	2
GO:0003416	endochondral bone growth	43/21092	0.013610528	0.364902962	0.343597915	Poc1a/Col12a1	2
GO:0043616	keratinocyte proliferation	43/21092	0.013610528	0.364902962	0.343597915	Cd109/Klk8	2
GO:0002063	chondrocyte development	44/21092	0.014220818	0.372266453	0.350531485	Poc1a/Col12a1	2
GO:0072522	purine-containing compound biosynthetic process	227/21092	0.014511589	0.372266453	0.350531485	Guca1b/Nt5e/Guca1a/Nos1	4
GO:0044848	biological phase	45/21092	0.014842778	0.372266453	0.350531485	Mlh1/Piwil2	2
GO:0098868	bone growth	45/21092	0.014842778	0.372266453	0.350531485	Poc1a/Col12a1	2
GO:0030218	erythrocyte differentiation	126/21092	0.015329422	0.378369072	0.3562778	Tmod3/Heph/Sox6	3
GO:0035966	response to topologically incorrect protein	127/21092	0.015654387	0.380352676	0.35814559	Wfs1/Hspb1/Hspa1b	3
GO:0055076	transition metal ion homeostasis	130/21092	0.016652558	0.389337206	0.366605554	Tmprss6/Atp2c1/Heph	3
GO:0031641	regulation of myelination	48/21092	0.016777644	0.389337206	0.366605554	Klk8/Hes5	2
GO:0032330	regulation of chondrocyte differentiation	48/21092	0.016777644	0.389337206	0.366605554	Adamts7/Sox6	2
GO:0060351	cartilage development involved in endochondral bone morphogenesis	51/21092	0.01881383	0.389337206	0.366605554	Poc1a/Col12a1	2
GO:0034101	erythrocyte homeostasis	137/21092	0.019117914	0.389337206	0.366605554	Tmod3/Heph/Sox6	3
GO:0009791	post-embryonic development	138/21092	0.01948573	0.389337206	0.366605554	Pygo1/Arid5b/Sox6	3
GO:0016573	histone acetylation	138/21092	0.01948573	0.389337206	0.366605554	Morf4l1/Nos1/Piwil2	3
GO:0009583	detection of light stimulus	52/21092	0.019514601	0.389337206	0.366605554	Guca1b/Guca1a	2
GO:0006879	cellular iron ion homeostasis	54/21092	0.020948606	0.389337206	0.366605554	Tmprss6/Heph	2

GO:0035065	regulation of histone acetylation	54/21092	0.020948606	0.389337206	0.366605554	Nos1/Piwil2	2
GO:0046718	viral entry into host cell	54/21092	0.020948606	0.389337206	0.366605554	Trim21/Nectin1	2
GO:0061077	chaperone-mediated protein folding	54/21092	0.020948606	0.389337206	0.366605554	Hspb1/Hspa1b	2
GO:0007224	smoothened signaling pathway	143/21092	0.021383537	0.389337206	0.366605554	Ptch2/Scube1/Hes5	3
GO:0018393	internal peptidyl-lysine acetylation	143/21092	0.021383537	0.389337206	0.366605554	Morf4l1/Nos1/Piwil2	3
GO:0006475	internal protein amino acid acetylation	146/21092	0.022569246	0.389337206	0.366605554	Morf4l1/Nos1/Piwil2	3
GO:0030856	regulation of epithelial cell differentiation	146/21092	0.022569246	0.389337206	0.366605554	Cd109/Ptch2/Hes5	3
GO:0062013	positive regulation of small molecule metabolic process	146/21092	0.022569246	0.389337206	0.366605554	Elovl5/Guca1a/Nos1	3
GO:0006457	protein folding	147/21092	0.022972324	0.389337206	0.366605554	Ppib/Hspb1/Hspa1b	3
GO:0030260	entry into host cell	57/21092	0.02317929	0.389337206	0.366605554	Trim21/Nectin1	2
GO:0044409	entry into host	57/21092	0.02317929	0.389337206	0.366605554	Trim21/Nectin1	2
GO:0051806	entry into cell of other organism involved in symbiotic interaction	57/21092	0.02317929	0.389337206	0.366605554	Trim21/Nectin1	2
GO:0051828	entry into other organism involved in symbiotic interaction	57/21092	0.02317929	0.389337206	0.366605554	Trim21/Nectin1	2
GO:0072347	response to anesthetic	57/21092	0.02317929	0.389337206	0.366605554	Crhbp/Nos1	2
GO:1903902	positive regulation of viral life cycle	57/21092	0.02317929	0.389337206	0.366605554	Ppib/Trim21	2
GO:0018394	peptidyl-lysine acetylation	154/21092	0.025903624	0.389337206	0.366605554	Morf4l1/Nos1/Piwil2	3
GO:0051926	negative regulation of calcium ion transport	61/21092	0.026298186	0.389337206	0.366605554	Gnb5/Nos1	2
GO:0070206	protein trimerization	61/21092	0.026298186	0.389337206	0.366605554	Trim21/Col6a4	2
GO:0006816	calcium ion transport	409/21092	0.027100618	0.389337206	0.366605554	Gnb5/Wfs1/Nos1/Atp2c1/Ibtk	5
GO:2000736	regulation of stem cell differentiation	62/21092	0.027103103	0.389337206	0.366605554	Sox6/Hes5	2
GO:2000756	regulation of peptidyl-lysine acetylation	63/21092	0.027917909	0.389337206	0.366605554	Nos1/Piwil2	2
GO:0001933	negative regulation of protein phosphorylation	414/21092	0.028355965	0.389337206	0.366605554	Cd109/Nos1/Socs5/Hspb1/Ibtk	5

GO:0043949	regulation of cAMP-mediated signaling	64/21092	0.02874251	0.389337206	0.366605554	Cgas/Nos1	2
GO:1901264	carbohydrate derivative transport	64/21092	0.02874251	0.389337206	0.366605554	Slc17a5/Slc35b2	2
GO:0061035	regulation of cartilage development	66/21092	0.030420729	0.389337206	0.366605554	Adamts7/Sox6	2
GO:0090278	negative regulation of peptide hormone secretion	66/21092	0.030420729	0.389337206	0.366605554	Crhbp/Nos1	2
GO:1903034	regulation of response to wounding	165/21092	0.030897152	0.389337206	0.366605554	Cd109/Klk8/Tmprss6	3
GO:0061045	negative regulation of wound healing	67/21092	0.031274163	0.389337206	0.366605554	Cd109/Tmprss6	2
GO:0007051	spindle organization	166/21092	0.031374485	0.389337206	0.366605554	Poc1a/Mlh1/Hspa1b	3
GO:0006163	purine nucleotide metabolic process	426/21092	0.031518336	0.389337206	0.366605554	Guca1b/Nt5e/Guca1a/Nos1/Cox7a2	5
GO:0002262	myeloid cell homeostasis	171/21092	0.033819296	0.389337206	0.366605554	Tmod3/Heph/Sox6	3
GO:0002088	lens development in camera-type eye	70/21092	0.033890674	0.389337206	0.366605554	Bfsp2/Nectin1	2
GO:0043627	response to estrogen	72/21092	0.035680958	0.389337206	0.366605554	Crhbp/Nos1	2
GO:0051851	modification by host of symbiont morphology or physiology	72/21092	0.035680958	0.389337206	0.366605554	Ppib/Tusc2	2
GO:0060271	cilium assembly	301/21092	0.03599202	0.389337206	0.366605554	Poc1a/Mns1/Gmnc/Ulk4	4
GO:0071772	response to BMP	176/21092	0.036360604	0.389337206	0.366605554	Adamts7/Tmprss6/Hes5	3
GO:0071773	cellular response to BMP stimulus	176/21092	0.036360604	0.389337206	0.366605554	Adamts7/Tmprss6/Hes5	3
GO:0060350	endochondral bone morphogenesis	73/21092	0.036589618	0.389337206	0.366605554	Poc1a/Col12a1	2
GO:0034620	cellular response to unfolded protein	74/21092	0.037507172	0.389337206	0.366605554	Wfs1/Hspa1b	2
GO:1901983	regulation of protein acetylation	74/21092	0.037507172	0.389337206	0.366605554	Nos1/Piwil2	2
GO:0061515	myeloid cell development	75/21092	0.038433534	0.389337206	0.366605554	Tmod3/Sox6	2
GO:0051702	interaction with symbiont	77/21092	0.040312336	0.389337206	0.366605554	Ppib/Tusc2	2
GO:0002118	aggressive behavior	10/21092	0.040499085	0.389337206	0.366605554	Crhbp	1
GO:0002934	desmosome organization	10/21092	0.040499085	0.389337206	0.366605554	Nectin1	1
GO:0003091	renal water homeostasis	10/21092	0.040499085	0.389337206	0.366605554	Wfs1	1

GO:0006995	cellular response to nitrogen starvation	10/21092	0.040499085	0.389337206	0.366605554	Nprl2	1
GO:0007220	Notch receptor processing	10/21092	0.040499085	0.389337206	0.366605554	Aph1c	1
GO:0010825	positive regulation of centrosome duplication	10/21092	0.040499085	0.389337206	0.366605554	Poc1a	1
GO:0043562	cellular response to nitrogen levels	10/21092	0.040499085	0.389337206	0.366605554	Nprl2	1
GO:0044154	histone H3-K14 acetylation	10/21092	0.040499085	0.389337206	0.366605554	Piwil2	1
GO:0045607	regulation of inner ear auditory receptor cell differentiation	10/21092	0.040499085	0.389337206	0.366605554	Hes5	1
GO:0045631	regulation of mechanoreceptor differentiation	10/21092	0.040499085	0.389337206	0.366605554	Hes5	1
GO:0046085	adenosine metabolic process	10/21092	0.040499085	0.389337206	0.366605554	Nt5e	1
GO:0048149	behavioral response to ethanol	10/21092	0.040499085	0.389337206	0.366605554	Crhbp	1
GO:0051458	corticotropin secretion	10/21092	0.040499085	0.389337206	0.366605554	Crhbp	1
GO:0051764	actin crosslink formation	10/21092	0.040499085	0.389337206	0.366605554	Baiap2l1	1
GO:0072070	loop of Henle development	10/21092	0.040499085	0.389337206	0.366605554	Hes5	1
GO:0072173	metanephric tubule morphogenesis	10/21092	0.040499085	0.389337206	0.366605554	Hes5	1
GO:0090343	positive regulation of cell aging	10/21092	0.040499085	0.389337206	0.366605554	Cgas	1
GO:1905456	regulation of lymphoid progenitor cell differentiation	10/21092	0.040499085	0.389337206	0.366605554	Hes5	1
GO:2000980	regulation of inner ear receptor cell differentiation	10/21092	0.040499085	0.389337206	0.366605554	Hes5	1
GO:0006473	protein acetylation	184/21092	0.040625775	0.389337206	0.366605554	Morf4l1/Nos1/Piwil2	3
GO:0070838	divalent metal ion transport	457/21092	0.040686115	0.389337206	0.366605554	Gnb5/Wfs1/Nos1/Atp2c1/Ibtk	5
GO:0071277	cellular response to calcium ion	78/21092	0.041264605	0.389337206	0.366605554	Crhbp/Syt12	2
GO:0072511	divalent inorganic cation transport	460/21092	0.041650992	0.389337206	0.366605554	Gnb5/Wfs1/Nos1/Atp2c1/Ibtk	5
GO:0043279	response to alkaloid	79/21092	0.042225339	0.389337206	0.366605554	Crhbp/Nos1	2

GO:0048708	astrocyte differentiation	79/21092	0.042225339	0.389337206	0.366605554	Sox6/Hes5	2
GO:0007218	neuropeptide signaling pathway	80/21092	0.043194454	0.389337206	0.366605554	Npy/Cort	2
GO:0008544	epidermis development	321/21092	0.04385706	0.389337206	0.366605554	Cd109/Ptch2/Atp2c1/Hes5	4
GO:0015748	organophosphate ester transport	81/21092	0.044171866	0.389337206	0.366605554	Stard10/Slc35b2	2
GO:0030203	glycosaminoglycan metabolic process	81/21092	0.044171866	0.389337206	0.366605554	Pxylp1/Itih3	2
GO:0032436	positive regulation of proteasomal ubiquitin-dependent protein catabolic process	81/21092	0.044171866	0.389337206	0.366605554	Socs5/Hspa1b	2
GO:0002756	MyD88-independent toll-like receptor signaling pathway	11/21092	0.044458699	0.389337206	0.366605554	Lrrfip2	1
GO:0006527	arginine catabolic process	11/21092	0.044458699	0.389337206	0.366605554	Nos1	1
GO:0009125	nucleoside monophosphate catabolic process	11/21092	0.044458699	0.389337206	0.366605554	Nt5e	1
GO:0048664	neuron fate determination	11/21092	0.044458699	0.389337206	0.366605554	Hes5	1
GO:0070944	neutrophil mediated killing of bacterium	11/21092	0.044458699	0.389337206	0.366605554	Tusc2	1
GO:0086103	G protein-coupled receptor signaling pathway involved in heart process	11/21092	0.044458699	0.389337206	0.366605554	Nos1	1
GO:0097264	self proteolysis	11/21092	0.044458699	0.389337206	0.366605554	Tmprss6	1
GO:2000615	regulation of histone H3-K9 acetylation	11/21092	0.044458699	0.389337206	0.366605554	Piwil2	1
GO:0072521	purine-containing compound metabolic process	469/21092	0.044628846	0.389337206	0.366605554	Guca1b/Nt5e/Guca1a/Nos1/Cox7a2	5
GO:0048524	positive regulation of viral process	82/21092	0.045157492	0.389337206	0.366605554	Ppib/Trim21	2
GO:0019058	viral life cycle	193/21092	0.045713446	0.389337206	0.366605554	Ppib/Trim21/Nectin1	3
GO:0044782	cilium organization	326/21092	0.045963204	0.389337206	0.366605554	Poc1a/Mns1/Gmnc/Ulk4	4
GO:0048469	cell maturation	194/21092	0.046297442	0.389337206	0.366605554	Bfsp2/Tusc2/Hes5	3
GO:0006862	nucleotide transport	12/21092	0.048402159	0.389337206	0.366605554	Slc35b2	1
GO:0043970	histone H3-K9 acetylation	12/21092	0.048402159	0.389337206	0.366605554	Piwil2	1

GO:0045625	regulation of T-helper 1 cell differentiation	12/21092	0.048402159	0.389337206	0.366605554	Socs5	1
GO:0070986	left/right axis specification	12/21092	0.048402159	0.389337206	0.366605554	Mns1	1
GO:2000674	regulation of type B pancreatic cell apoptotic process	12/21092	0.048402159	0.389337206	0.366605554	Wfs1	1
GO:1902115	regulation of organelle assembly	199/21092	0.049272929	0.389337206	0.366605554	Mns1/Nprl2/Hspa1b	3

Supp. Table S15. List of Biological Processes (BP) at FDR 5% - cerebral cortex

GO ID	Description	BgRatio	pvalue	p.adjust	qvalue	geneID	Count
GO:0003433	chondrocyte development involved in endochondral bone morphogenesis	21/21092	0.000615719	0.139820453	0.11902696	Poc1a/Col12a1	2
GO:0003418	growth plate cartilage chondrocyte differentiation	22/21092	0.000676543	0.139820453	0.11902696	Poc1a/Col12a1	2
GO:0120034	positive regulation of plasma membrane bounded cell projection assembly	106/21092	0.000846561	0.139820453	0.11902696	Mns1/Zmynd10/Rhoq	3
GO:0003413	chondrocyte differentiation involved in endochondral bone morphogenesis	28/21092	0.001099759	0.139820453	0.11902696	Poc1a/Col12a1	2
GO:0045724	positive regulation of cilium assembly	28/21092	0.001099759	0.139820453	0.11902696	Mns1/Zmynd10	2
GO:0070306	lens fiber cell differentiation	29/21092	0.001179919	0.139820453	0.11902696	Bfsp2/Ntrk3	2
GO:0003417	growth plate cartilage development	36/21092	0.001816821	0.154604889	0.131612719	Poc1a/Col12a1	2
GO:0006739	NADP metabolic process	36/21092	0.001816821	0.154604889	0.131612719	Me1/Cyb5r4	2
GO:0007601	visual perception	145/21092	0.002083607	0.154604889	0.131612719	Guca1b/Bfsp2/Cacna2d4	3
GO:0050953	sensory perception of light stimulus	149/21092	0.002251264	0.154604889	0.131612719	Guca1b/Bfsp2/Cacna2d4	3
GO:0003416	endochondral bone growth	43/21092	0.002584086	0.154604889	0.131612719	Poc1a/Col12a1	2
GO:0002063	chondrocyte development	44/21092	0.002704155	0.154604889	0.131612719	Poc1a/Col12a1	2
GO:0098868	bone growth	45/21092	0.002826812	0.154604889	0.131612719	Poc1a/Col12a1	2
GO:0060351	cartilage development involved in endochondral bone morphogenesis	51/21092	0.003616605	0.173279586	0.147510196	Poc1a/Col12a1	2

GO:0009583	detection of light stimulus	52/21092	0.003757129	0.173279586	0.147510196	Guca1b/Cacna2d4	2
GO:0120032	regulation of plasma membrane bounded cell projection assembly	183/21092	0.004019105	0.173279586	0.147510196	Mns1/Zmynd10/Rhoq	3
GO:0060491	regulation of cell projection assembly	185/21092	0.004143112	0.173279586	0.147510196	Mns1/Zmynd10/Rhoq	3
GO:1902017	regulation of cilium assembly	58/21092	0.004652825	0.183786586	0.156454641	Mns1/Zmynd10	2
GO:1902115	regulation of organelle assembly	199/21092	0.005076661	0.189973992	0.161721882	Mns1/Zmynd10/Ntrk3	3
GO:0060348	bone development	220/21092	0.00669866	0.22492988	0.191479282	Poc1a/Col12a1/Ppib	3
GO:0002088	lens development in camera-type eye	70/21092	0.006708555	0.22492988	0.191479282	Bfsp2/Ntrk3	2
GO:0007422	peripheral nervous system development	73/21092	0.007276213	0.22492988	0.191479282	Ntrk3/Egr3	2
GO:0060350	endochondral bone morphogenesis	73/21092	0.007276213	0.22492988	0.191479282	Poc1a/Col12a1	2
GO:1902117	positive regulation of organelle assembly	78/21092	0.008268942	0.231551969	0.197116562	Mns1/Zmynd10	2
GO:0031346	positive regulation of cell projection organization	462/21092	0.00847536	0.231551969	0.197116562	Mns1/Zmynd10/Ntrk3/Rhoq	4
GO:0120031	plasma membrane bounded cell projection assembly	493/21092	0.010583125	0.231551969	0.197116562	Mns1/Poc1a/Zmynd10/Rhoq	4
GO:0006140	regulation of nucleotide metabolic process	97/21092	0.012556103	0.231551969	0.197116562	Guca1b/Me1	2
GO:0060271	cilium assembly	301/21092	0.015630743	0.231551969	0.197116562	Mns1/Poc1a/Zmynd10	3
GO:0060349	bone morphogenesis	115/21092	0.017332275	0.231551969	0.197116562	Poc1a/Col12a1	2
GO:0010825	positive regulation of centrosome duplication	10/21092	0.017408049	0.231551969	0.197116562	Poc1a	1
GO:0031282	regulation of guanylate cyclase activity	10/21092	0.017408049	0.231551969	0.197116562	Guca1b	1
GO:0002062	chondrocyte differentiation	116/21092	0.017617071	0.231551969	0.197116562	Poc1a/Col12a1	2
GO:0002756	MyD88-independent toll-like receptor signaling pathway	11/21092	0.019132549	0.231551969	0.197116562	Lrrfip2	1
GO:0007128	meiotic prophase I	11/21092	0.019132549	0.231551969	0.197116562	Mlh1	1
GO:0019227	neuronal action potential propagation	11/21092	0.019132549	0.231551969	0.197116562	Ntrk3	1
GO:0048680	positive regulation of axon regeneration	11/21092	0.019132549	0.231551969	0.197116562	Ntrk3	1

GO:0051324	prophase	11/21092	0.019132549	0.231551969	0.197116562	Mlh1	1
GO:0098870	action potential propagation	11/21092	0.019132549	0.231551969	0.197116562	Ntrk3	1
GO:0044782	cilium organization	326/21092	0.019289759	0.231551969	0.197116562	Mns1/Poc1a/Zmynd10	3
GO:0009582	detection of abiotic stimulus	125/21092	0.020269212	0.231551969	0.197116562	Guca1b/Cacna2d4	2
GO:0034763	negative regulation of transmembrane transport	125/21092	0.020269212	0.231551969	0.197116562	Gnb5/Rhoq	2
GO:0009581	detection of external stimulus	126/21092	0.020573645	0.231551969	0.197116562	Guca1b/Cacna2d4	2
GO:0000279	M phase	12/21092	0.020854104	0.231551969	0.197116562	Mlh1	1
GO:0070572	positive regulation of neuron projection regeneration	12/21092	0.020854104	0.231551969	0.197116562	Ntrk3	1
GO:0070986	left/right axis specification	12/21092	0.020854104	0.231551969	0.197116562	Mns1	1
GO:0098762	meiotic cell cycle phase	12/21092	0.020854104	0.231551969	0.197116562	Mlh1	1
GO:0098764	meiosis I cell cycle phase	12/21092	0.020854104	0.231551969	0.197116562	Mlh1	1
GO:0062012	regulation of small molecule metabolic process	340/21092	0.02152968	0.231551969	0.197116562	Guca1b/Elovl5/Me1	3
GO:0006596	polyamine biosynthetic process	13/21092	0.022572718	0.231551969	0.197116562	Amd2	1
GO:0016446	somatic hypermutation of immunoglobulin genes	13/21092	0.022572718	0.231551969	0.197116562	Mlh1	1
GO:0042492	gamma-delta T cell differentiation	13/21092	0.022572718	0.231551969	0.197116562	Egr3	1
GO:0042761	very long-chain fatty acid biosynthetic process	13/21092	0.022572718	0.231551969	0.197116562	Elovl5	1
GO:0045141	meiotic telomere clustering	13/21092	0.022572718	0.231551969	0.197116562	Mlh1	1
GO:0002566	somatic diversification of immune receptors via somatic mutation	14/21092	0.024288398	0.231551969	0.197116562	Mlh1	1
GO:0030497	fatty acid elongation	14/21092	0.024288398	0.231551969	0.197116562	Elovl5	1
GO:0036159	inner dynein arm assembly	14/21092	0.024288398	0.231551969	0.197116562	Zmynd10	1
GO:0046607	positive regulation of centrosome cycle	14/21092	0.024288398	0.231551969	0.197116562	Poc1a	1
GO:0070307	lens fiber cell development	14/21092	0.024288398	0.231551969	0.197116562	Bfsp2	1

GO:0090220	chromosome localization to nuclear envelope involved in homologous chromosome segregation	14/21092	0.024288398	0.231551969	0.197116562	Mlh1	1
GO:0099151	regulation of postsynaptic density assembly	14/21092	0.024288398	0.231551969	0.197116562	Ntrk3	1
GO:0046496	nicotinamide nucleotide metabolic process	142/21092	0.025701212	0.231551969	0.197116562	Me1/Cyb5r4	2
GO:0033089	positive regulation of T cell differentiation in thymus	15/21092	0.026001147	0.231551969	0.197116562	Egr3	1
GO:0034397	telomere localization	15/21092	0.026001147	0.231551969	0.197116562	Mlh1	1
GO:0044827	modulation by host of viral genome replication	15/21092	0.026001147	0.231551969	0.197116562	Ppib	1
GO:0048304	positive regulation of isotype switching to IgG isotypes	15/21092	0.026001147	0.231551969	0.197116562	Mlh1	1
GO:1901386	negative regulation of voltage-gated calcium channel activity	15/21092	0.026001147	0.231551969	0.197116562	Gnb5	1
GO:0019362	pyridine nucleotide metabolic process	144/21092	0.026375309	0.231551969	0.197116562	Me1/Cyb5r4	2
GO:0042552	myelination	146/21092	0.027056585	0.231551969	0.197116562	Ntrk3/S100b	2
GO:0003414	chondrocyte morphogenesis involved in endochondral bone morphogenesis	16/21092	0.027710971	0.231551969	0.197116562	Col12a1	1
GO:0003429	growth plate cartilage chondrocyte morphogenesis	16/21092	0.027710971	0.231551969	0.197116562	Col12a1	1
GO:0043968	histone H2A acetylation	16/21092	0.027710971	0.231551969	0.197116562	Morf4l1	1
GO:0044794	positive regulation by host of viral process	16/21092	0.027710971	0.231551969	0.197116562	Ppib	1
GO:0046629	gamma-delta T cell activation	16/21092	0.027710971	0.231551969	0.197116562	Egr3	1
GO:0090171	chondrocyte morphogenesis	16/21092	0.027710971	0.231551969	0.197116562	Col12a1	1
GO:1904889	regulation of excitatory synapse assembly	16/21092	0.027710971	0.231551969	0.197116562	Ntrk3	1
GO:2001015	negative regulation of skeletal muscle cell differentiation	16/21092	0.027710971	0.231551969	0.197116562	S100b	1
GO:0007272	ensheathment of neurons	149/21092	0.028091837	0.231551969	0.197116562	Ntrk3/S100b	2

GO:0008360	regulation of cell shape	149/21092	0.028091837	0.231551969	0.197116562	Rhoq/S100b	2
GO:0008366	axon ensheathment	149/21092	0.028091837	0.231551969	0.197116562	Ntrk3/S100b	2
GO:0072524	pyridine-containing compound metabolic process	149/21092	0.028091837	0.231551969	0.197116562	Me1/Cyb5r4	2
GO:0003422	growth plate cartilage morphogenesis	17/21092	0.029417874	0.231551969	0.197116562	Col12a1	1
GO:0036158	outer dynein arm assembly	17/21092	0.029417874	0.231551969	0.197116562	Zmynd10	1
GO:1904469	positive regulation of tumor necrosis factor secretion	17/21092	0.029417874	0.231551969	0.197116562	Lrrfip2	1
GO:0000712	resolution of meiotic recombination intermediates	18/21092	0.031121862	0.231551969	0.197116562	Mlh1	1
GO:0006595	polyamine metabolic process	18/21092	0.031121862	0.231551969	0.197116562	Amd2	1
GO:0009309	amine biosynthetic process	18/21092	0.031121862	0.231551969	0.197116562	Amd2	1
GO:0031643	positive regulation of myelination	18/21092	0.031121862	0.231551969	0.197116562	S100b	1
GO:0042401	cellular biogenic amine biosynthetic process	18/21092	0.031121862	0.231551969	0.197116562	Amd2	1
GO:0048302	regulation of isotype switching to IgG isotypes	18/21092	0.031121862	0.231551969	0.197116562	Mlh1	1
GO:0048712	negative regulation of astrocyte differentiation	18/21092	0.031121862	0.231551969	0.197116562	Ntrk3	1
GO:0050650	chondroitin sulfate proteoglycan biosynthetic process	18/21092	0.031121862	0.231551969	0.197116562	Pxylp1	1
GO:0099150	regulation of postsynaptic specialization assembly	18/21092	0.031121862	0.231551969	0.197116562	Ntrk3	1
GO:0150052	regulation of postsynapse assembly	18/21092	0.031121862	0.231551969	0.197116562	Ntrk3	1
GO:0006733	oxidoreduction coenzyme metabolic process	160/21092	0.03202188	0.231551969	0.197116562	Me1/Cyb5r4	2
GO:0048291	isotype switching to IgG isotypes	19/21092	0.032822939	0.231551969	0.197116562	Mlh1	1
GO:1905874	regulation of postsynaptic density organization	19/21092	0.032822939	0.231551969	0.197116562	Ntrk3	1
GO:2000251	positive regulation of actin cytoskeleton reorganization	19/21092	0.032822939	0.231551969	0.197116562	Ntrk3	1

GO:0007051	spindle organization	166/21092	0.034252067	0.231551969	0.197116562	Poc1a/Mlh1	2
GO:0043271	negative regulation of ion transport	166/21092	0.034252067	0.231551969	0.197116562	Gnb5/Plcb4	2
GO:0002755	MyD88-dependent toll-like receptor signaling pathway	20/21092	0.034521109	0.231551969	0.197116562	Lrrfip2	1
GO:0010560	positive regulation of glycoprotein biosynthetic process	20/21092	0.034521109	0.231551969	0.197116562	Pxylp1	1
GO:0022403	cell cycle phase	20/21092	0.034521109	0.231551969	0.197116562	Mlh1	1
GO:0032148	activation of protein kinase B activity	20/21092	0.034521109	0.231551969	0.197116562	Ntrk3	1
GO:0046325	negative regulation of glucose import	20/21092	0.034521109	0.231551969	0.197116562	Rhoq	1
GO:0050908	detection of light stimulus involved in visual perception	20/21092	0.034521109	0.231551969	0.197116562	Cacna2d4	1
GO:0050962	detection of light stimulus involved in sensory perception	20/21092	0.034521109	0.231551969	0.197116562	Cacna2d4	1
GO:0015012	heparan sulfate proteoglycan biosynthetic process	21/21092	0.036216379	0.24065276	0.204863923	Pxylp1	1
GO:0000289	nuclear-transcribed mRNA poly(A) tail shortening	22/21092	0.037908752	0.24065288	0.204864026	Mlh1	1
GO:0000413	protein peptidyl-prolyl isomerization	22/21092	0.037908752	0.24065288	0.204864026	Ppib	1
GO:0045723	positive regulation of fatty acid biosynthetic process	22/21092	0.037908752	0.24065288	0.204864026	Elovl5	1
GO:0097107	postsynaptic density assembly	22/21092	0.037908752	0.24065288	0.204864026	Ntrk3	1
GO:1903020	positive regulation of glycoprotein metabolic process	22/21092	0.037908752	0.24065288	0.204864026	Pxylp1	1
GO:0060972	left/right pattern formation	23/21092	0.039598233	0.244743578	0.208346372	Mns1	1
GO:1903077	negative regulation of protein localization to plasma membrane	23/21092	0.039598233	0.244743578	0.208346372	Rhoq	1
GO:0006298	mismatch repair	25/21092	0.042968541	0.244743578	0.208346372	Mlh1	1
GO:0007289	spermatid nucleus differentiation	25/21092	0.042968541	0.244743578	0.208346372	Pygo1	1

GO:0010829	negative regulation of glucose transmembrane transport	25/21092	0.042968541	0.244743578	0.208346372	Rhoq	1
GO:0044788	modulation by host of viral process	25/21092	0.042968541	0.244743578	0.208346372	Ppib	1
GO:1904376	negative regulation of protein localization to cell periphery	25/21092	0.042968541	0.244743578	0.208346372	Rhoq	1
GO:0006312	mitotic recombination	26/21092	0.044649377	0.244743578	0.208346372	Mlh1	1
GO:0030201	heparan sulfate proteoglycan metabolic process	26/21092	0.044649377	0.244743578	0.208346372	Pxylp1	1
GO:0048642	negative regulation of skeletal muscle tissue development	26/21092	0.044649377	0.244743578	0.208346372	S100b	1
GO:0050927	positive regulation of positive chemotaxis	26/21092	0.044649377	0.244743578	0.208346372	Ntrk3	1
GO:1901020	negative regulation of calcium ion transmembrane transporter activity	26/21092	0.044649377	0.244743578	0.208346372	Gnb5	1
GO:1904861	excitatory synapse assembly	26/21092	0.044649377	0.244743578	0.208346372	Ntrk3	1
GO:0035987	endodermal cell differentiation	27/21092	0.04632734	0.244743578	0.208346372	Col12a1	1
GO:0050926	regulation of positive chemotaxis	27/21092	0.04632734	0.244743578	0.208346372	Ntrk3	1
GO:0060536	cartilage morphogenesis	27/21092	0.04632734	0.244743578	0.208346372	Col12a1	1
GO:1904467	regulation of tumor necrosis factor secretion	27/21092	0.04632734	0.244743578	0.208346372	Lrrfip2	1
GO:1990774	tumor necrosis factor secretion	27/21092	0.04632734	0.244743578	0.208346372	Lrrfip2	1
GO:0051307	meiotic chromosome separation	28/21092	0.048002435	0.244743578	0.208346372	Mlh1	1
GO:0042168	heme metabolic process	29/21092	0.049674668	0.244743578	0.208346372	Cyb5r4	1
GO:0046039	GTP metabolic process	29/21092	0.049674668	0.244743578	0.208346372	Rhoq	1
GO:0050654	chondroitin sulfate proteoglycan metabolic process	29/21092	0.049674668	0.244743578	0.208346372	Pxylp1	1
GO:0098698	postsynaptic specialization assembly	29/21092	0.049674668	0.244743578	0.208346372	Ntrk3	1

GO:0006302	double-strand break repair	204/21092	0.049694819	0.244743578	0.208346372	Mlh1/Morf4l1	2
