# Supplementary material for: Inhibition of G-protein signalling in cardiac dysfunction of intellectual developmental disorder with cardiac arrhythmia (IDDCA) syndrome
Source: J Med Genet. 2020 Nov 10;58(12):815–31. doi: 10.1136/jmedgenet-2020-107015 (PMC8639930; doi:10.1136/jmedgenet-2020-107015)
Supplement: Supplementary data [file jmedgenet-2020-107015supp002.pdf]

Supplemental note: Case Reports

Family R

Family R (**Figure S1, Table 1, Supplementary Table S1**) is a Pakistani family from Lahore. The proband is an 8-month-old boy born from healthy consanguineous parents and with a healthy female sibling. He presented with global developmental delay characterized by intellectual disability (ID), severe hypotonia, and altered speech development. Physical examination of this child revealed respiratory stridor, macrocephaly and failure to thrive. The child has passed away in the last months. Cardiologic assessment was not performed prior.

Family S

Family S (**Figure S1, Table 1, Supplementary Table S1**) is a consanguineous Pakistani family from the Multan region with one affected 15-year-old boy who showed global developmental delay and flexor spasm seizures, kept under control with the administration of Valproic Acid. He was non-verbal and presented impaired verbal understanding too. He was hypotonic with no head control. He was thin, and showed nystagmus and gastric reflux.
Electroencephalogram (EEG) demonstrated modified hypsarrhythmia. Magnetic Resonance Imaging (MRI) revealed hypogenesis of the corpus callosum. He experienced episodes of breath holding spell.

Family T

Family T (**Figure S1, Table 1, Supplementary Table S1**) is a consanguineous Pakistani family from Lahore with two affected daughters. The older sister likely died of cardiac arrest. The younger sister is the proband. At 1.2-year of age, she showed global developmental delay, ID, nystagmus and no neck holding. She did not reach developmental milestones regarding lexical

production and verbal understanding. Holter monitoring revealed signs of sick sinus syndrome, with severe bradycardia at rest and prolonged sinus pauses.

Family U

Family U (**Figure S1, Table 1, Supplementary Table S1**) is a consanguineous Pakistani trio family from Lahore with an affected 1 year-old female child. Of the main IDDCA characteristics, the proband displayed ID, inability to hold her neck, and lack of eye contact. She also presented with Fatty Acid Oxidation disorder. MRI showed cerebral atrophy. Dysmorphic features of this individual included prominent philtral ridges, micrognathia, prominent ears and craniofacial disproportion.

Family V

Family V (**Figure S1, Table 1, Supplementary Table S1**) is a consanguineous Pakistani trio family from Lahore with an affected male child showing severe ID, developmental delay, hypotonia and seizures, kept under control with the administration of Epictam. He had no eye contact, no social smile and did not respond to his name. He presented with nystagmus and some dysmoprhic features (blond hair, frontal bossing, epicanthic folds, small palpebral fissure, depressed nasal bridge, high arched palate). Urinary organic acid and plasma amino acids were normal. Holter monitoring revealed a sinus rhythm with insignificant (<3 sec) sinus pauses.

Family W

Family W (**Figure S1, Table 1, Supplementary Table S1**) is a Tunisian family with one non-dysmorphic affected child and two healthy siblings born to consanguineous parents who are

first cousins. The affected 7-year old girl was born at term and weighed 2.9 kg (with a body height of 49 cm, and head circumference of 35 cm). She was delayed in her early developmental milestones and first walked at 31 months; she remains nonverbal with mild intellectual disability (a formal neuropsychological assessment was not performed). Growth parameters at evaluation (5 yo) include height and weight at 2 SD and head circumference at 3 SD. Neurologically, she demonstrated impaired fine motor skills. Both MRI and EEG failed to show anything abnormal. Electrocardiographic monitoring showed sinus-node dysfunction with sinus bradycardia, and prolonged sinus pauses.

At 21 months, she experienced an unexplained coma after a septic shock subsequent to a digestive tract infection. She also had an episode of pyelonephritis, concluded without complications. She had shorter thumbs, but no history of eye disease, nor gastric problems. Metabolic screening reported plasma amino acids imbalances with higher phenylalanine concentrations. Currently, her fine movements progressed with re-education, and she developed sign language; she is enrolled in a special education class.

Family history revealed a non-verbal paternal uncle with severe cognitive impairment with no diagnosis. Additionally, a maternal aunt, a maternal uncle and the son of another maternal aunt presented with severe neurodevelopmental problems, including speech impairment.

Family X

Family X (**Figure S1, Table 1, Supplementary Table S1**) is a consanguineous family from Egypt with one 2 year-old affected boy and two healthy girls. The parents have had a previously affected child who is now deceased (IV.8, Individual 39, **Figure S1, Supplementary Table S1**); his clinical findings were similar to the proband’s phenotype (IV.10, Individual 38, **Figure S1, Supplementary Table S1**). Additionally, three of the couple’s pregnancies resulted in

spontaneous miscarriages (IV.4, IV.7 and IV.9, **Figure S1**). Family history showed an affected cousin (IV.1, Individual 40, **Figure S1, Supplementary Table S1).**

The proband is a nonverbal child with severe ID and mild dysmorphic features including prominent forehead, synophrys, downslanting palpebral fissures, depressed nasal root and low-set ears. He presents with severe hypotonia and hyporeflexia; the child cannot use his hands or stand on his feet, and has no head support. MRI showed prominent ventricular system, mild occipitoparietal patchy white matter lesions, indicating dysmyelination. Additionally, the three affected family members presented retinal disease with mild affection of rods and cones functions. They developed epileptic tonic and focal seizures within the first year of life (7 months for individual 38, 1 year for individual 39 and 9 months for individual 40) treated with valproate and levetiracetam. Comprehensive cardiac evaluation was not carried out in the three individuals of these family.

Family Y

Family Y (**Figure S1, Table 1, Supplementary Table S1**) is a consanguineous Egyptian family with one 19-year-old affected girl and one 16-year-old affected boy. Both siblings are nonverbal individuals displaying severe ID, autistic behaviors, severe hypotonia, mild nystagmus and reduced eye pigmentation. Individual 41 (V.I, **Figure S1, Supplementary Table S1**) presents febrile seizures and an abnormal EEG; seizures were treated with valproate and carbamazepine antiepileptic drugs. Her brother (individual 42, V.II, **Figure S1, Supplementary Table S1**) developed generalized tonic-clonic seizures with cyanosis at 1 year of age; he is on valproate, carbamazepine and levetiracetam antiepileptic treatment. On MRI, cortical deep white matter lesions resulting in defective myelination was noticed; additionally, thin dysplastic corpus callosum and abnormal gyral overfolding resembling polymicrogyria were

observed as well in both siblings. The children present with facial dysmorphisms including long hypotonic face, open mouth, arched eyebrows, mild ptosis, prominent upturned nose, long philtrum, thin lips, broad chin and low set ears. Abnormal ECG testing identified sinus arrhythmias and ectopic premature complexes in individual 41 and 42, respectively.

Family Z

Family Z (**Figure S1, Table 1, Supplementary Table S1**) is a family from Iran with two affected cousins born from healthy consanguineous parents who are in turn first cousins. The children are two boys of 8 (IV.1, individual 43, **Figure S1, Supplementary Table S1**) and 6 years (IV.6, individual 44, **Figure S1, Supplementary Table S1**), respectively, both affected by global developmental delay, severe ID, and epilepsy (treated with phenobarbital until 5 years of age for individual 43 and with topiramate, clobazam and orfiril for individual 44). They presented pathological gastric reflux until 1.5-2 years of age, horizontal nystagmus and strabismus. Tandem Mass Spectrometry revealed an unremarkable metabolic workup in Individual 43. Among other features, Individual 43 presented muscle atrophy, while Individual 44 showed behavioral anomalies including laughing and hand stereotypies. Array CGH (Individual 43) and karyotyping (Individual 44) showed no chromosomal abnormalities. Exhaustive cardiac assessment was not performed in individual 43; echocardiography and cardiac examination at birth were normal in individual 44.
