# Supplementary material for: Inhibition of G-protein signalling in cardiac dysfunction of intellectual developmental disorder with cardiac arrhythmia (IDDCA) syndrome
Source: J Med Genet. 2020 Nov 10;58(12):815–31. doi: 10.1136/jmedgenet-2020-107015 (PMC8639930; doi:10.1136/jmedgenet-2020-107015)
Supplement: Supplementary data [file jmedgenet-2020-107015supp001.pdf]

Appendix: Consortia and networks involved in this study

Full details are available below.

The Synaptopathies and Paroxysmal Syndromes (SYNaPS) Study Group (http://neurogenetics.co.uk/synaptopathies-synaps/):

Stanislav Groppa, Blagovesta Marinova Karashova, Wolfgang Nachbauer, Sylvia Boesch, Larissa Arning, Dagmar Timmann, Bru Cormand, Belen Pérez-Dueñas, Jatinder S. Goraya,, Tipu Sultan, Jun Mine, Daniela Avdjieva, Hadil Kathom, Radka Tincheva, Selina Banu, Mercedes Pineda-Marfa, Pierangelo Veggiotti, Michel D. Ferrari, Arn M. J. M. van den Maagdenberg, Alberto Verrotti, Giangluigi Marseglia, Salvatore Savasta, Mayte García-Silva, Alfons Macaya Ruiz, Barbara Garavaglia, Eugenia Borgione, Simona Portaro, Benigno Monteagudo Sanchez, Richard Boles, Savvas Papacostas, Michail Vikelis, Eleni Zamba Papanicolaou, Efthymios Dardiotis, Shazia Maqbool, Shahnaz Ibrahim, Salman Kirmani, Nuzhat Noureen Rana, Osama Atawneh, George Koutsis, Salvatore Mangano, Carmela Scuderi, Eugenia Borgione, Giovanna Morello, Tanya Stojkovic, Massimo Zollo, Gali Heimer, Yves A. Dauvilliers, Pasquale Striano, Issam Al-Khawaja, Fuad Al-Mutairi, Hamed Sherifa.

Study Group Members:

Prof Stanislav Groppa

Affiliation: Department of Neurology and Neurosurgery, Institute of Emergency Medicine, Chisinau, Republic of Moldova.

Email: sgroppa@gmail.com

De Nittis P, *et al. J Med Genet* 2021; 58:815–831. doi: 10.1136/jmedgenet-2020-107015

Dr. Blagovesta Marinova Karashova

Affiliation: Department of Paediatrics, Medical University of Sofia, Sofia 1431, Bulgaria

Email: blagovestakarashova@gmail.com

Dr. Wolfgang Nachbauer

Affiliation: Department of Neurology, Medical University Innsbruck, Anichstrasse 35, Innsbruck 6020, Austria

Email: Wolfgang.Nachbauer@i-med.ac.at

Prof. Sylvia Boesch

Affiliation: Department of Neurology, Medical University Innsbruck, Anichstrasse 35, Innsbruck 6020, Austria

Email: sylvia.boesch@i-med.ac.at

Dr. Larissa Arning

Affiliation: Department of Human Genetics, Ruhr-University Bochum, Bochum 44801, Germany

Email: Larissa.Arning@ruhr-uni-bochum.de

Prof. Dagmar Timmann

Affiliation: Braun Neurologische Universitätsklinik Universität Essen, Hufelandstr 55, Essen D-45122, Germany

Email: Dagmar.Timmann-Braun@uni-duisburg-essen.de

Prof. Bru Cormand

Affiliation: Department of Genetics, Universitat de Barcelona, Barcelona 08007, Spain

Email: bcormand@ub.edu

Dr. Belen Pérez-Dueñas

Affiliation: Hospital Sant Joan de Deu, Esplugues de Llobregat 08950, Barcelona, Spain

Email: bperez@sjdhospitalbarcelona.org

Dr Gabriella Di Rosa, MD, PhD

Affiliation: Department of Pediatrics, University of Messina, Messina 98123, Italy

Email: gdirosa@unime.it

Prof. Jatinder S. Goraya, MD, FRCP

Affiliation: Division of Paediatric Neurology, Dayanand Medical College & Hospital, Ludhiana, Punjab 141001, India

Email: gorayajs@gmail.com

Prof. Tipu Sultan

Affiliation: Division of Paediatric Neurology, Children's Hospital of Lahore, Lahore 381-D/2, Pakistan

Email: tipusultanmalik@hotmail.com

Prof Jun Mine

Affiliation: Department of Paediatrics, Shimane University, Faculty of Medicine, Izumo, 693-8501, Japan

Email: jmine@med.shimane-u.ac.jp

Prof. Daniela Avdjieva,

Affiliation: Department of Paediatrics, Medical University of Sofia, Sofia 1431, Bulgaria

Email: davdjieva@yahoo.com

Dr. Hadil Kathom,

Affiliation: Department of Pediatrics, Medical University of Sofia, Sofia 1431, Bulgaria

Email: hadilmk@gmail.com

Prof.Dr Radka Tincheva

Affiliation: Head of Department of Clinical Genetics, University Pediatric Hospital, Sofia 1431, Bulgaria

Email: radka.tincheva@gmail.com

Prof. Selina Banu

Affiliation: Neurosciences Unit, Institute of Child Health and Shishu Shastho Foundation Hospital, Mirpur, Dhaka 1216, Bangladesh

Email: selinabanu17@gmail.com

Prof. Mercedes Pineda-Marfa

Affiliation Servei de Neurologia Pediàtrica, l'Hospital Universitari Vall d'Hebron, Barcelona 08035, Spain

Email: pineda@hsjdbcn.org

Prof. Pierangelo Veggiotti

Affiliation: Unit of Infantile Neuropsychiatry Fondazione

Istituto Neurologico "C. Mondino" IRCCS, Via Mondino 2, Pavia 27100, Italy

Email: pierangelo.veggiotti@unipv.it

Prof. Michel D. Ferrari

Affiliation: Leiden University Medical Center, Albinusdreef 2, Leiden 2333, Netherlands

Email: M.D.Ferrari@lumc.nl

Prof. Alberto Verrotti

Affiliation: University of L'Aquila, L'Aquila, Italy

Email: verrottidipianella@univaq.it

Prof Giangluigi Marseglia

Affiliation: Department of Pediatrics, University of Pavia, IRCCS Policlinico "San Matteo", Pavia 27100, Italy

Email: gl.marseglia@smatteo.pv.it

Dr. Salvatore Savasta

Affiliation: Division of Pediatric Neurology, Department of Pediatrics, University of Pavia, IRCCS Policlinico "San Matteo", Pavia 27100, Italy

Email: S.Savasta@smatteo.pv.it

Dr. Mayte García-Silva

Affiliation: Hospital Universitario 12 de Octubre, Madrid 28041, Spain

Email: mgarciasilva@salud.madrid.org

Dr. Alfons Macaya Ruiz

Affiliation: University Hospital Vall d'Hebron, Barcelona 08035, Spain

Email: amacaya@vhebron.net

Prof. Barbara Garavaglia

Affiliation: IRCCS Foundation, Neurological Institute "Carlo Besta", Molecular Neurogenetics, 20126 Milan, Italy

Email: segr.neurogenetica@istituto-besta.it

Dr. Eugenia Borgione

Affiliation: Laboratorio di Neuropatologia Clinica, U.O.S. Malattie, Neuromuscolari Associazione OASI Maria SS. ONLUS – IRCCS, Via Conte Ruggero 73, 94018 Troina, Italy

Email: eborgione@oasi.en.it

Dr. Simona Portaro

De Nittis P, *et al. J Med Genet* 2021; 58:815–831. doi: 10.1136/jmedgenet-2020-107015

Affiliation: IRCCS Centro Neurolesi "Bonino Pulejo", SS113, c.da Casazza, 98124 Messina, Italy

Email: simonaportaro@hotmail.it

Dr. Benigno Monteagudo Sanchez

Affiliation: Hospital Arquitecto Marcide, Avenida de la Residencia S/N, Ferrol (A Coruña), 15401 Spain

Email: benims@hotmail.com

Dr. Richard Boles

Affiliation: Courtagen Life Sciences, 12 Gill Street Suite 3700, Woburn, MA 01801 USA

Email: Richard.Boles@courtagen.com

Prof. Savvas Papacostas

Affiliation: Neurology Clinic B, The Cyprus Institute of Neurology and Genetics, 6 International Airport Road, 1683 Nicosia, Cyprus

Email: savvas@cing.ac.cy

Dr. Michail Vikelis

Affiliation: Iatreio Kefalalgias Glyfadas, 8 Lazaraki str, 3rd floor, 16675, Athens, Greece

Email: mvikelis@headaches.gr

Prof Eleni Zamba Papanicolaou

Affiliation: The Cyprus Institute of Neurology & Genetics, Nicosia, Cyprus

Email: ezamba@cing.ac.cy

Dr Efthymios Dardiotis

Affiliation: UNIVERSITY HOSPITAL OF LARISSA, NEUROLOGY Department, Greece

Email: edar@med.uth.gr

Prof Shazia Maqbool

Affiliation: Department of Developmental and Behavioral Pediatrics, CH&ICH, Lahore, Pakistan

Email: drshazimaq@yahoo.com

Prof Shahnaz Ibrahim

Affiliation: Department of Pediatrics and child health, Aga Khan University, Karachi, Pakistan

Email: shahnaz.ibrahim@aku.edu

Prof Salman Kirmani

Affiliation: Department of Paediatrics & Child Health, The Aga Khan University, Karachi , Pakistan

Email: salman.kirmani@aku.edu

Dr. Nuzhat Noureen Rana

Affiliation: Department of Paediatric Neurology, Children Hospital Complex and ICH, Multan, Pakistan

Email: drnuzhatrana@gmail.com

Dr. Osama Atawneh

Affiliation: Hilal Pediatric Hospital Hebron, Hebron West Bank, Palestine

Email: osamaat@gmail.com

Prof George Koutsis

Dr Marianthi Breza

Affiliation: Neurogenetics Unit, Neurology Department, Eginition Hospital, National and Kapodistrian University, Athens, Greece

Email: marianthibr@med.uoa.gr

Prof Salvatore Mangano

Affiliation: Unità di Neuropsichiatria Infantile, AOUP "P.Giaccone" Palermo, Italy

Email: salvatore.mangano@unipa.it

Dr Carmela Scuderi

Affiliation: Associazione Oasi Maria SS, 94018 Troina, Italy

Email: cscuderi@oasi.en.it

Dr Eugenia Borgione

Affiliation: Associazione Oasi Maria SS, 94018 Troina, Italy

Email: eborgione@oasi.en.it

Dr Giovanna Morello

Affiliation: Institute of Neurological Sciences, National Research Council, Mangone, Italy

De Nittis P, *et al. J Med Genet* 2021; 58:815–831. doi: 10.1136/jmedgenet-2020-107015

Email: g.morello@isn.cnr.it

Dr Tanya Stojkovic

Affiliation: Institute of Myology, Hôpital La Pitié Salpêtrière, Paris, France

Email: stojkovic.tanya@aphp.fr

Prof Massimi Zollo

Affiliation: CEINGE, Biotecnologie Avanzate S.c.a.rl., Naples, Italy

Email: massimo.zollo@unina.it

Dr Gali Heimer

Affiliation: University Hospital of Tel Aviv, Tel Aviv, Israel

Email: galih.md@gmail.com

Prof Yves A. Dauvilliers

Affiliation: University Hospital Montpellier, Montpellier, France

Email: ydauvilliers@yahoo.fr

Prof Pasquale Striano

Affiliation: Institute “Giannina Gaslini”, Genova, Italy

Email: strianop@gmail.com

Dr Issam Al-Khawaja

Affiliation: Albashir University Hospital, Amman, Jordan

Email: isamkhawaja61@gmail.com

Dr Fuad Al-Mutairi

Affiliation: King Saud University, Riyadh, Saudi Arabia

Email: almutairifu@NGHA.MED.SA

Prof Hamed Sherifa

Affiliation: Assiut University Hospital, Assiut, Egypt

Email: hamed_sherifa@yahoo.com
