# Supplementary material for: Inhibition of G-protein signalling in cardiac dysfunction of intellectual developmental disorder with cardiac arrhythmia (IDDCA) syndrome
Source: J Med Genet. 2020 Nov 10;58(12):815–31. doi: 10.1136/jmedgenet-2020-107015 (PMC8639930; doi:10.1136/jmedgenet-2020-107015)
Supplement: Supplementary data [file jmedgenet-2020-107015supp005.pdf]

Appendix: Consortia and networks involved in this study

The Synaptopathies and Paroxysmal Syndromes (SYNaPS) Study Group (http://neurogenetics.co.uk/synaptopathies-synaps/)

Study Group Members:

Prof Stanislav Groppa

Affiliation: Department of Neurology and Neurosurgery, Institute of Emergency Medicine, Chisinau, Republic of Moldova.

Email: sgroppa@gmail.com

Dr. Blagovesta Marinova Karashova

Affiliation: Department of Paediatrics, Medical University of Sofia, Sofia 1431, Bulgaria

Email: blagovestakarashova@gmail.com

Dr. Wolfgang Nachbauer

Affiliation: Department of Neurology, Medical University Innsbruck, Anichstrasse 35, Innsbruck 6020, Austria

Email: Wolfgang.Nachbauer@i-med.ac.at

Prof. Sylvia Boesch

Affiliation: Department of Neurology, Medical University Innsbruck, Anichstrasse 35, Innsbruck 6020, Austria

Email: sylvia.boesch@i-med.ac.at

Dr. Larissa Arning

Affiliation: Department of Human Genetics, Ruhr-University Bochum, Bochum 44801, Germany

Email: Larissa.Arning@ruhr-uni-bochum.de

Prof. Dagmar Timmann

Affiliation: Braun Neurologische Universitätsklinik Universität Essen, Hufelandstr 55, Essen D-45122, Germany

Email: Dagmar.Timmann-Braun@uni-duisburg-essen.de

Prof. Bru Cormand

Affiliation: Department of Genetics, Universitat de Barcelona, Barcelona 08007, Spain

Email: bcormand@ub.edu

Dr. Belen Pérez-Dueñas

Affiliation: Hospital Sant Joan de Deu, Esplugues de Llobregat 08950, Barcelona, Spain

Email: bperez@sjdhospitalbarcelona.org

Dr Gabriella Di Rosa, MD, PhD

Affiliation: Department of Pediatrics, University of Messina, Messina 98123, Italy

Email: gdirosa@unime.it

Prof. Jatinder S. Goraya, MD, FRCP

Affiliation: Division of Paediatric Neurology, Dayanand Medical College & Hospital, Ludhiana, Punjab 141001, India

De Nittis P, *et al. J Med Genet* 2021; 58:815–831. doi: 10.1136/jmedgenet-2020-107015

Email: gorayajs@gmail.com

Prof. Tipu Sultan

Affiliation: Division of Paediatric Neurology, Children's Hospital of Lahore, Lahore 381-D/2, Pakistan

Email: tipusultanmalik@hotmail.com

Prof Jun Mine

Affiliation: Department of Paediatrics, Shimane University, Faculty of Medicine, Izumo, 693-8501, Japan

Email: jmine@med.shimane-u.ac.jp

Prof. Daniela Avdjieva,

Affiliation: Department of Paediatrics, Medical University of Sofia, Sofia 1431, Bulgaria

Email: davdjieva@yahoo.com

Dr. Hadil Kathom,

Affiliation: Department of Pediatrics, Medical University of Sofia, Sofia 1431, Bulgaria

Email: hadilmk@gmail.com

Prof.Dr Radka Tincheva

Affiliation: Head of Department of Clinical Genetics, University Pediatric Hospital, Sofia 1431, Bulgaria

Email: radka.tincheva@gmail.com

Prof. Selina Banu

Affiliation: Neurosciences Unit, Institute of Child Health and Shishu Shastho Foundation Hospital, Mirpur, Dhaka 1216, Bangladesh

Email: selinabanu17@gmail.com

Prof. Mercedes Pineda-Marfa

Affiliation Servei de Neurologia Pediàtrica, l'Hospital Universitari Vall d'Hebron, Barcelona 08035, Spain

Email: pineda@hsjdbcn.org

Prof. Pierangelo Veggiotti

Affiliation: Unit of Infantile Neuropsychiatry Fondazione

Istituto Neurologico "C. Mondino" IRCCS, Via Mondino 2, Pavia 27100, Italy

Email: pierangelo.veggiotti@unipv.it

Prof. Michel D. Ferrari

Affiliation: Leiden University Medical Center, Albinusdreef 2, Leiden 2333, Netherlands

Email: M.D.Ferrari@lumc.nl

Prof. Alberto Verrotti

Affiliation: University of L’Aquila, L’Aquila, Italy

Email: verrottidipianella@univaq.it

Prof Giangluigi Marseglia

Affiliation: Department of Pediatrics, University of Pavia, IRCCS Policlinico "San Matteo", Pavia 27100, Italy

Email: gl.marseglia@smatteo.pv.it

Dr. Salvatore Savasta

Affiliation: Division of Pediatric Neurology, Department of Pediatrics, University of Pavia, IRCCS Policlinico "San Matteo", Pavia 27100, Italy

Email: S.Savasta@smatteo.pv.it

Dr. Mayte García-Silva

Affiliation: Hospital Universitario 12 de Octubre, Madrid 28041, Spain

Email: mgarciasilva@salud.madrid.org

Dr. Alfons Macaya Ruiz

Affiliation: University Hospital Vall d'Hebron, Barcelona 08035, Spain

Email: amacaya@vhebron.net

Prof. Barbara Garavaglia

Affiliation: IRCCS Foundation, Neurological Institute “Carlo Besta”, Molecular Neurogenetics, 20126 Milan, Italy

Email: segr.neurogenetica@istituto-besta.it

Dr. Eugenia Borgione

Affiliation: Laboratorio di Neuropatologia Clinica, U.O.S. Malattie, Neuromuscolari Associazione OASI Maria SS. ONLUS – IRCCS, Via Conte Ruggero 73, 94018 Troina, Italy

Email: eborgione@oasi.en.it

Dr. Simona Portaro

Affiliation: IRCCS Centro Neurolesi "Bonino Pulejo", SS113, c.da Casazza, 98124 Messina, Italy

Email: simonaportaro@hotmail.it

Dr. Benigno Monteagudo Sanchez

Affiliation: Hospital Arquitecto Marcide, Avenida de la Residencia S/N, Ferrol (A Coruña), 15401 Spain

Email: benims@hotmail.com

Dr. Richard Boles

Affiliation: Courtagen Life Sciences, 12 Gill Street Suite 3700, Woburn, MA 01801 USA

Email: Richard.Boles@courtagen.com

Prof. Savvas Papacostas

Affiliation: Neurology Clinic B, The Cyprus Institute of Neurology and Genetics, 6 International Airport Road, 1683 Nicosia, Cyprus

Email: savvas@cing.ac.cy

Dr. Michail Vikelis

De Nittis P, *et al. J Med Genet* 2021; 58:815–831. doi: 10.1136/jmedgenet-2020-107015

Affiliation: Iatreio Kefalalgias Glyfadas, 8 Lazaraki str, 3rd floor, 16675, Athens, Greece

Email: mvikelis@headaches.gr

Prof Eleni Zamba Papanicolaou

Affiliation: The Cyprus Institute of Neurology & Genetics, Nicosia, Cyprus

Email: ezamba@cing.ac.cy

Dr Efthymios Dardiotis

Affiliation: UNIVERSITY HOSPITAL OF LARISSA, NEUROLOGY Department, Greece

Email: edar@med.uth.gr

Prof Shazia Maqbool

Affiliation: Department of Developmental and Behavioral Pediatrics, CH&ICH, Lahore, Pakistan

Email: drshazimaq@yahoo.com

Prof Shahnaz Ibrahim

Affiliation: Department of Pediatrics and child health, Aga Khan University, Karachi, Pakistan

Email: shahnaz.ibrahim@aku.edu

Prof Salman Kirmani

Affiliation: Department of Paediatrics & Child Health, The Aga Khan University, Karachi , Pakistan

Email: salman.kirmani@aku.edu

De Nittis P, *et al. J Med Genet* 2021; 58:815–831. doi: 10.1136/jmedgenet-2020-107015

Dr. Nuzhat Noureen Rana

Affiliation: Department of Paediatric Neurology, Children Hospital Complex and ICH, Multan, Pakistan

Email: drnuzhatrana@gmail.com

Dr. Osama Atawneh

Affiliation: Hilal Pediatric Hospital Hebron, Hebron West Bank, Palestine

Email: osamaat@gmail.com

Prof George Koutsis

Dr Marianthi Breza

Affiliation: Neurogenetics Unit, Neurology Department, Eginition Hospital, National and Kapodistrian University, Athens, Greece

Email: marianthibr@med.uoa.gr

Prof Salvatore Mangano

Affiliation: Unità di Neuropsichiatria Infantile, AOUP "P.Giaccone" Palermo, Italy

Email: salvatore.mangano@unipa.it

Dr Carmela Scuderi

Affiliation: Associazione Oasi Maria SS, 94018 Troina, Italy

Email: cscuderi@oasi.en.it

Dr Eugenia Borgione

Affiliation: Associazione Oasi Maria SS, 94018 Troina, Italy

Email: eborgione@oasi.en.it

Dr Giovanna Morello

Affiliation: Institute of Neurological Sciences, National Research Council, Mangone, Italy

Email: g.morello@isn.cnr.it

Dr Tanya Stojkovic

Affiliation: Institute of Myology, Hôpital La Pitié Salpêtrière, Paris, France

Email: stojkovic.tanya@aphp.fr

Prof Massimi Zollo

Affiliation: CEINGE, Biotecnologie Avanzate S.c.a.rl., Naples, Italy

Email: massimo.zollo@unina.it

Dr Gali Heimer

Affiliation: University Hospital of Tel Aviv, Tel Aviv, Israel

Email: galih.md@gmail.com

Prof Yves A. Dauvilliers

Affiliation: University Hospital Montpellier, Montpellier, France

Email: ydauvilliers@yahoo.fr

Prof Pasquale Striano

Affiliation: Institute “Giannina Gaslini”, Genova, Italy

Email: strianop@gmail.com

Dr Issam Al-Khawaja

Affiliation: Albashir University Hospital, Amman, Jordan

Email: isamkhawaja61@gmail.com

Dr Fuad Al-Mutairi

Affiliation: King Saud University, Riyadh, Saudi Arabia

Email: almutairifu@NGHA.MED.SA

Prof Hamed Sherifa

Affiliation: Assiut University Hospital, Assiut, Egypt

Email: hamed_sherifa@yahoo.com
